# A non-ACE2-blocking neutralizing antibody against Omicron-included SARS-CoV-2 variants

**DOI:** 10.1038/s41392-022-00879-2

**Published:** 2022-01-25

**Authors:** Xiaomin Duan, Rui Shi, Pulan Liu, Qingrui Huang, Fengze Wang, Xinyu Chen, Hui Feng, Weijin Huang, Junyu Xiao, Jinghua Yan

**Affiliations:** 1 CAS Key Laboratory of Pathogenic Microbiology and Immunology, Institute of Microbiology, Chinese Academy of Sciences, Beijing, 100101 China; 2 University of Chinese Academy of Sciences, Beijing, 100049 China; 3 State Key Laboratory of Protein and Plant Gene Research, School of Life Sciences, Peking-Tsinghua Center for Life Sciences, Beijing Advanced Innovation Center for Genomics, Peking University, Beijing, 100871 China; 4 Shanghai Junshi Biosciences Co. Ltd, Shanghai, 200126 China; 5 Division of HIV/AIDS and Sex-transmitted Virus Vaccines, Institute for Biological Product Control, National Institutes for Food and Drug Control (NIFDC), Beijing, 102629 China

**Keywords:** Infection, Infectious diseases

**Dear Editor**,

The emergence of severe acute respiratory syndrome coronavirus-2 (SARS-CoV-2) variants threatens efforts to contain the coronavirus disease 2019 (COVID-19) pandemic. Omicron (B.1.1.529), the fifth novel SARS-CoV-2 variant of concern (VOC), harbors 15 mutations in the receptor-binding domain (RBD) of the spike (S) protein.^1^ These mutations include almost all the sites of existing VOCs (Alpha/B.1.1.7, Beta/B.1.351, Gamma/P.1, and Delta/B.1.617.2).^2–4^ Importantly, the key mutations directly interact with the ACE2 receptor and constitute the main target of neutralizing antibodies (NAbs), which is believed to alter the sensitivity to a large number of NAbs.

To understand how SARS-CoV-2 variants, including Beta, Delta, and Omicron, evade RBD-targeting NAbs, we screened the binding affinities of monoclonal antibodies (mAbs) currently being evaluated in late clinical trials, NAbs with Emergency Use Authorization (EUA), and mAb hu33 to SARS-CoV-2 RBDs. We previously isolated a large panel of SARS-CoV-2 RBD-binding mAbs from RBD mRNA vaccine immunized mice using a 10×Genomics-based antibody discovery platform. The most frequent germline presented by RBD-specific B cells was VH9-3 (15.6%). Among these mAbs, R33 exhibited ultra-potent neutralizing activity and targeted epitopes that located between P2B-2F6 (Class II)^5^ and S309 (Class III) epitope, but do not overlap with the hACE2-binding region. To reduce the risk of a human-anti-mouse-antibody (HAMA) response in clinical trials, R33 was humanized through complementarity determining region (CDR) grafting onto human acceptor germline frameworks and named hu33.^6^ In comparison with the affinity constant (KD) values of ACE2/SARS-CoV-2 RBDs, the Surface Plasmon Resonance (SPR) results demonstrated that Beta and Delta variants partially affected the affinities of NAbs to RBD proteins. Importantly, Omicron fully decreased the binding ability of Class I/II antibodies (LY-CoV16/CB6,^7^ LY-CoV555, REGN10933, CT-59, ADZ1061, ADZ8895, P2C-1F11, and DXP-604) below the affinities of ACE2/Omicron RBD except for S309 (a Class III NAb) and hu33 (Fig. 1a). Using the same set of 11 antibodies, an enzyme-linked immunosorbent assay (ELISA) was employed to measure the blocking potency. Strikingly, Omicron was found to escape the blocking ability of all Class I/II antibodies (EC50 > 60 μg/mL), with the exception of non-ACE2-blocking mAbs hu33 and S309 (Fig. 1b). Furthermore, a cell-based blocking assay positively proved that the hu33 and S309 mAbs did not directly interfere with ACE2 binding (Supplementary Fig. 1).

**Fig. 1:**
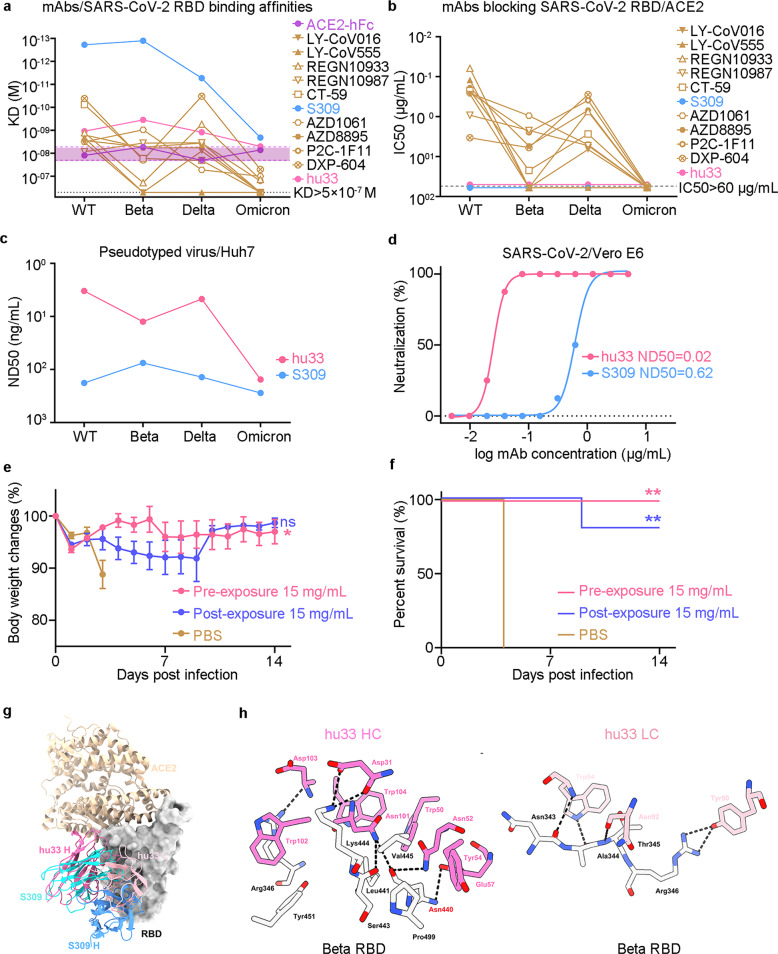
Non-ACE2-blocking mAb hu33 broadly neutralizing SARS-CoV-2 and Omicron variant. **a** Binding affinities of ACE2 or mAbs/SARS-CoV-2 RBD variants. The purple interval represents the threshold (KD) of ACE2/SARS-CoV-2 RBD variants. mAbs binding to SARS-CoV-2 RBD variants are labeled accordingly. **b** Blocking assays of mAbs to ACE2/SARS-CoV-2 RBD variants. The EC50s were calculated by fitting OD450 values from the serially diluted antibody to a sigmoidal dose-response curve. The data presented in (**a**, **b**) are one representative result from three independent experiments. **c** Pseudovirus neutralization analysis of mAbs. SARS-CoV-2 or variants pseudovirus was incubated with serially diluted hu33 or S309. The mixtures were then added to Huh7 cells. ND50 values were calculated by fitting the luciferase activity from serially diluted antibody to a sigmoidal dose-response curve. **d** Authentic SARS-CoV-2 neutralization analysis of mAbs. Mixtures of live SARS-CoV-2 virus and serially diluted hu33 or S309 were added to Vero E6 cells. After a 72-h incubation, ND50 values were calculated by fitting the proportion of cytopathic effect with serially diluted antibody to a sigmoidal dose-response curve. One set of representative data is shown in (**c**) and (**d**). **e**, **f** Groups of 8-week-old hACE2 mice (*n* = 5) were infused with 15 mg/kg mAb hu33 or placebo as a control. Changes of body weight (**e**) and survival curves (**f**) of mice in 2 weeks later by challenging with SARS-CoV-2 virus. Error bars denote SEM (standard error of the mean) of the mean. *P* values were analyzed with ordinary one-way ANOVA in (**e**) (ns *p* > 0.05, and *p < 0.05). *P* values were calculated using the Mantel-Cox test in comparison with that of the PBS-treated group in (**f**) (**p < 0.01). **g** Superimposition of the hu33/SARS-CoV-2 Beta RBD complex (PDB code:7WBH), S309/SARS-CoV-2 RBD (PDB code: 6WPT), and ACE2/SARS-CoV-2 RBD (PDB code: 6LZG) reveals the no stereo-specific competition between NAbs and ACE2. SARS-CoV-2 RBD is shown as surface (gray). hu33-Fab (pink/violet), S309-Fab (blue/cyan), and ACE2 (wheat) are displayed as a cartoon. **h** The binding details between hu33 and Beta RBD are presented with amino acids from VL and VH which forms hydrogen bond interactions with amino acids from Beta RBD. The hydrogen bonds are shown as dashed black lines

S309, which targets conserved epitopes, cross-reacts with and neutralizes all SARS-CoV-2 VOCs and other sarbecoviruses.^8^ To investigate the breadth of hu33 neutralizing activity, SARS-CoV-2 S proteins-bearing vesicular stomatitis virus (VSV delta G/luciferase) pseudoviruses infection of Huh7 cells model was employed. hu33 had shown high potency against prototype SARS-CoV-2 with a median neutralization dose (ND50) of 3.3 ng/mL, consistent with its efficacy of 12.5 and 4.7 ng/mL to Beta and Delta variants, respectively (Fig. 1c and Supplementary Fig. 2a–c). Despite a nearly 46-fold potency reduction compared with that to wild-type SARS-CoV-2, hu33 still neutralized Omicron at an ND50 value of 154.3 ng/mL (Supplementary Fig. 2d). S309 also showed a significantly reduced neutralizing activity against Omicron, with an ND50 value of 276.8 ng/mL (Supplementary Fig. 2d). Of note, hu33 has shown higher neutralization potency than S309 against the pseudotyped prototype and SARS-CoV-2 variants including Omicron. Next, we assessed the neutralizing activity of hu33 against authentic viruses. The Vero E6 cells were infected with the mixture of antibody and SARS-CoV-2 virus in a 96-well plate. Consistently, hu33 potently neutralized live SARS-CoV-2 infection into host cells in a dose-dependent inhibition profile (ND50 = 20 ng/mL), which was one order of magnitude more potent than S309 (Fig. 1d).

In this study, we initially assessed the efficacy of hu33 to protect ACE2 humanized mice from SARS-CoV-2 infection in both prophylactic and treatment settings. K18-hACE2 mice (*n* = 15) were divided into three groups, intraperitoneally administered with 15 mg/kg hu33 or phosphate buffer (PBS) as a placebo, and challenged with 1 × 10^2^ 50% tissue culture infectious dose (TCID50) of the virus through intranasal route. The changes in mice bodyweight of the prophylactic and treatment groups were significantly lower than those of the placebo-treated group (Fig. 1e). Of note, SARS-CoV-2 infection caused a highly lethality (100%) in the control group, whereas hu33 injections provided survival protection against SARS-CoV-2 challenges for 5/5 and 4/5 mice from the prophylactic and treatment groups, respectively (Fig. 1f). These in vivo results demonstrate that NAb hu33 is efficacious in treatment models, as measured by reduced bodyweight changes and infection-induced mortality. Moreover, protection data at pre-exposure settings indicated that hu33 is a promising candidate as a prophylaxis for COVID-19.

To reveal the binding epitope of hu33, we investigated the complex formed by the fragment antigen-binding (Fab) regions of hu33 and a prefusion-stabilized Beta S trimer using single-particle cryo-electron microscopy (cryo-EM), and reconstructed a cryo-EM density map for the one RBD open state at an overall resolution of 3.1 Å (Supplementary Fig. 3 and Supplementary Table 1). The binding site of hu33 on Beta RBD is reminiscent of S309. However, when compared to S309, hu33 moves up towards the RBD neck region that engages ACE2 (Fig. 1g). Further structural analyses suggest that the hu33 Fab binds largely outside of the ACE2 site, although a few clashes may occur between their side chains if they bind together on one RBD (Fig. 1g). RBD residues including Leu441, Val445, Tyr451, and Pro499 form hydrophobic and van der Waals interactions with hu33, whereas Asn343, Ala344, Thr345, Arg346, Asn440, Ser443, and Lys444 are targeted via polar interactions (Fig. 1h). None of the three mutated sites in the Beta RBD (K417N/E484K/N501Y) are involved in interacting with hu33 (Fig. 1h and Supplementary Fig. 4a, b). Among the 15 residues that are mutated in the Omicron RBD, only Gly339 and Asn440 are located in the peripheral areas of the binding epitope (Supplementary Fig. 4b). There is plenty of space between the light chain of hu33 and Gly339 to accommodate an Asp side chain found in Omicron RBD. Moreover, Asn440 forms a hydrogen bond with a negatively charged Glu57 in the heavy chain of hu33 (Fig. 1h), and its mutation to a positively charged Lys may even slightly weaken the binding interaction. The comparisons of binding (Fig. 1a) and neutralizing abilities to the wild-type and Omicron variant (Fig. 1c) reflect that hu33, a Class III NAb, is less sensitive to changes at Asn440 than S309.

In summary, this study showed that the Omicron variant escapes most EUA Class I/II NAbs, whereas the neutralization sensitivity of Class III mAbs, non-ACE2-blocking antibodies, was less affected by this variant. In the meantime, hu33 was a mutation-resistant and broadly neutralizing activity against Omicron-included SARS-CoV-2 variants. Structural and functional analyses support the idea that hu33 is a potential treatment option for treating the SARS-CoV-2 VOCs and combating the COVID-19 pandemic.

## Supplementary information

Supplemental materials

Validation Report

## Data Availability

The atomic models generated from cryo-EM studies of the hu33 and S6P Beta trimer is deposited in the Protein Data Bank under accession codes 7WBH. All data that support the findings of this study are available within the paper, supplementary information, or available from the corresponding author upon reasonable request.
