# Supplementary material for: A non-ACE2-blocking neutralizing antibody against Omicron-included SARS-CoV-2 variants
Source: Signal Transduct Target Ther. 2022 Jan 25;7:23. doi: 10.1038/s41392-022-00879-2 (PMC8787029; doi:10.1038/s41392-022-00879-2)
Supplement: Supplementary file 1 — Supplemental materials [file 41392_2022_879_MOESM1_ESM.docx]

Supplementary Materials for

**A non-ACE2-blocking neutralizing antibody against Omicron-included SARS-CoV-2 variants**

Xiaomin Duan, Rui Shi, Pulan Liu, Qingrui Huang, Fengze Wang, Xinyu Chen, Hui Feng, Weijin Huang, Junyu Xiao and Jinghua Yan

Correspondence to: Weijin Huang(huangweijin@nifdc.org.cn), Junyu Xiao(junyuxiao@pku.edu.cn), Jinghua Yan (yanjh@im.ac.cn)

**This PDF file includes:**

**Supplementary Materials and Methods**

**Supplementary Figures S1-5**

**Supplementary** **Table S1****Materials and Methods**

**Cells and virus**

HEK293T cells (ATCC, CRL-3216), Huh7 cells (3111C0001CCC000679), and Vero E6 cells (ATCC, CRL-1586) were cultured at 37°C under 5% CO_2_ in Dulbecco’s Modified Eagle medium (DMEM) supplemented with 10% fetal bovine serum (FBS). The SARS-CoV-2 virus (hCoV-19/China/CAS-B001/2020, GISAID accession number 25 EPI_ISL_514256-7) used in this study was isolated by Dr. Yuhai Bi and was stored at Biosafety Level 3 at the Institute of Microbiology, Chinese Academy of Sciences.

**Plasmid construction**

The coding sequences of SARS-CoV-2 RBD (residues 319-537, GISAID accession number EPI_ISL_402119), Beta RBD (residues 319-537, GISAID accession number EPI_ISL_7814263), Delta RBD (residues 319-537, GISAID accession number EPI_ISL_7829786), and Omicron RBD (residues 319-537, GISAID accession number EPI_ISL_7834606) were fused with N-terminal native signal peptides and C-terminal 6×His tags and cloned into the pCAGGS expression vector (Addgene) using the EcoRI and XhoI restriction sites. The coding region of human ACE2 (residues 1-805, NCBI accession number BAJ21180) was cloned into a pEGFP-N1 vector plasmid (MiaoLing) using the restriction enzymes XhoI and SmaI. The variable region sequences of hu33, LY-CoV016, LY-CoV555, REGN10933, REGN10987, CT-59, S309, AZD1061, AZD8895, P2C-1F11, and DXP-604 were synthesized with GenScript and fused with the coding sequences of the human IgG_1_ constant region or the corresponding light chain (human IgK or IgL) constant region in a pCAGGS vector. The coding region of human ACE2 (residues 1-615) was fused with the coding sequences of the human IgG_1_ constant region in expression vector pCAGGS (ACE2-hFc). To resolve structure, the SARS-CoV-2 S6P (Beta) expression plasmid containing the spike ectodomain (residues 1-1208, GISAID accession number EPI_ISL_7814263) with a “GSAS” substitution at the furin cleavage site (residues 682-685) and six stabilizing Pro substitutions (F817P, A892P, A899P, A942P, K986P, and V987P) was previously described ^1^.

**Protein expression and purification**

The expression plasmids of RBDs, S6P (Beta), ACE2-hFc, or mAbs were transiently transfected into HEK293T cells. For RBD and S6P (Beta), the supernatant was collected and purified by Ni affinity chromatography using a HisTrap HP 5 mL column (GE Healthcare). The mAbs and ACE2-hFc were purified by HiTrap Protein A HP 5 mL column (GE Healthcare). All proteins were further purified with a Superdex 200 column (GE Healthcare) in 137 mM NaCl, 2.7 mM KCl, 10 mM Na_2_HPO_4_, and 2 mM KH_2_PO_4_ at pH7.4.

**SPR**

Binding affinity was measured by BIAcore 8K system (GE Healthcare). All measurements were performed at 26 °C in a buffer containing 137 mM NaCl, 2.7 mM KCl, 10 mM Na_2_HPO_4_, 2 mM KH_2_PO_4_, and 0.005% Tween-20 at pH7.4. We captured

mAbs or ACE2-hFC on the protein A chip at 1000 response units. Gradient concentrations of RBD protein flowed over the chip. After each cycle, the sensor was regenerated with 10 mM Gly-HCl (pH 1.5). The affinities were calculated using a 1:1 fitting model with BIAevaluation software (GE Healthcare).

**ELISA**

To test the activity of mAbs blocking the binding of hACE2/RBDs, the 96-well microtiter plates (Corning) were coated with recombinant ACE2-hFc protein (500 ng/mL) at 4°C overnight. The plates were blocked with 5% skimmed milk in PBST at 37°C for one hour. A five-fold serially diluted mAbs sample (from 2 mg/mL to 25.6 ng/mL or from 20 μg/mL to 256 pg/mL) and RBD (25, 50, or 100 ng per well) proteins were incubated at 37°C for another hour. After five times of washing, HRP conjugated anti-His tag antibody (1:3000, MBL) was added. After another washing, TMB (Beyotime) was added to the wells for color development. The reactions were stopped with 2 M hydrochloric acid, and the absorbance was measured at 450 nm using a microplate reader (Tecan). When the EC50 is higher than 60 μg/mL, the value is recorded as 60.

**FACS**

The pEGFP-N1-ACE2 expression plasmid was transiently transfected into HEK293T cells. After 24 hours, the cells were collected. The 200 ng/mL RBD protein was mixed with 10 μg/mL hu33, LY-CoV016, or isotype IgG at 37°C for 30 minutes. Then the mixtures were incubated with 3 × 10^5^ cells for another 30 minutes. After washing three times, the cells were incubated with APC-conjugated anti-His antibody (1:200, Miltenyi Biotec). The cells were then analyzed using flow cytometry (BD FACSCanto II).

**Neutralization assay**

For pseudovirus neutralization assay, threefold serial dilutions of the mAbs (initial concentrations at 10, 50, or 120 μg/mL) were mixed with 1.3 × 10^4^ TCID50 pseudotyped SARS-CoV-2 and variants, with two replicates. After incubation at 37℃ for one hour, the mixtures were added to Huh7 cells. After 24 hours of incubation at 37°C in a CO_2_ incubator, the chemiluminescence signals in terms of relative luminescence unit (RLU) values were determined using Glomax Navigator (Promega).

An infectious SARS-CoV-2 neutralization assay was performed. Specifically, 50 μL of twofold serially diluted mAbs (from 5 μg/mL to 4.88 ng/mL) were mixed with 100 TCID50 SARS-CoV-2 in 50 μL, with eight replicates and incubated at 37°C for one hour. Then, 1.5 × 10^4^ Vero E6 cells in an equal volume were added into the mixtures and incubated at 37°C in a CO_2_ incubator. After three days, the cytopathic condition was observed. All experiments involving infectious SARS-CoV-2 followed the standard operating procedures (SOPs) of the approved Biosafety Level-3 facility.

**Animal experiments**

All animal experiments were carried out in accordance with the procedures approved by the Institute of Microbiology, Chinese Academy of Sciences and complied with all relevant ethical regulations regarding animal research.

A total of 15 male K18-hACE2 transgenic mice (eight weeks old, GemPharmaTech) were divided into three groups. In the pre-exposure group, animals received 15 mg/kg antibody one day before the viral challenge. PBS was injected in the placebo group as a control. All of the animals were challenged with 1 ×10^2^ TCID50 SARS-CoV-2. Animals in the postexposure group were given 15 mg/kg antibody eight hours after the viral challenge. The body weight and survival rate of the mice were recorded daily for 14 days.

**Cryo-EM data collection, processing, and structure building**

The samples for cryo-EM study were prepared as previously described ^1,2^. All EM grids were evacuated for 2 minutes and glow-discharged for 30 seconds using a plasma cleaner (Harrick PDC-32G-2). Four microliters of S6P (Beta) protein (0.8 mg/mL) were mixed with the same volume of the hu33 Fab (1 mg/mL), and the mixture was immediately applied to glow-discharged holy-carbon gold grids (Quantifoil, R1.2/1.3). The grids were then blotted with filter paper at 4°C and 100% humidity, and were plunged into the liquid ethane using a Vitrobot Mark IV (FEI). A Titan Krios (operating at 300 kV) equipped with a K3 direct detection camera (Gatan) was used for data collection. A total of 2,367 movie stacks were recorded and processed using cryoSPARC ^3^. A total of 222,776 particles were selected for homogeneous refinement. UCSF Chimera ^4^ and Relion ^5^ were used for generating the mask for local refinement, and the local refinement was performed using cryoSPARC. Coot ^6^ and Phenix ^7^ were used for structural modeling and refinement. USCF ChimeraX ^8^ was used for structural visualization.

**Data analysis and statistics**

Biological replicates and presentation displayed on graphs represent the mean ± SEM. Statistical significance was analyzed using ordinary one-way ANOVA analysis of variance, with multiple comparison tests for multiple groups and a Mantel-Cox log-rank test for survival curves. All analyses were performed using GraphPad Prism 8.1. No data exclusion was performed.

**References**

1 Du, S. *et al.* Structures of SARS-CoV-2 B.1.351 neutralizing antibodies provide insights into cocktail design against concerning variants. *Cell Res.* **31**, 1130-1133, (2021).

2 Du, S. *et al.* Structurally Resolved SARS-CoV-2 Antibody Shows High Efficacy in Severely Infected Hamsters and Provides a Potent Cocktail Pairing Strategy. *Cell*. **183**, 1013-+, (2020).

3 Punjani, A., Rubinstein, J. L., Fleet, D. J. & Brubaker, M. A. cryoSPARC: algorithms for rapid unsupervised cryo-EM structure determination. *Nat. Methods*. **14**, 290-+, (2017).

4 Pettersen, E. F. *et al.* UCSF chimera - A visualization system for exploratory research and analysis. *J. Comput. Chem.* **25**, 1605-1612, (2004).

5 Zivanov, J. *et al.* New tools for automated high-resolution cryo-EM structure determination in RELION-3. *eLife*. **7**, 22, (2018).

6 Emsley, P., Lohkamp, B., Scott, W. G. & Cowtan, K. Features and development of Coot. *Acta Crystallogr. Sect. D-Biol. Crystallogr.* **66**, 486-501, (2010).

7 Liebschner, D. *et al.* Macromolecular structure determination using X-rays, neutrons and electrons: recent developments in Phenix. *Acta Crystallogr. Sect. D-Struct. Biol.* **75**, 861-877, (2019).

8 Pettersen, E. F. *et al.* UCSF ChimeraX: Structure visualization for researchers, educators, and developers. *Protein Sci.* **30**, 70-82, (2021).


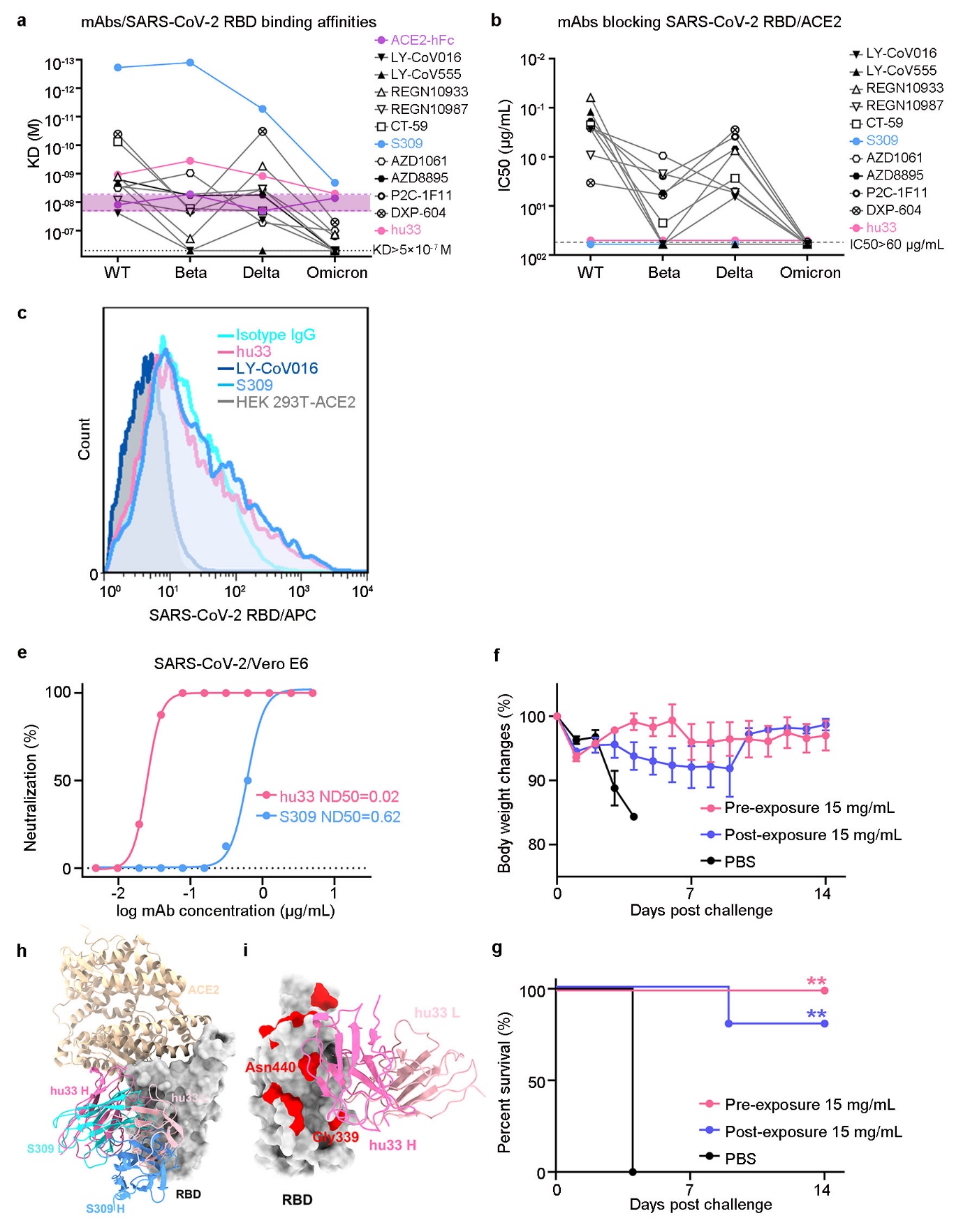
**Figure S1. The hu33 is non-ACE2-blocking mAb.** hACE2 was transiently expressed on the HEK293T cell surface with GFP and cells were stained with SARS-CoV-2 RBD proteins, which were pre-incubated with isotype IgG, hu33, or LY-CoV016.
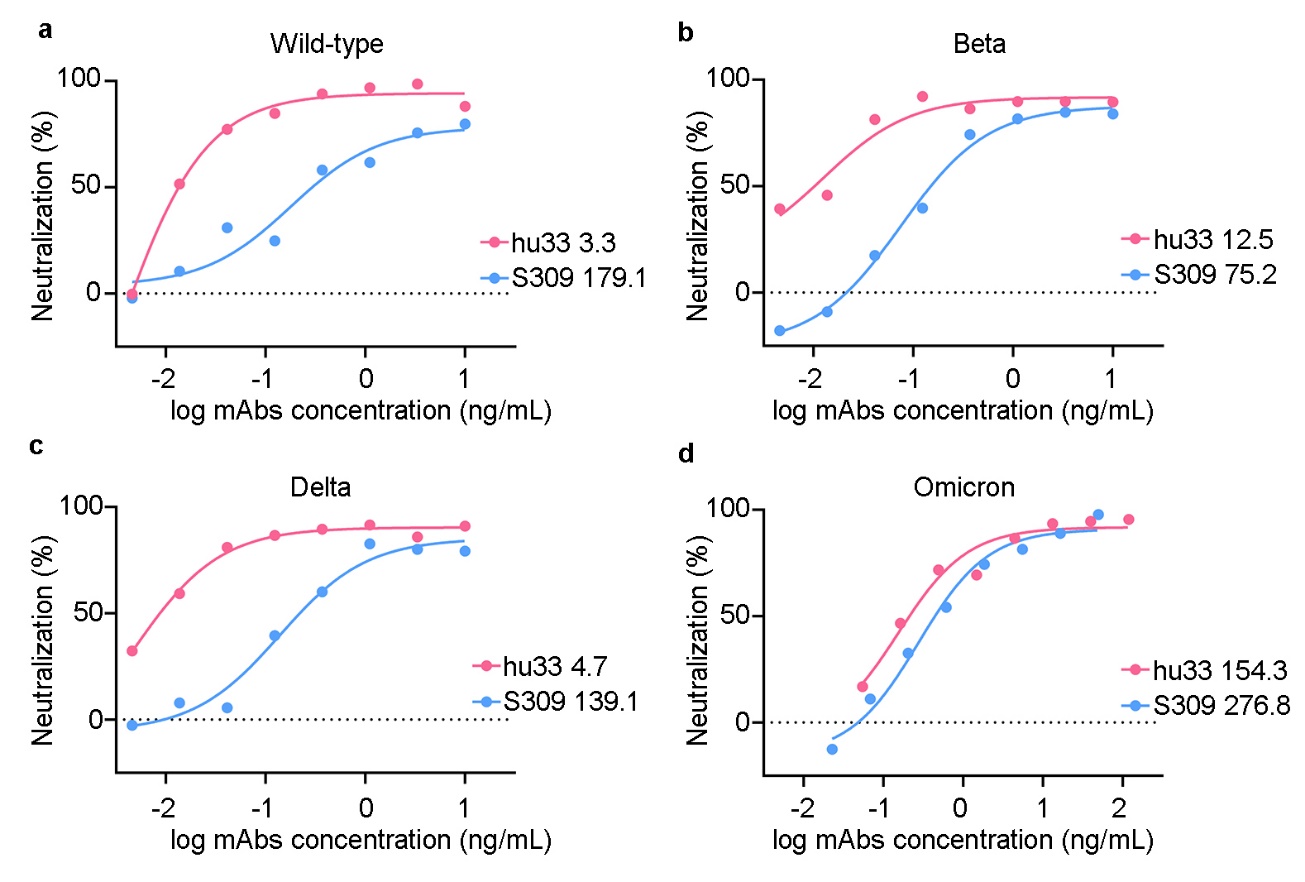
**Figure S2.** **hu33 neutralizes original SARS-CoV-2 and novel lineages of SARS-CoV-2 *in vitro.*** Inhibiting potencies of hu33 and S309 to original SARS-CoV-2, or epidemic SARS-CoV-2 variant pseudoviruses were evaluated in a luciferase reporter assay. ND50 was calculated by fitting the relative fluorescence intensity of host cells from serially diluted antibodies to a sigmoidal dose-response curve.


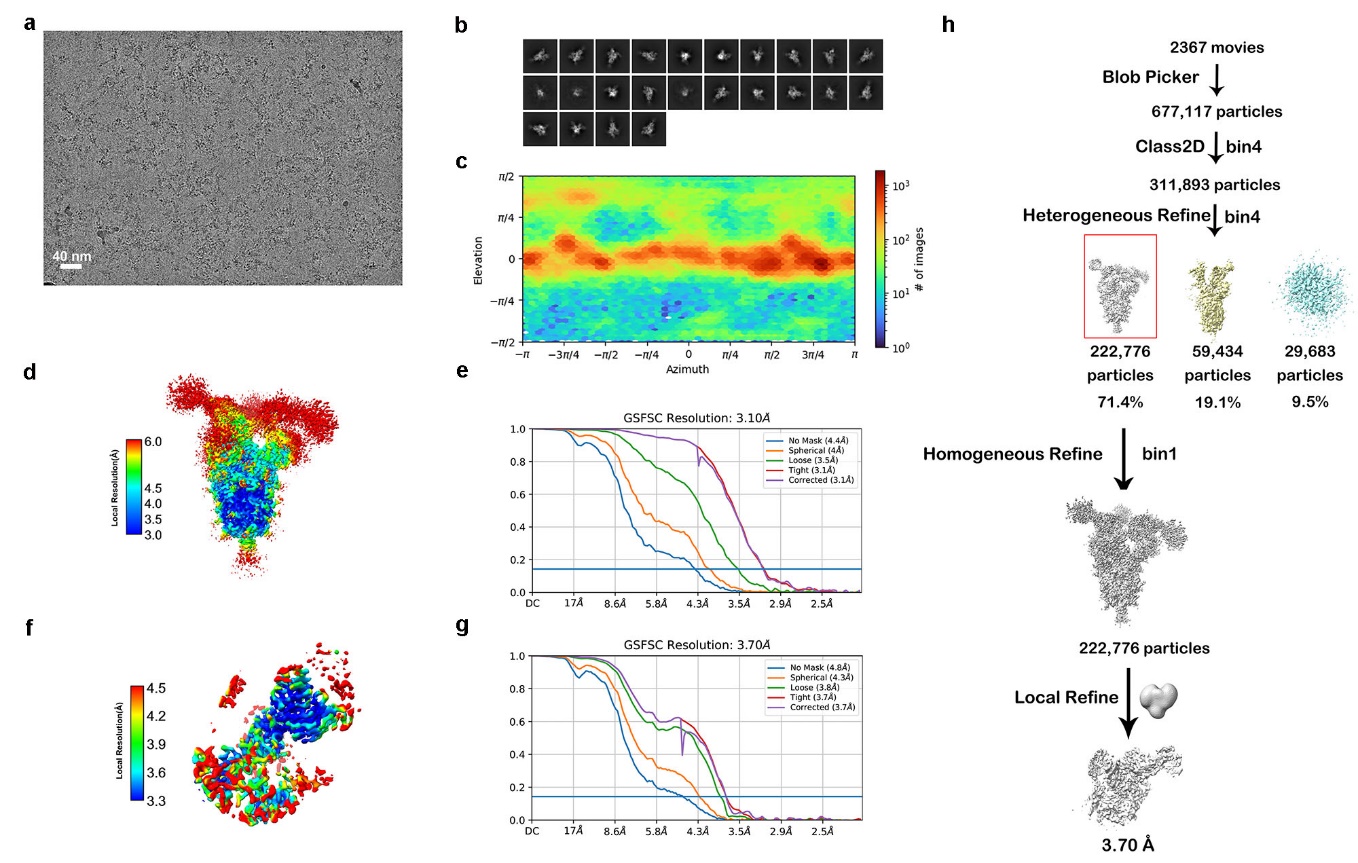
**Figure S3. Workflow for the 3D reconstruction of the cryo-EM structure of the S6P (Beta) trimer in complex with the Fabs of hu33.** **a** A representative raw image was collected using a Titan Krios 300 kV equipped with a K3 detector. **b** Representative 2D classes. **c** Eulerian angle distribution of the particles used in the final 3D reconstruction. **d** Local resolution estimation of the overall density map. **e** Gold standard Fourier shell correlation (FSC) curve with the estimated resolution for the overall density map. **f** Local resolution estimation of the local density map around the region containing the RBD, NTD, and hu33 Fab. **g** Gold standard FSC curve with the estimated resolution for the local density map. **h** Flow chart of image processing.

**
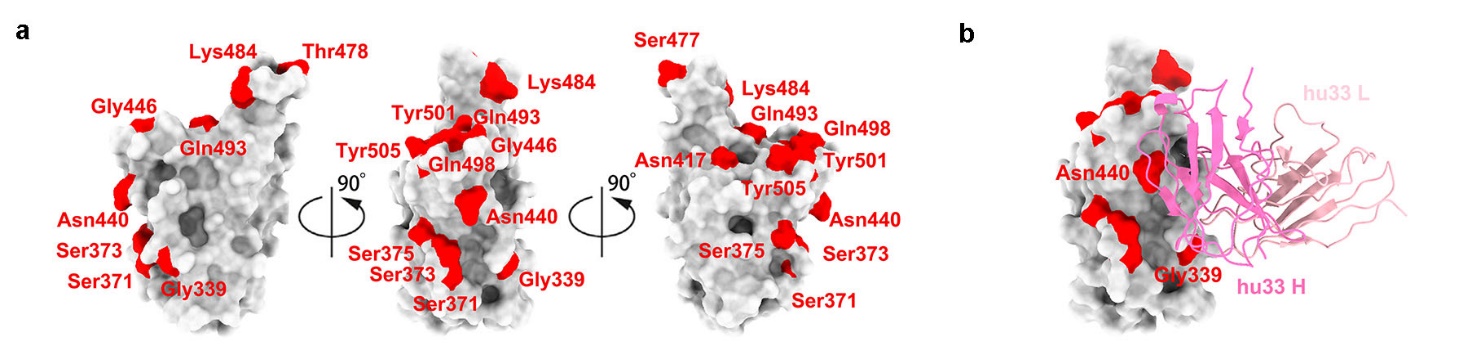
Figure S4. Comparison of key mutated sites of Omicron RBD.** a Mutated sites of Omicron RBD. Beta RBD is rayed as a surface and mutations are highlighted in red. b hu33 Fab is displayed as cartoon. Mutated sites of Omicron RBD are colored in red for emphasis. Residues involved in interactions are labeled.


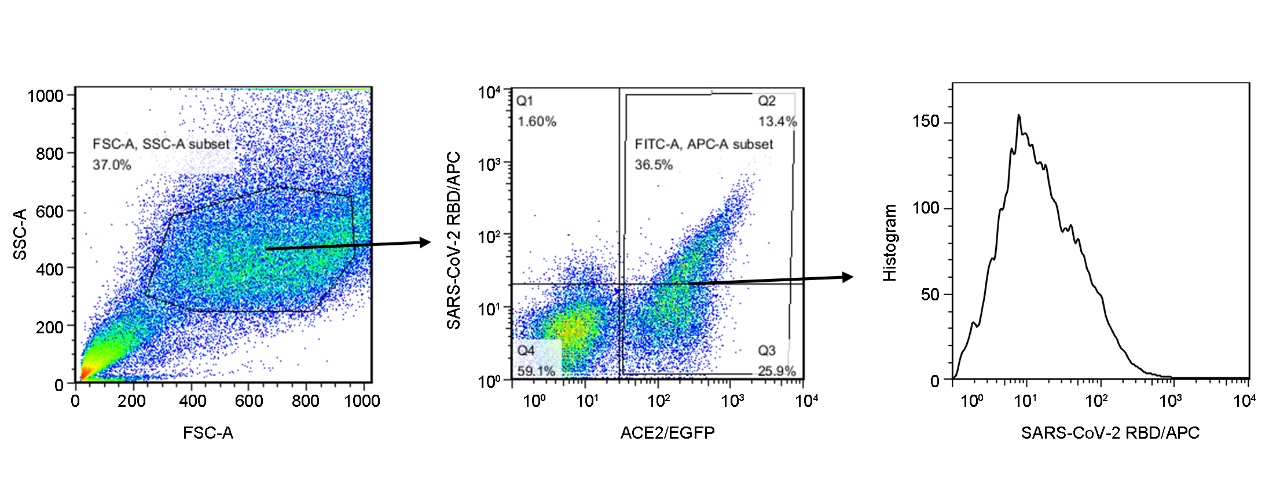
**Figure S5.** **Gating strategy of flow cytometry to assay the blocking functions.** HEK293T-ACE2 cells were stained and analyzed by flow cytometry. The cell was progressively gated to identify single cells and SARS-CoV-2-RBD+ cells as shown in the right panel.

**Table S1. Cryo-EM data collection, processing and validation statistics**

|  | S6P Beta in complex with hu33 Fab |
| --- | --- |
| **Data collection** | |
| Voltage (kV) | 300 |
| Microscope | FEI Titan Krios G3i |
| Camera | K3 (Gatan) |
| Magnification (calibrated) | 64,000X |
| Electron exposure (e^–^/Å^2^) | 50 |
| Exposure rate (e^–^/Å^2^/s) | 19.53 |
| Number of frames collected per micrograph | 32 |
| Energy filter slit width | 20 eV |
| Automation software | EPU |
| Defocus range (μm) | -1.0 to -1.5 |
| Pixel size (Å) | 1.08 |
| **Overall map processing** |  |
| EMDB | EMD-32398 |
| Micrographs used | 2,367 |
| Symmetry imposed | C1 |
| Initial particle images | 677,117 |
| Final particle images | 222,776 |
| Resolution at 0.143 FSC of masked reconstruction (Å) | 3.10 |
| Map sharpening B factor (Å^2^) | -70.5 |
| **Local map processing** |  |
| EMDB | EMD-32395 |
| Final particle images | 222,776 |
| Resolution at 0.143 FSC of masked reconstruction (Å) | 3.75 |
| Map sharpening B factor (Å^2^) | -117.0 |
| **Overall Refinement** |  |
| PDB | 7WBH |
| Initial model used (PDB code) | 7CHH/7CHF |
| Refinement package | Phenix v1.18 (Real-space refinement at 3.13 Å) |
| Map-model CC |  |
| CC_mask | 0.53 |
| CC_box | 0.60 |
| CC_peaks | 0.45 |
| CC_volume | 0.53 |
| Model composition |  |
| Non-hydrogen atoms | 29,187 |
| Protein residues | 3,681 |
| Ligands | BMA:3 NAG:52 |
| R.m.s. deviations |  |
| Bond lengths (Å) | 0.016 |
| Bond angles (°) | 1.506 |
| *B* factors (Å^2^) |  |
| Protein | 189.66 |
| Ligands | 150.08 |
| Validation |  |
| MolProbity score | 2.92 |
| Clashscore | 15.11 |
| Poor rotamers (%) | 7.89 |
| Ramachandran plot |  |
| Favored (%) | 89.71 |
| Allowed (%) | 9.27 |
| Disallowed (%) | 0.00 |
| Cβ outliers (%) | 0.15 |
| CaBLAM outliers (%) | 5.27 |
| **Local Refinement** |  |
| PDB | 7WB5 |
| Initial model used (PDB code) | 7CHH/7CHF |
| Refinement package | Phenix v1.18 (Real-space refinement at 3.75 Å) |
| Map-model CC |  |
| CC_mask | 0.78 |
| CC_box | 0.61 |
| CC_peaks | 0.48 |
| CC_volume | 0.76 |
| Model composition |  |
| Non-hydrogen atoms | 3,256 |
| Protein residues | 408 |
| Ligands | BMA:1 NAG:2 |
| R.m.s. deviations |  |
| Bond lengths (Å) | 0.011 |
| Bond angles (°) | 1.458 |
| *B* factors (Å^2^) |  |
| Protein | 110.06 |
| Ligands | 30.00 |
| Validation |  |
| MolProbity score | 3.36 |
| Clashscore | 17.76 |
| Poor rotamers (%) | 0 |
| Ramachandran plot |  |
| Favored (%) | 79.60 |
| Allowed (%) | 18.16 |
| Disallowed (%) | 0.00 |
| Cβ outliers (%) | 0.00 |
| CaBLAM outliers (%) | 9.09 |
