# Supplementary material for: A non-ACE2-blocking neutralizing antibody against Omicron-included SARS-CoV-2 variants
Source: Signal Transduct Target Ther. 2022 Jan 25;7:23. doi: 10.1038/s41392-022-00879-2 (PMC8787029; doi:10.1038/s41392-022-00879-2)
Supplement: Supplementary file 2 — Validation Report [file 41392_2022_879_MOESM2_ESM.pdf]

Preliminary Full wwPDB EM Validation Report (i)

Dec 17, 2021 – 05:13 PM JST

Deposition ID : D_1300026375

This is a Preliminary Full wwPDB EM Validation Report.

This report is produced by the wwPDB Deposition System during initial deposition but before annotation of the structure.

We welcome your comments at *validation@mail.wwpdb.org*
A user guide is available at
https://www.wwpdb.org/validation/2017/EMValidationReportHelp
with specific help available everywhere you see the (i) symbol.

PRELIMINARY VALIDATION REPORT

The following versions of software and data (see references (i)) were used in the production of this report:

EMDB validation analysis	:	0.0.0.dev97
Mogul	:	1.8.5 (274361), CSD as541be (2020)
MolProbity	:	4.02b-467
Percentile statistics	:	20191225.v01 (using entries in the PDB archive December 25th 2019)
Ideal geometry (proteins)	:	Engh & Huber (2001)
Ideal geometry (DNA, RNA)	:	Parkinson et al. (1996)
Validation Pipeline (wwPDB-VP)	:	2.24

1 Overall quality at a glance (i)

The following experimental techniques were used to determine the structure:
ELECTRON MICROSCOPY

The reported resolution of this entry is 3.70 Å.

Percentile scores (ranging between 0-100) for global validation metrics of the entry are shown in the following graphic. The table shows the number of entries on which the scores are based.

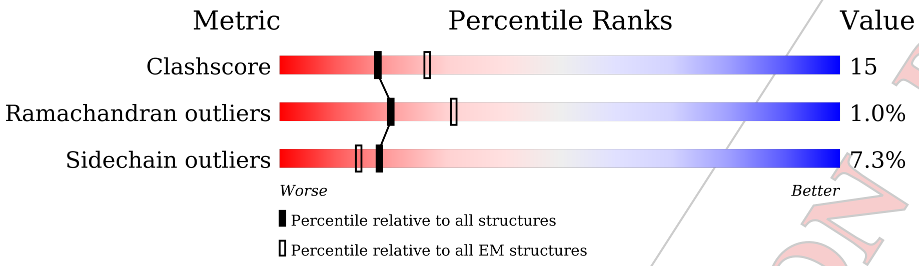

Metric	Whole archive (#Entries)	EM structures (#Entries)
Clashscore	158937	4297
Ramachandran outliers	154571	4023
Sidechain outliers	154315	3826

The table below summarises the geometric issues observed across the polymeric chains and their fit to the map. The red, orange, yellow and green segments of the bar indicate the fraction of residues that contain outliers for >=3, 2, 1 and 0 types of geometric quality criteria respectively. A grey segment represents the fraction of residues that are not modelled. The numeric value for each fraction is indicated below the corresponding segment, with a dot representing fractions <=5% The upper red bar (where present) indicates the fraction of residues that have poor fit to the EM map (all-atom inclusion < 40%). The numeric value is given above the bar.

Mol	Chain	Length	Quality of chain
1	A	1016	19%<br>76% 20% • •
2	B	1000	13%<br>76% 21% •
3	C	1000	14%<br>74% 23% •
4	O	118	52%<br>58% 42% •
4	P	118	34%<br>58% 42% •
4	U	118	78%<br>57% 42% •
5	Q	107	56%<br>61% 38% •
5	S	107	41%<br>61% 38% •

Continued on next page...

PRELIMINARY VALIDATION REPORT

Continued from previous page...

Mol	Chain	Length	Quality of chain
5	V	107	84%<br>59% 40% •
6	D	3	67%<br>33% 67%
6	E	3	33%<br>33% 67%
6	F	3	33%<br>33% 67%
7	G	2	50%<br>50% 50%
7	H	2	100%
7	I	2	50% 50%
7	J	2	50%<br>50% 50%
7	K	2	50%<br>100%
7	L	2	100%
7	M	2	50%<br>100%
7	N	2	100%
7	R	2	50% 50%
7	T	2	50%<br>100%

PRELIMINARY VALIDATION REPORT

2 Entry composition (i)

There are 8 unique types of molecules in this entry. The entry contains 29187 atoms, of which 0 are hydrogens and 0 are deuteriums.

In the tables below, the AltConf column contains the number of residues with at least one atom in alternate conformation and the Trace column contains the number of residues modelled with at most 2 atoms.

- Molecule 1 is a protein called spike chain A.

Mol	Chain	Residues	Atoms					AltConf	Trace
			Total	C	N	O	S		
1	A	1006	7761	4956	1292	1478	35	0	0

- Molecule 2 is a protein called chain B of spike.

Mol	Chain	Residues	Atoms					AltConf	Trace
			Total	C	N	O	S		
2	B	1000	7732	4938	1283	1476	35	0	0

- Molecule 3 is a protein called chain C of spike.

Mol	Chain	Residues	Atoms					AltConf	Trace
			Total	C	N	O	S		
3	C	1000	7677	4903	1270	1469	35	0	0

- Molecule 4 is a protein called heavy chain of R33.

Mol	Chain	Residues	Atoms					AltConf	Trace
			Total	C	N	O	S		
4	U	118	928	594	151	178	5	0	0
4	O	118	928	594	151	178	5	0	0
4	P	118	928	594	151	178	5	0	0

- Molecule 5 is a protein called light chain of R33.

Mol	Chain	Residues	Atoms					AltConf	Trace
			Total	C	N	O	S		
5	V	107	824	520	137	164	3	0	0
5	Q	107	824	520	137	164	3	0	0

Continued on next page...

PRELIMINARY VALIDATION REPORT

Continued from previous page...

Mol	Chain	Residues	Atoms	AltConf	Trace
5	S	107	Total C N O S<br>824 520 137 164 3	0	0

- Molecule 6 is an oligosaccharide called beta-D-mannopyranose-(1-4)-2-acetamido-2-deoxy-beta-D-glucopyranose-(1-4)-2-acetamido-2-deoxy-beta-D-glucopyranose.

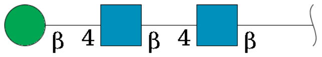

Mol	Chain	Residues	Atoms	AltConf	Trace
6	D	3	Total C N O<br>39 22 2 15	0	0
6	E	3	Total C N O<br>39 22 2 15	0	0
6	F	3	Total C N O<br>39 22 2 15	0	0

- Molecule 7 is an oligosaccharide called 2-acetamido-2-deoxy-beta-D-glucopyranose-(1-4)-2-acetamido-2-deoxy-beta-D-glucopyranose.

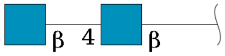

Mol	Chain	Residues	Atoms	AltConf	Trace
7	G	2	Total C N O<br>28 16 2 10	0	0
7	H	2	Total C N O<br>28 16 2 10	0	0
7	I	2	Total C N O<br>28 16 2 10	0	0
7	J	2	Total C N O<br>28 16 2 10	0	0
7	K	2	Total C N O<br>28 16 2 10	0	0
7	L	2	Total C N O<br>28 16 2 10	0	0
7	M	2	Total C N O<br>28 16 2 10	0	0
7	N	2	Total C N O<br>28 16 2 10	0	0

Continued on next page...

PRELIMINARY VALIDATION REPORT

Continued from previous page...

Mol	Chain	Residues	Atoms	AltConf	Trace
7	R	2	Total C N O<br>28 16 2 10	0	0
7	T	2	Total C N O<br>28 16 2 10	0	0

- Molecule 8 is 2-acetamido-2-deoxy-beta-D-glucopyranose (three-letter code: NAG) (formula: $C_8H_{15}NO_6$).

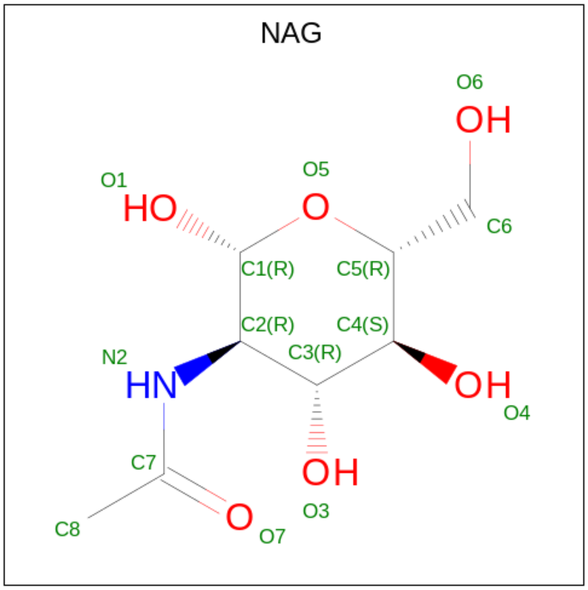

Mol	Chain	Residues	Atoms	AltConf
8	A	1	Total C N O<br>98 56 7 35	0
8	A	1	Total C N O<br>98 56 7 35	0
8	A	1	Total C N O<br>98 56 7 35	0
8	A	1	Total C N O<br>98 56 7 35	0
8	A	1	Total C N O<br>98 56 7 35	0
8	A	1	Total C N O<br>98 56 7 35	0
8	A	1	Total C N O<br>98 56 7 35	0
8	B	1	Total C N O<br>154 88 11 55	0
8	B	1	Total C N O<br>154 88 11 55	0

Continued on next page...

PRELIMINARY VALIDATION REPORT

Continued from previous page...

Mol	Chain	Residues	Atoms	AltConf
8	B	1	Total C N O<br>154 88 11 55	0
8	B	1	Total C N O<br>154 88 11 55	0
8	B	1	Total C N O<br>154 88 11 55	0
8	B	1	Total C N O<br>154 88 11 55	0
8	B	1	Total C N O<br>154 88 11 55	0
8	B	1	Total C N O<br>154 88 11 55	0
8	B	1	Total C N O<br>154 88 11 55	0
8	B	1	Total C N O<br>154 88 11 55	0
8	B	1	Total C N O<br>154 88 11 55	0
8	C	1	Total C N O<br>112 64 8 40	0
8	C	1	Total C N O<br>112 64 8 40	0
8	C	1	Total C N O<br>112 64 8 40	0
8	C	1	Total C N O<br>112 64 8 40	0
8	C	1	Total C N O<br>112 64 8 40	0
8	C	1	Total C N O<br>112 64 8 40	0
8	C	1	Total C N O<br>112 64 8 40	0
8	C	1	Total C N O<br>112 64 8 40	0

PRELIMINARY VALIDATION REPORT

3 Residue-property plots (i)

These plots are drawn for all protein, RNA, DNA and oligosaccharide chains in the entry. The first graphic for a chain summarises the proportions of the various outlier classes displayed in the second graphic. The second graphic shows the sequence view annotated by issues in geometry and atom inclusion in map density. Residues are color-coded according to the number of geometric quality criteria for which they contain at least one outlier: green = 0, yellow = 1, orange = 2 and red = 3 or more. A red diamond above a residue indicates a poor fit to the EM map for this residue (all-atom inclusion < 40%). Stretches of 2 or more consecutive residues without any outlier are shown as a green connector. Residues present in the sample, but not in the model, are shown in grey.

- Molecule 1: spike chain A

Chain A:

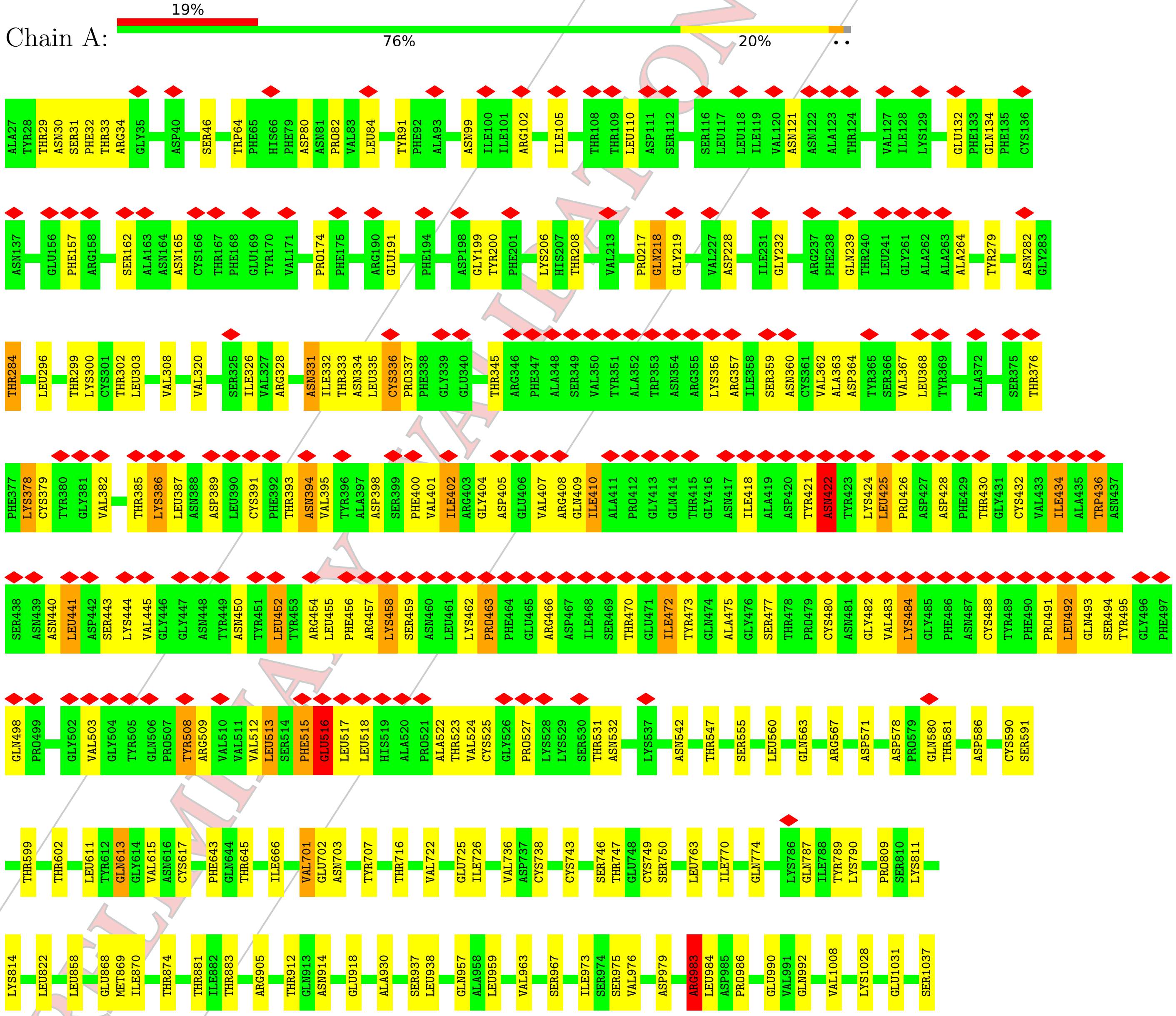

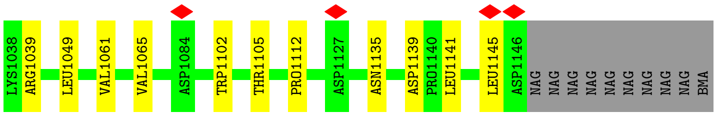

• Molecule 2: chain B of spike

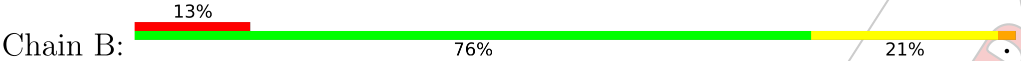

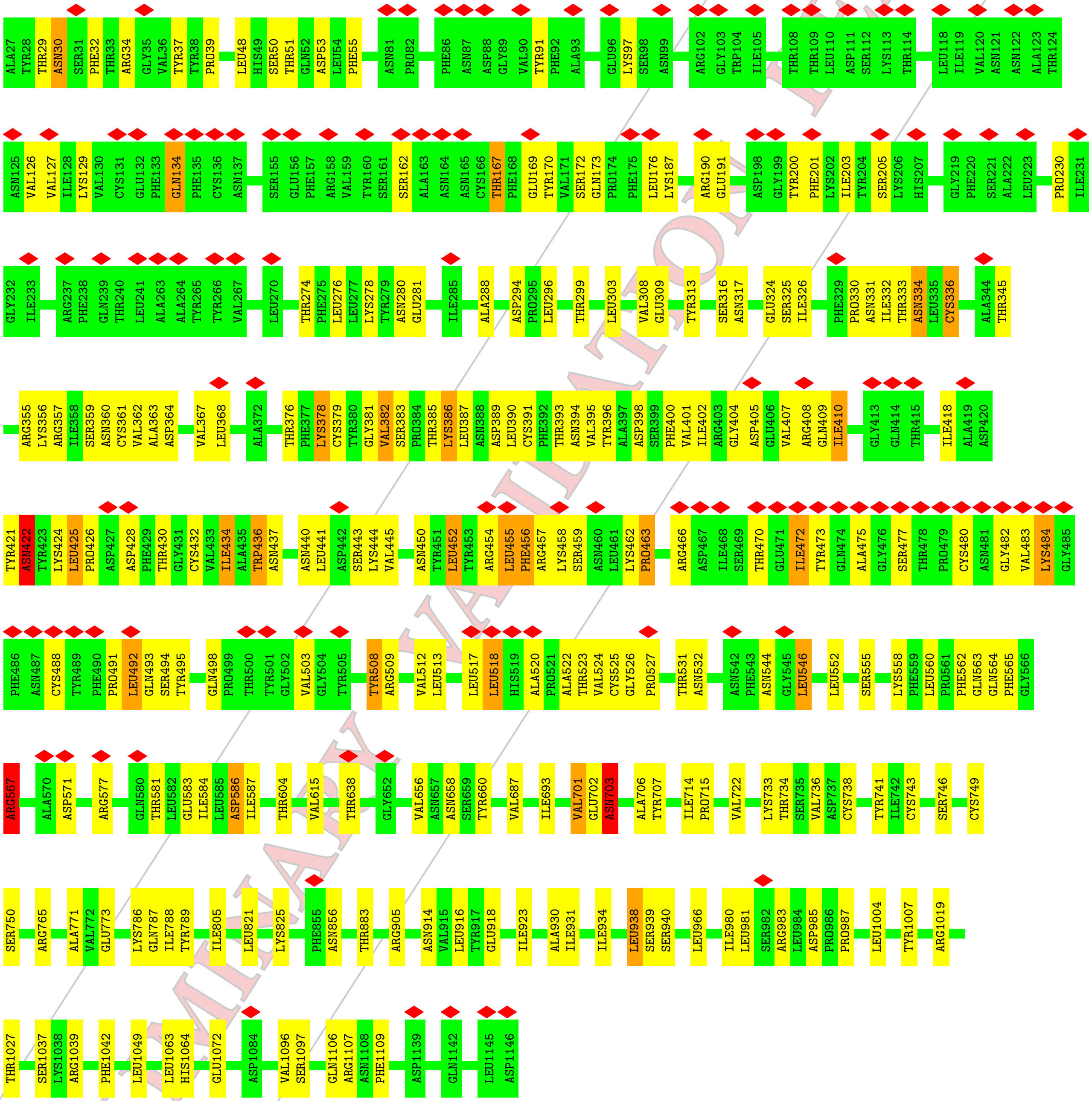

• Molecule 3: chain C of spike

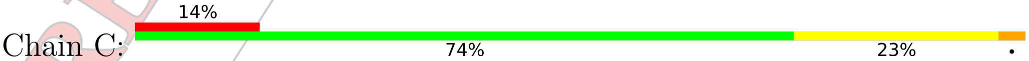

PRELIMINARY VALIDATION REPORT

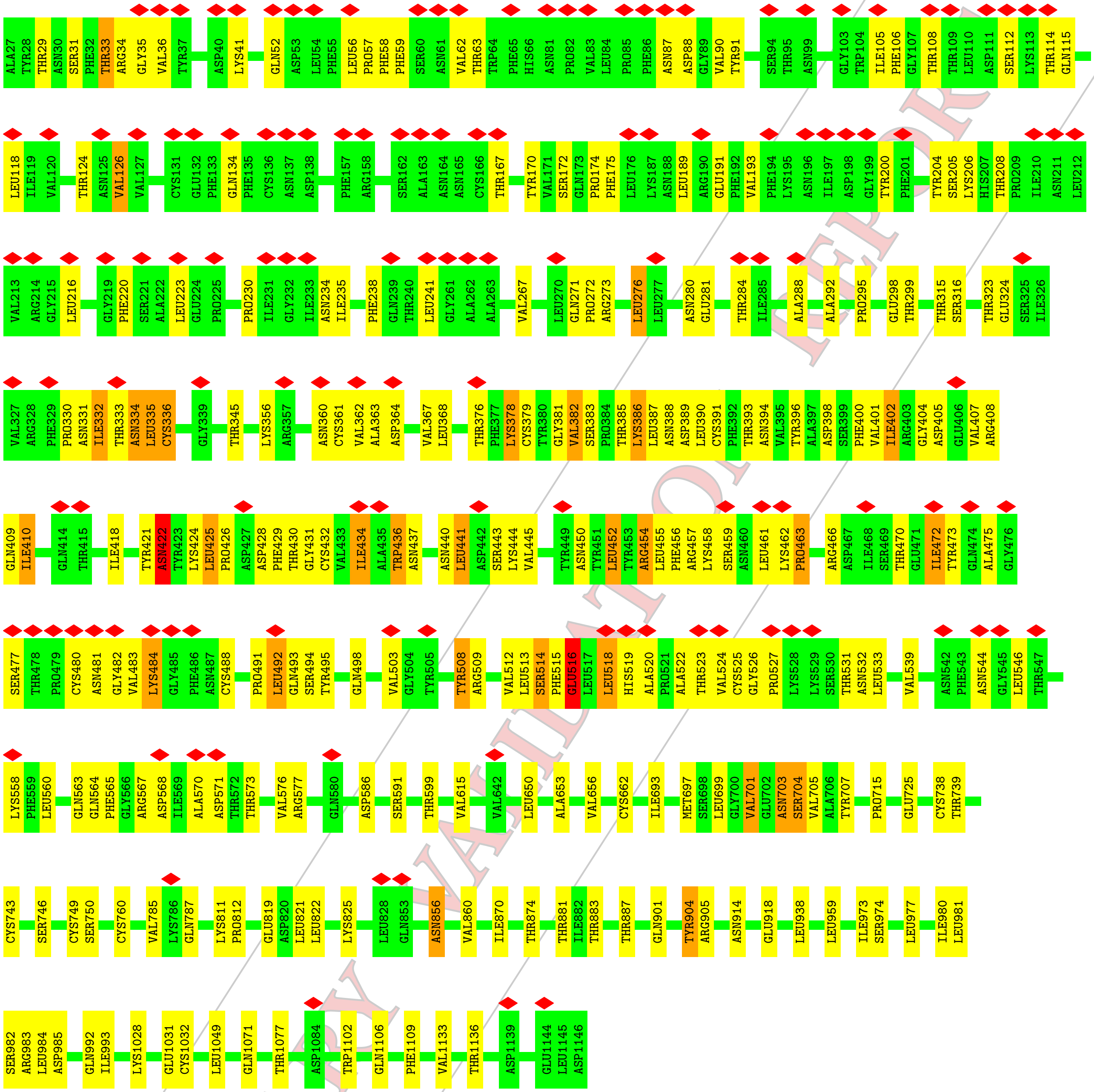

- Molecule 4: heavy chain of R33

Chain U:

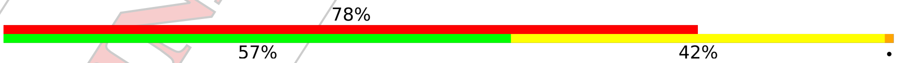

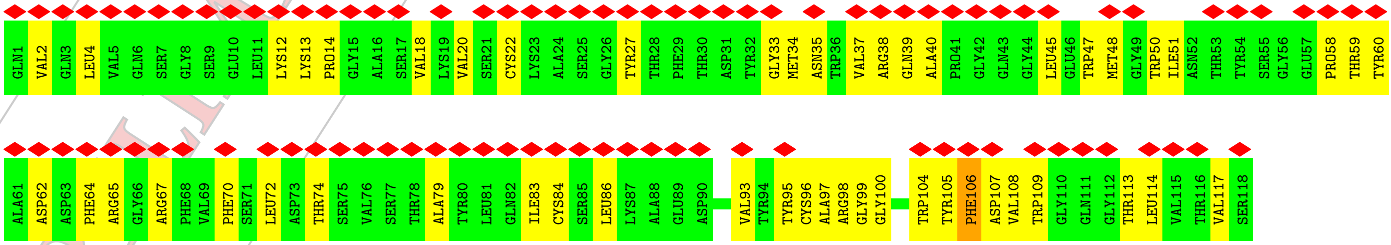

- Molecule 4: heavy chain of R33

PRELIMINARY VALIDATION REPORT

Chain O:

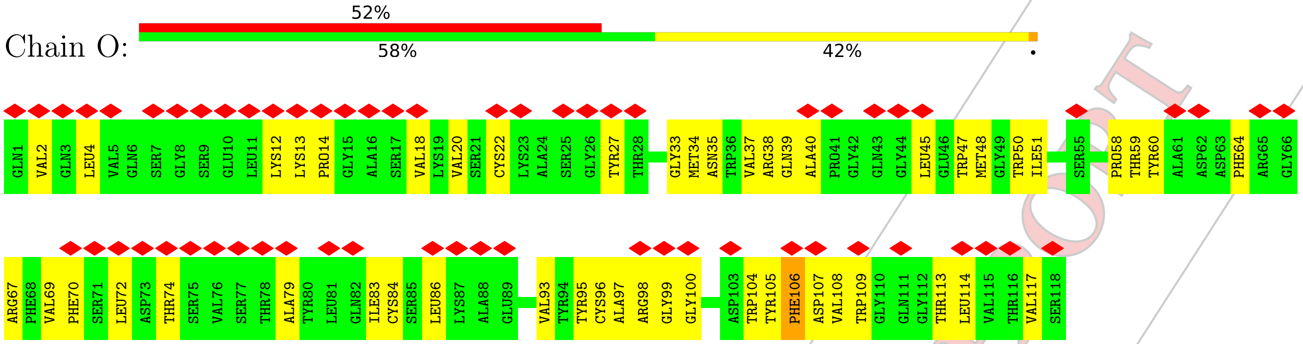

• Molecule 4: heavy chain of R33

Chain P:

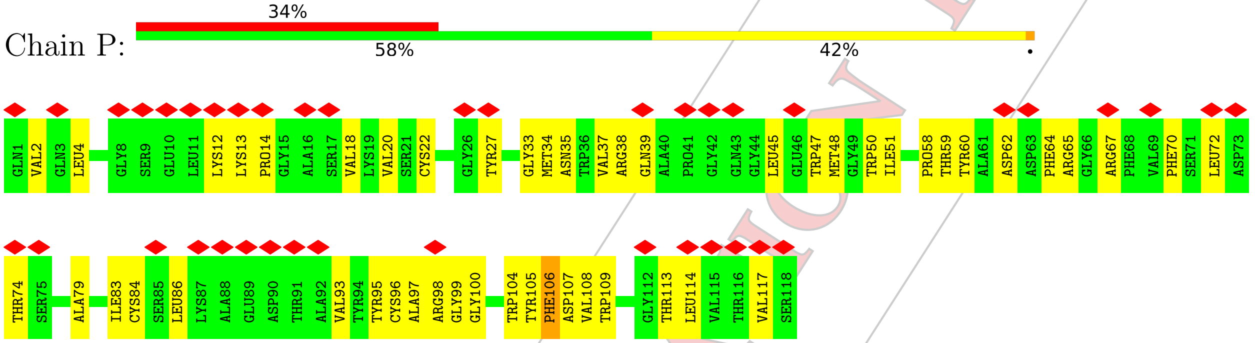

• Molecule 5: light chain of R33

Chain V:

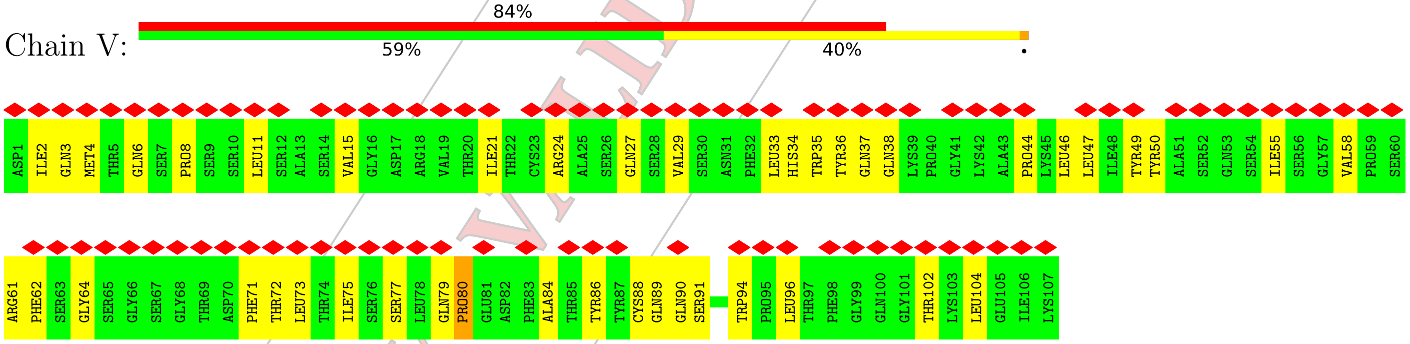

• Molecule 5: light chain of R33

Chain Q:

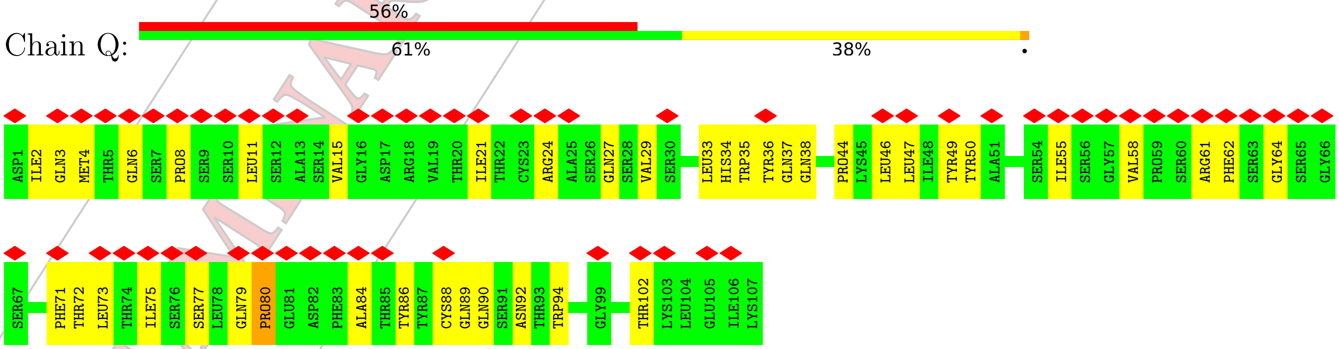

• Molecule 5: light chain of R33

Chain S:

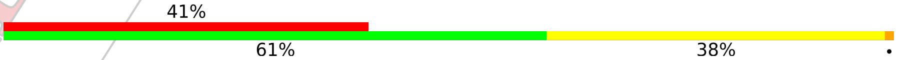

PRELIMINARY VALIDATION REPORT

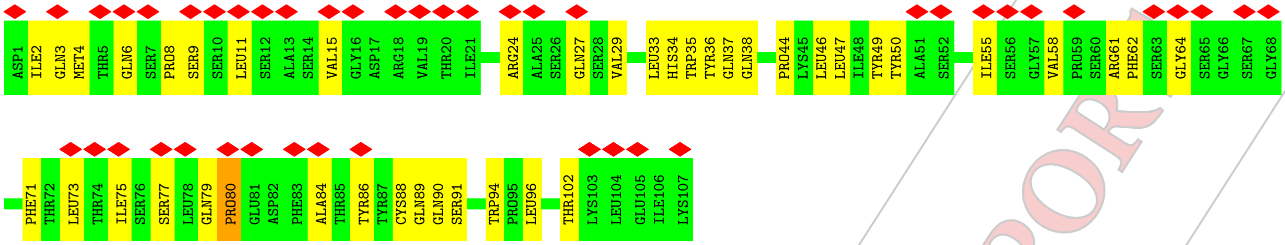

• Molecule 6: beta-D-mannopyranose-(1-4)-2-acetamido-2-deoxy-beta-D-glucopyranose-(1-4)-2-acetamido-2-deoxy-beta-D-glucopyranose

Chain D:

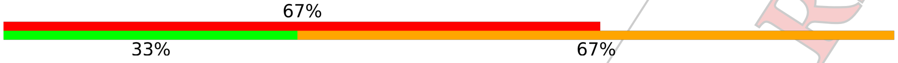

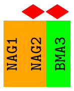

• Molecule 6: beta-D-mannopyranose-(1-4)-2-acetamido-2-deoxy-beta-D-glucopyranose-(1-4)-2-acetamido-2-deoxy-beta-D-glucopyranose

Chain E:

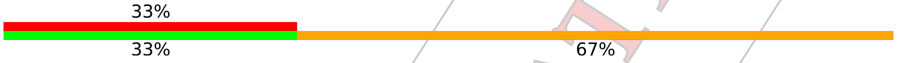

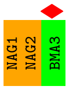

• Molecule 6: beta-D-mannopyranose-(1-4)-2-acetamido-2-deoxy-beta-D-glucopyranose-(1-4)-2-acetamido-2-deoxy-beta-D-glucopyranose

Chain F:

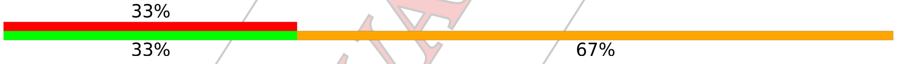

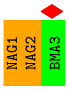

• Molecule 7: 2-acetamido-2-deoxy-beta-D-glucopyranose-(1-4)-2-acetamido-2-deoxy-beta-D-glucopyranose

Chain G:

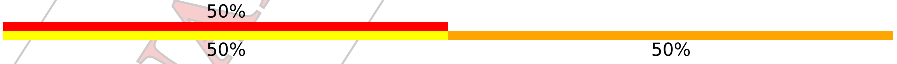

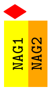

• Molecule 7: 2-acetamido-2-deoxy-beta-D-glucopyranose-(1-4)-2-acetamido-2-deoxy-beta-D-glucopyranose

Chain H:

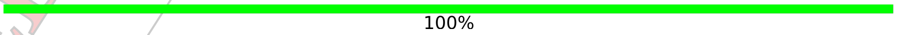

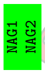

PRELIMINARY VALIDATION REPORT

• Molecule 7: 2-acetamido-2-deoxy-beta-D-glucopyranose-(1-4)-2-acetamido-2-deoxy-beta-D-glucopyranose

Chain I:

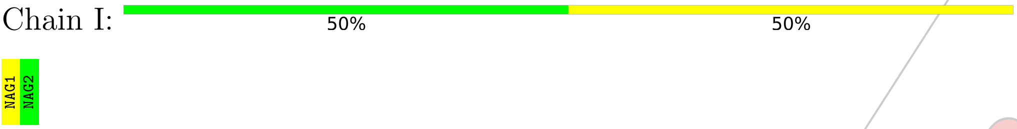

• Molecule 7: 2-acetamido-2-deoxy-beta-D-glucopyranose-(1-4)-2-acetamido-2-deoxy-beta-D-glucopyranose

Chain J:

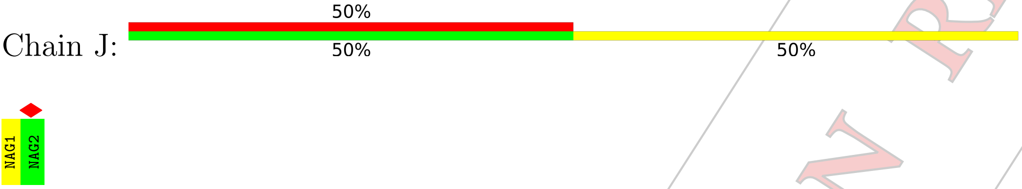

• Molecule 7: 2-acetamido-2-deoxy-beta-D-glucopyranose-(1-4)-2-acetamido-2-deoxy-beta-D-glucopyranose

Chain K:

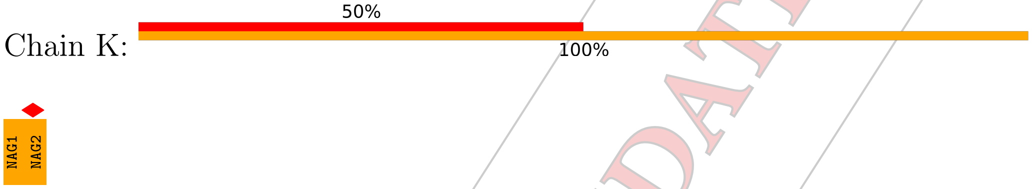

• Molecule 7: 2-acetamido-2-deoxy-beta-D-glucopyranose-(1-4)-2-acetamido-2-deoxy-beta-D-glucopyranose

Chain L:

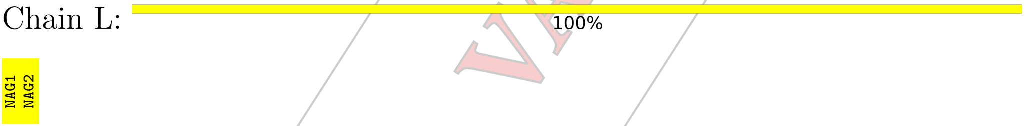

• Molecule 7: 2-acetamido-2-deoxy-beta-D-glucopyranose-(1-4)-2-acetamido-2-deoxy-beta-D-glucopyranose

Chain M:

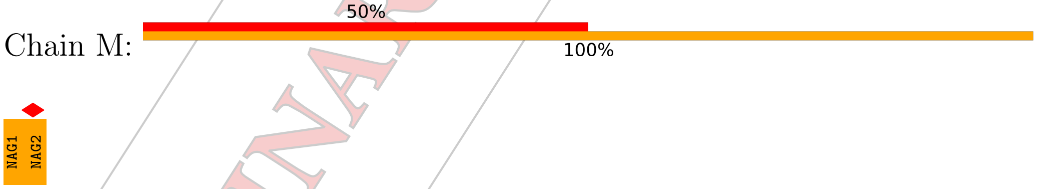

• Molecule 7: 2-acetamido-2-deoxy-beta-D-glucopyranose-(1-4)-2-acetamido-2-deoxy-beta-D-glucopyranose

Chain N:

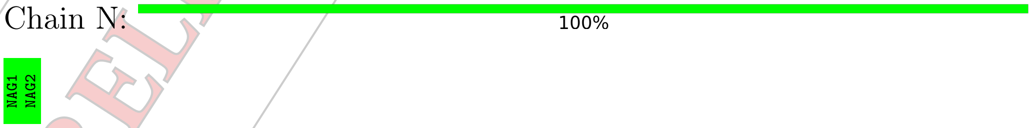

PRELIMINARY VALIDATION REPORT

• Molecule 7: 2-acetamido-2-deoxy-beta-D-glucopyranose-(1-4)-2-acetamido-2-deoxy-beta-D-gluc opyranose

Chain R:
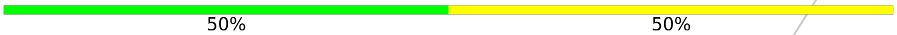

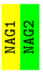

• Molecule 7: 2-acetamido-2-deoxy-beta-D-glucopyranose-(1-4)-2-acetamido-2-deoxy-beta-D-gluc opyranose

Chain T:
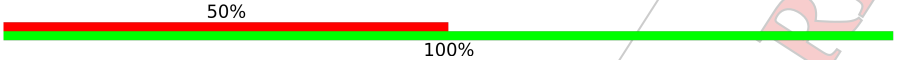

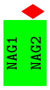

PRELIMINARY VALIDATION REPORT

4 Experimental information (i)

Property	Value	Source
EM reconstruction method	SINGLE PARTICLE	Depositor
Imposed symmetry	POINT, Not provided	
Number of particles used	Not provided	
Resolution determination method	Not provided	
CTF correction method	Not provided	
Microscope	Not provided	
Voltage (kV)	Not provided	
Electron dose ($e^-/\text{Å}^2$)	Not provided	
Minimum defocus (nm)	Not provided	
Maximum defocus (nm)	Not provided	
Magnification	Not provided	
Image detector	Not provided	
Maximum map value	1.657	Depositor
Minimum map value	-0.780	Depositor
Average map value	-0.000	Depositor
Map value standard deviation	0.036	Depositor
Recommended contour level	0.177	Depositor
Map size (Å)	432.00003, 432.00003, 432.00003	wwPDB
Map dimensions	400, 400, 400	wwPDB
Map angles (°)	90.0, 90.0, 90.0	wwPDB
Pixel spacing (Å)	1.08, 1.08, 1.08	Depositor

PRELIMINARY VALIDATION REPORT

5 Model quality (i)

5.1 Standard geometry (i)

Bond lengths and bond angles in the following residue types are not validated in this section: NAG, BMA

The Z score for a bond length (or angle) is the number of standard deviations the observed value is removed from the expected value. A bond length (or angle) with $|Z| > 5$ is considered an outlier worth inspection. RMSZ is the root-mean-square of all Z scores of the bond lengths (or angles).

Mol	Chain	Bond lengths		Bond angles	
		RMSZ	#$\|Z\|$ >5	RMSZ	#$\|Z\|$ >5
1	A	1.33	6/7936 (0.1%)	0.83	7/10811 (0.1%)
2	B	0.51	1/7907 (0.0%)	0.66	5/10768 (0.0%)
3	C	0.52	3/7851 (0.0%)	0.66	3/10703 (0.0%)
4	O	0.51	0/955	0.60	0/1300
4	P	0.51	0/955	0.60	0/1300
4	U	0.51	0/955	0.60	0/1300
5	Q	0.44	0/844	0.63	1/1146 (0.1%)
5	S	0.44	0/844	0.62	1/1146 (0.1%)
5	V	0.44	0/844	0.63	1/1146 (0.1%)
All	All	0.82	10/29091 (0.0%)	0.70	18/39620 (0.0%)

Chiral center outliers are detected by calculating the chiral volume of a chiral center and verifying if the center is modelled as a planar moiety or with the opposite hand.A planarity outlier is detected by checking planarity of atoms in a peptide group, atoms in a mainchain group or atoms of a sidechain that are expected to be planar.

Mol	Chain	#Chirality outliers	#Planarity outliers
1	A	1	2
2	B	0	2
4	O	0	1
4	P	0	1
4	U	0	1
5	Q	0	1
5	S	0	1
5	V	0	1
All	All	1	10

All (10) bond length outliers are listed below:

Mol	Chain	Res	Type	Atoms	Z	Observed(Å)	Ideal(Å)
1	A	516	GLU	CB-CG	87.00	3.17	1.52

Continued on next page...

PRELIMINARY VALIDATION REPORT

Continued from previous page...

Mol	Chain	Res	Type	Atoms	Z	Observed(Å)	Ideal(Å)
1	A	516	GLU	CA-CB	64.02	2.94	1.53
1	A	516	GLU	CG-CD	15.03	1.74	1.51
3	C	336	CYS	N-CA	-9.02	1.28	1.46
1	A	516	GLU	N-CA	8.44	1.63	1.46
3	C	336	CYS	CA-CB	-7.49	1.37	1.53
2	B	336	CYS	CA-CB	-7.15	1.38	1.53
3	C	336	CYS	CB-SG	6.85	1.93	1.82
1	A	336	CYS	CA-CB	-5.42	1.42	1.53
1	A	515	PHE	C-N	5.29	1.46	1.34

All (18) bond angle outliers are listed below:

Mol	Chain	Res	Type	Atoms	Z	Observed(^{o})	Ideal(^{o})
1	A	516	GLU	CA-CB-CG	-47.55	8.80	113.40
1	A	516	GLU	N-CA-CB	14.55	136.78	110.60
2	B	30	ASN	C-N-CA	9.23	144.79	121.70
5	V	80	PRO	CA-N-CD	-8.89	99.05	111.50
5	Q	80	PRO	CA-N-CD	-8.88	99.07	111.50
5	S	80	PRO	CA-N-CD	-8.88	99.08	111.50
3	C	703	ASN	CB-CA-C	-7.92	94.56	110.40
2	B	567	ARG	NE-CZ-NH2	-7.30	116.65	120.30
1	A	983	ARG	NE-CZ-NH2	-6.45	117.08	120.30
2	B	938	LEU	CA-CB-CG	6.03	129.18	115.30
1	A	515	PHE	C-N-CA	5.85	136.32	121.70
1	A	617	CYS	CA-CB-SG	5.67	124.20	114.00
3	C	1032	CYS	CA-CB-SG	5.55	123.99	114.00
1	A	436	TRP	CA-CB-CG	5.33	123.84	113.70
3	C	436	TRP	CA-CB-CG	5.33	123.83	113.70
2	B	436	TRP	CA-CB-CG	5.31	123.80	113.70
2	B	546	LEU	CA-CB-CG	5.15	127.15	115.30
1	A	336	CYS	CB-CA-C	5.05	120.51	110.40

All (1) chirality outliers are listed below:

Mol	Chain	Res	Type	Atom
1	A	516	GLU	CA

All (10) planarity outliers are listed below:

Mol	Chain	Res	Type	Group
1	A	513	LEU	Mainchain
1	A	983	ARG	Sidechain

Continued on next page...

PRELIMINARY VALIDATION REPORT

Continued from previous page...

Mol	Chain	Res	Type	Group
2	B	333	THR	Peptide
2	B	567	ARG	Sidechain
4	O	106	PHE	Peptide
4	P	106	PHE	Peptide
5	Q	29	VAL	Peptide
5	S	29	VAL	Peptide
4	U	106	PHE	Peptide
5	V	29	VAL	Peptide

5.2 Too-close contacts (i)

In the following table, the Non-H and H(model) columns list the number of non-hydrogen atoms and hydrogen atoms in the chain respectively. The H(added) column lists the number of hydrogen atoms added and optimized by MolProbity. The Clashes column lists the number of clashes within the asymmetric unit, whereas Symm-Clashes lists symmetry-related clashes.

Mol	Chain	Non-H	H(model)	H(added)	Clashes	Symm-Clashes
1	A	7761	0	7507	221	0
2	B	7732	0	7480	238	0
3	C	7677	0	7374	233	0
4	O	928	0	869	57	0
4	P	928	0	869	54	0
4	U	928	0	869	55	0
5	Q	824	0	801	39	0
5	S	824	0	801	39	0
5	V	824	0	801	42	0
6	D	39	0	34	1	0
6	E	39	0	34	1	0
6	F	39	0	34	1	0
7	G	28	0	25	1	0
7	H	28	0	25	0	0
7	I	28	0	25	0	0
7	J	28	0	25	0	0
7	K	28	0	25	2	0
7	L	28	0	25	1	0
7	M	28	0	25	2	0
7	N	28	0	25	0	0
7	R	28	0	25	0	0
7	T	28	0	25	0	0
8	A	98	0	91	3	0
8	B	154	0	143	1	0
8	C	112	0	104	2	0

Continued on next page...

PRELIMINARY VALIDATION REPORT

Continued from previous page...

Mol	Chain	Non-H	H(model)	H(added)	Clashes	Symm-Clashes
All	All	29187	0	28061	867	0

The all-atom clashscore is defined as the number of clashes found per 1000 atoms (including hydrogen atoms). The all-atom clashscore for this structure is 15.

All (867) close contacts within the same asymmetric unit are listed below, sorted by their clash magnitude.

Atom-1	Atom-2	Interatomic distance (Å)	Clash overlap (Å)
1:A:357:ARG:HH12	2:B:167:THR:CA	1.12	1.63
2:B:363:ALA:CB	2:B:524:VAL:HG22	1.27	1.55
3:C:363:ALA:HB2	3:C:524:VAL:CG1	1.30	1.53
1:A:395:VAL:HG23	1:A:524:VAL:CG1	1.39	1.53
1:A:516:GLU:CD	1:A:516:GLU:CG	1.74	1.53
3:C:363:ALA:CB	3:C:524:VAL:CG1	1.92	1.46
2:B:363:ALA:CB	2:B:524:VAL:CG2	1.85	1.41
2:B:391:CYS:SG	2:B:544:ASN:CA	2.11	1.39
2:B:363:ALA:HB2	2:B:524:VAL:CG2	0.89	1.35
2:B:391:CYS:SG	2:B:544:ASN:HB2	1.66	1.34
2:B:391:CYS:SG	2:B:544:ASN:CB	2.17	1.33
2:B:386:LYS:HD2	3:C:983:ARG:C	1.47	1.32
2:B:391:CYS:HB2	2:B:544:ASN:O	1.31	1.30
2:B:382:VAL:HG13	3:C:983:ARG:CD	1.62	1.29
1:A:357:ARG:NH1	2:B:167:THR:CA	1.90	1.28
2:B:383:SER:CB	3:C:984:LEU:HA	1.62	1.27
1:A:362:VAL:CG1	1:A:527:PRO:HA	1.66	1.25
1:A:357:ARG:NH1	2:B:167:THR:HA	1.44	1.23
2:B:357:ARG:NE	3:C:230:PRO:CG	1.95	1.23
2:B:393:THR:HB	2:B:520:ALA:O	1.40	1.22
1:A:394:ASN:ND2	1:A:516:GLU:CB	2.03	1.21
2:B:361:CYS:O	2:B:524:VAL:HA	1.38	1.19
3:C:391:CYS:HB2	3:C:544:ASN:O	1.37	1.19
1:A:357:ARG:HH12	2:B:167:THR:CB	1.55	1.19
1:A:394:ASN:H	1:A:516:GLU:CG	1.57	1.18
2:B:383:SER:HB3	3:C:983:ARG:O	1.02	1.17
2:B:391:CYS:SG	2:B:544:ASN:C	2.23	1.16
2:B:391:CYS:CB	2:B:544:ASN:O	1.92	1.16
2:B:357:ARG:NE	3:C:230:PRO:HG3	1.55	1.16
2:B:336:CYS:SG	2:B:524:VAL:HG23	1.85	1.15
1:A:362:VAL:CG1	1:A:527:PRO:CA	2.24	1.15
3:C:363:ALA:HB3	3:C:524:VAL:HG12	1.25	1.14
2:B:383:SER:CB	3:C:983:ARG:O	1.95	1.12

Continued on next page...

PRELIMINARY VALIDATION REPORT

Continued from previous page...

Atom-1	Atom-2	Interatomic distance (Å)	Clash overlap (Å)
1:A:393:THR:C	1:A:516:GLU:CB	2.18	1.12
1:A:395:VAL:HG23	1:A:524:VAL:HG13	1.29	1.11
2:B:391:CYS:SG	2:B:544:ASN:HA	1.90	1.11
2:B:361:CYS:N	2:B:523:THR:O	1.80	1.11
1:A:362:VAL:HG13	1:A:527:PRO:HA	1.35	1.08
2:B:383:SER:HB2	3:C:984:LEU:HD23	1.31	1.08
3:C:363:ALA:HB2	3:C:524:VAL:HG11	1.26	1.08
3:C:363:ALA:CB	3:C:524:VAL:HG12	1.80	1.08
2:B:382:VAL:CG1	3:C:983:ARG:HD2	1.85	1.06
2:B:383:SER:CB	3:C:984:LEU:HD23	1.83	1.06
2:B:334:ASN:HD22	2:B:361:CYS:HA	1.20	1.06
1:A:395:VAL:CG2	1:A:524:VAL:CG1	2.32	1.06
2:B:363:ALA:HB2	2:B:524:VAL:HG23	1.14	1.06
1:A:394:ASN:H	1:A:516:GLU:HA	1.03	1.05
2:B:357:ARG:HE	3:C:230:PRO:CG	1.60	1.05
1:A:395:VAL:HG23	1:A:524:VAL:HG11	1.08	1.05
1:A:394:ASN:H	1:A:516:GLU:HG2	1.13	1.05
2:B:363:ALA:HB2	2:B:524:VAL:HG21	1.38	1.05
2:B:383:SER:HB2	3:C:984:LEU:CA	1.86	1.04
2:B:383:SER:HB2	3:C:984:LEU:CD2	1.88	1.04
3:C:363:ALA:HB2	3:C:524:VAL:HG13	1.08	1.03
2:B:359:SER:CA	2:B:523:THR:HG21	1.86	1.03
2:B:363:ALA:N	2:B:525:CYS:O	1.92	1.03
2:B:382:VAL:HG13	3:C:983:ARG:HD2	1.04	1.02
1:A:395:VAL:CG2	1:A:524:VAL:HG11	1.90	1.01
1:A:393:THR:OG1	1:A:516:GLU:CB	2.08	1.01
2:B:357:ARG:HE	3:C:230:PRO:HG2	1.23	1.01
2:B:334:ASN:ND2	2:B:361:CYS:HA	1.74	1.01
2:B:363:ALA:O	2:B:526:GLY:HA2	1.59	1.00
2:B:382:VAL:HG12	3:C:983:ARG:HB2	1.44	1.00
1:A:395:VAL:HG21	1:A:524:VAL:CG2	1.91	1.00
2:B:363:ALA:HB3	2:B:524:VAL:HG22	1.41	1.00
3:C:393:THR:CG2	3:C:520:ALA:O	2.10	1.00
2:B:336:CYS:SG	2:B:524:VAL:CG2	2.50	0.99
2:B:359:SER:HA	2:B:523:THR:CG2	1.91	0.99
1:A:394:ASN:HD22	1:A:516:GLU:CB	1.66	0.99
3:C:391:CYS:SG	3:C:544:ASN:HA	2.03	0.98
4:U:12:LYS:HG3	4:U:18:VAL:HG11	1.45	0.98
4:O:12:LYS:HG3	4:O:18:VAL:HG11	1.45	0.98
1:A:393:THR:HA	1:A:522:ALA:HA	1.43	0.98
1:A:394:ASN:N	1:A:516:GLU:HA	1.78	0.98

Continued on next page...

PRELIMINARY VALIDATION REPORT

Continued from previous page...

Atom-1	Atom-2	Interatomic distance (Å)	Clash overlap (Å)
1:A:393:THR:OG1	1:A:516:GLU:CA	2.11	0.98
4:U:13:LYS:HD3	4:U:14:PRO:HD2	1.46	0.98
2:B:391:CYS:SG	2:B:544:ASN:O	2.22	0.97
4:P:12:LYS:HG3	4:P:18:VAL:HG11	1.45	0.97
4:O:13:LYS:HD3	4:O:14:PRO:HD2	1.46	0.96
2:B:359:SER:CA	2:B:523:THR:CG2	2.42	0.96
4:P:13:LYS:HD3	4:P:14:PRO:HD2	1.46	0.96
3:C:360:ASN:H	3:C:523:THR:HB	1.30	0.95
1:A:362:VAL:HG11	1:A:527:PRO:HA	1.49	0.95
1:A:394:ASN:H	1:A:516:GLU:CA	1.75	0.94
2:B:396:TYR:HE2	3:C:200:TYR:HH	0.96	0.94
2:B:393:THR:CB	2:B:520:ALA:O	2.15	0.94
1:A:357:ARG:NH1	2:B:167:THR:O	2.02	0.93
1:A:393:THR:CB	1:A:516:GLU:CB	2.46	0.93
1:A:393:THR:OG1	1:A:516:GLU:CG	2.16	0.92
2:B:382:VAL:CG1	3:C:983:ARG:CD	2.46	0.92
2:B:382:VAL:C	3:C:984:LEU:HD21	1.90	0.92
3:C:391:CYS:SG	3:C:544:ASN:CA	2.58	0.92
1:A:357:ARG:NH1	2:B:167:THR:C	2.22	0.92
2:B:383:SER:HB2	3:C:984:LEU:HA	0.93	0.92
3:C:393:THR:HA	3:C:522:ALA:HA	1.49	0.92
1:A:391:CYS:HA	1:A:525:CYS:SG	2.09	0.92
1:A:364:ASP:OD1	1:A:527:PRO:HD2	1.69	0.92
2:B:357:ARG:NE	3:C:230:PRO:HG2	1.81	0.92
1:A:394:ASN:CG	1:A:516:GLU:CA	2.39	0.91
3:C:364:ASP:OD2	3:C:527:PRO:HD3	1.71	0.91
3:C:393:THR:HG21	3:C:520:ALA:O	1.71	0.90
2:B:396:TYR:HE2	3:C:200:TYR:OH	1.53	0.90
1:A:883:THR:HG23	3:C:707:TYR:HB2	1.54	0.89
1:A:394:ASN:N	1:A:516:GLU:CG	2.30	0.89
2:B:396:TYR:CE2	3:C:200:TYR:OH	2.23	0.89
1:A:395:VAL:HG21	1:A:524:VAL:HG22	1.55	0.89
3:C:650:LEU:HD21	3:C:653:ALA:HB3	1.55	0.89
2:B:355:ARG:NH2	3:C:230:PRO:O	2.04	0.89
1:A:394:ASN:OD1	1:A:516:GLU:CA	2.22	0.88
2:B:396:TYR:OH	3:C:200:TYR:HE1	1.55	0.88
1:A:357:ARG:CZ	2:B:167:THR:HA	2.03	0.87
1:A:395:VAL:CG2	1:A:524:VAL:CG2	2.51	0.87
3:C:360:ASN:H	3:C:523:THR:CB	1.87	0.87
2:B:396:TYR:OH	3:C:200:TYR:CE1	2.27	0.86
4:U:4:LEU:HD13	4:U:109:TRP:HE1	1.40	0.86

Continued on next page...

PRELIMINARY VALIDATION REPORT

Continued from previous page...

Atom-1	Atom-2	Interatomic distance (Å)	Clash overlap (Å)
1:A:200:TYR:CE2	3:C:396:TYR:OH	2.28	0.86
3:C:193:VAL:HG23	3:C:223:LEU:HD12	1.56	0.86
2:B:386:LYS:CD	3:C:983:ARG:C	2.42	0.85
1:A:357:ARG:HH12	2:B:167:THR:C	1.79	0.85
4:O:4:LEU:HD13	4:O:109:TRP:HE1	1.40	0.85
2:B:382:VAL:HG13	3:C:983:ARG:HD3	1.58	0.84
3:C:391:CYS:CB	3:C:544:ASN:O	2.22	0.84
4:P:4:LEU:HD13	4:P:109:TRP:HE1	1.40	0.84
1:A:394:ASN:N	1:A:516:GLU:CA	2.37	0.84
2:B:334:ASN:HD22	2:B:361:CYS:CA	1.91	0.83
2:B:361:CYS:O	2:B:524:VAL:CA	2.26	0.83
1:A:357:ARG:NH1	2:B:167:THR:CB	2.31	0.83
3:C:360:ASN:N	3:C:523:THR:HB	1.93	0.82
1:A:362:VAL:HG11	1:A:527:PRO:CA	2.06	0.81
3:C:363:ALA:CB	3:C:524:VAL:HG13	1.82	0.81
3:C:393:THR:HB	3:C:520:ALA:O	1.80	0.80
2:B:336:CYS:SG	2:B:524:VAL:CB	2.70	0.80
1:A:395:VAL:CG2	1:A:524:VAL:HG21	2.12	0.80
2:B:383:SER:HB3	3:C:983:ARG:C	2.02	0.80
2:B:383:SER:CA	3:C:984:LEU:HD23	2.12	0.80
1:A:395:VAL:HG21	1:A:524:VAL:HG21	1.64	0.79
1:A:393:THR:H	1:A:516:GLU:CD	1.85	0.79
1:A:357:ARG:NH1	2:B:167:THR:OG1	2.16	0.79
1:A:364:ASP:OD1	1:A:527:PRO:CD	2.31	0.79
2:B:363:ALA:H	2:B:525:CYS:N	1.80	0.79
2:B:383:SER:N	3:C:984:LEU:HD23	1.98	0.78
3:C:393:THR:CB	3:C:520:ALA:O	2.30	0.78
2:B:383:SER:N	3:C:984:LEU:CD2	2.46	0.78
2:B:355:ARG:HH21	3:C:230:PRO:HB2	1.47	0.78
2:B:391:CYS:CB	2:B:544:ASN:HB2	2.13	0.78
3:C:391:CYS:HB3	3:C:522:ALA:CB	2.14	0.77
2:B:359:SER:HA	2:B:523:THR:HG23	1.65	0.77
2:B:359:SER:CB	2:B:523:THR:HG21	2.14	0.77
2:B:361:CYS:O	2:B:523:THR:O	2.03	0.77
1:A:394:ASN:N	1:A:516:GLU:CB	2.48	0.76
3:C:388:ASN:O	3:C:526:GLY:HA3	1.85	0.76
2:B:336:CYS:SG	2:B:524:VAL:HB	2.25	0.76
3:C:391:CYS:SG	3:C:544:ASN:C	2.64	0.76
2:B:363:ALA:O	2:B:526:GLY:CA	2.33	0.76
2:B:386:LYS:HD2	3:C:983:ARG:O	1.84	0.76
2:B:383:SER:CB	3:C:984:LEU:CD2	2.57	0.75

Continued on next page...

PRELIMINARY VALIDATION REPORT

Continued from previous page...

Atom-1	Atom-2	Interatomic distance (Å)	Clash overlap (Å)
2:B:383:SER:CA	3:C:984:LEU:CD2	2.65	0.75
1:A:362:VAL:CG1	1:A:527:PRO:CB	2.64	0.75
3:C:108:THR:OG1	3:C:234:ASN:O	2.03	0.75
2:B:383:SER:CB	3:C:984:LEU:CA	2.56	0.74
1:A:362:VAL:HG11	1:A:527:PRO:CB	2.16	0.74
4:U:51:ILE:HD12	4:U:72:LEU:HB2	1.70	0.74
3:C:391:CYS:HB3	3:C:522:ALA:HB1	1.70	0.73
1:A:516:GLU:CD	1:A:516:GLU:N	2.42	0.73
1:A:983:ARG:O	3:C:383:SER:HB3	1.88	0.73
2:B:386:LYS:HD2	3:C:983:ARG:CA	2.19	0.73
3:C:361:CYS:O	3:C:524:VAL:HA	1.89	0.72
2:B:386:LYS:HD2	3:C:984:LEU:N	2.01	0.72
3:C:1106:GLN:HE21	3:C:1109:PHE:HB3	1.54	0.72
3:C:363:ALA:CB	3:C:524:VAL:HG11	1.91	0.72
4:O:51:ILE:HD12	4:O:72:LEU:HB2	1.70	0.72
2:B:701:VAL:HG13	3:C:787:GLN:HG2	1.70	0.72
2:B:393:THR:CG2	2:B:520:ALA:O	2.36	0.72
2:B:393:THR:HA	2:B:522:ALA:HA	1.71	0.72
3:C:335:LEU:HG	3:C:362:VAL:O	1.89	0.72
1:A:391:CYS:CA	1:A:525:CYS:SG	2.79	0.71
4:P:51:ILE:HD12	4:P:72:LEU:HB2	1.70	0.71
1:A:394:ASN:O	1:A:516:GLU:N	2.23	0.71
2:B:395:VAL:HG21	2:B:524:VAL:HG11	1.73	0.71
1:A:364:ASP:CG	1:A:527:PRO:CG	2.59	0.70
1:A:394:ASN:N	1:A:516:GLU:HG2	1.96	0.70
1:A:362:VAL:HG11	1:A:527:PRO:HB3	1.73	0.70
2:B:364:ASP:OD2	2:B:527:PRO:HG3	1.92	0.69
1:A:362:VAL:CG1	1:A:527:PRO:HB3	2.21	0.69
4:P:4:LEU:HB2	4:P:109:TRP:CD1	2.27	0.69
1:A:395:VAL:N	1:A:524:VAL:HG11	2.07	0.69
8:A:1302:NAG:H83	3:C:558:LYS:HZ1	1.57	0.69
2:B:656:VAL:HG11	2:B:693:ILE:HD12	1.74	0.69
4:U:4:LEU:HB2	4:U:109:TRP:CD1	2.27	0.69
1:A:362:VAL:HG13	1:A:527:PRO:CA	2.07	0.69
2:B:363:ALA:HB2	2:B:524:VAL:HG22	0.71	0.69
1:A:395:VAL:CG2	1:A:524:VAL:HG13	2.12	0.68
2:B:362:VAL:C	2:B:525:CYS:O	2.32	0.68
4:O:4:LEU:HB2	4:O:109:TRP:CD1	2.27	0.68
1:A:787:GLN:HB3	3:C:701:VAL:HG13	1.75	0.68
4:U:13:LYS:HD3	4:U:14:PRO:CD	2.23	0.68
1:A:46:SER:HA	1:A:279:TYR:O	1.94	0.68

Continued on next page...

PRELIMINARY VALIDATION REPORT

Continued from previous page...

Atom-1	Atom-2	Interatomic distance (Å)	Clash overlap (Å)
2:B:382:VAL:C	3:C:984:LEU:CD2	2.63	0.68
2:B:355:ARG:NH2	3:C:230:PRO:HB2	2.08	0.67
2:B:905:ARG:NH1	2:B:1049:LEU:O	2.27	0.67
4:P:72:LEU:HD21	4:P:74:THR:HB	1.77	0.67
2:B:37:TYR:OH	2:B:53:ASP:OD2	2.11	0.67
1:A:722:VAL:HG12	1:A:1065:VAL:HG22	1.77	0.66
4:U:83:ILE:HG23	4:U:86:LEU:HD11	1.77	0.66
4:U:72:LEU:HD21	4:U:74:THR:HB	1.77	0.66
1:A:363:ALA:HB3	1:A:525:CYS:C	2.15	0.66
3:C:656:VAL:HG11	3:C:693:ILE:HD12	1.77	0.66
2:B:359:SER:C	2:B:523:THR:HG21	1.87	0.66
3:C:360:ASN:CA	3:C:523:THR:HB	2.24	0.66
4:O:83:ILE:HG23	4:O:86:LEU:HD11	1.78	0.66
4:P:13:LYS:HD3	4:P:14:PRO:CD	2.23	0.66
1:A:364:ASP:CA	1:A:527:PRO:HD3	2.25	0.66
4:P:83:ILE:HG23	4:P:86:LEU:HD11	1.78	0.66
3:C:516:GLU:HG2	3:C:519:HIS:H	1.60	0.66
2:B:176:LEU:HD23	2:B:190:ARG:HD2	1.78	0.65
1:A:394:ASN:CG	1:A:516:GLU:CB	2.65	0.65
2:B:404:GLY:HA2	2:B:508:TYR:HD2	1.62	0.65
3:C:364:ASP:OD2	3:C:527:PRO:CD	2.43	0.65
3:C:404:GLY:HA2	3:C:508:TYR:HD1	1.62	0.65
4:O:13:LYS:HD3	4:O:14:PRO:CD	2.23	0.65
1:A:404:GLY:HA2	1:A:508:TYR:HD2	1.62	0.65
1:A:725:GLU:OE1	1:A:1028:LYS:NZ	2.28	0.65
3:C:35:GLY:HA3	3:C:56:LEU:HB3	1.77	0.65
4:O:72:LEU:HD21	4:O:74:THR:HB	1.77	0.65
2:B:773:GLU:OE2	2:B:1019:ARG:NH1	2.30	0.64
5:V:8:PRO:HG2	5:V:11:LEU:HB3	1.80	0.64
3:C:905:ARG:NH1	3:C:1049:LEU:O	2.28	0.64
1:A:360:ASN:N	1:A:523:THR:OG1	2.30	0.64
3:C:91:TYR:OH	3:C:191:GLU:OE2	2.15	0.63
2:B:330:PRO:C	2:B:332:ILE:H	2.00	0.63
3:C:360:ASN:HA	3:C:523:THR:HB	1.80	0.63
5:Q:8:PRO:HG2	5:Q:11:LEU:HB3	1.80	0.63
2:B:382:VAL:CG1	3:C:983:ARG:HB2	2.25	0.63
2:B:395:VAL:CG2	2:B:524:VAL:HG11	2.28	0.63
1:A:393:THR:O	1:A:516:GLU:CB	2.47	0.63
1:A:567:ARG:HD3	1:A:571:ASP:HA	1.80	0.62
1:A:364:ASP:HA	1:A:527:PRO:HD2	1.82	0.62
5:S:8:PRO:HG2	5:S:11:LEU:HB3	1.80	0.62

Continued on next page...

PRELIMINARY VALIDATION REPORT

Continued from previous page...

Atom-1	Atom-2	Interatomic distance (Å)	Clash overlap (Å)
1:A:364:ASP:CG	1:A:527:PRO:HG3	2.20	0.62
4:P:106:PHE:O	4:P:107:ASP:HB2	2.00	0.62
2:B:393:THR:HB	2:B:520:ALA:C	2.16	0.62
2:B:743:CYS:SG	2:B:750:SER:N	2.73	0.62
5:Q:15:VAL:HG13	5:Q:80:PRO:HD2	1.82	0.62
1:A:983:ARG:O	3:C:383:SER:CB	2.48	0.61
2:B:363:ALA:CA	2:B:524:VAL:HG23	2.29	0.61
1:A:64:TRP:HE1	1:A:264:ALA:HB1	1.65	0.61
1:A:82:PRO:HG2	1:A:84:LEU:HD21	1.82	0.61
1:A:362:VAL:HG12	1:A:527:PRO:CA	2.24	0.61
5:S:15:VAL:HG13	5:S:80:PRO:HD2	1.82	0.61
5:V:15:VAL:HG13	5:V:80:PRO:HD2	1.82	0.61
2:B:425:LEU:HD12	2:B:426:PRO:HD2	1.83	0.61
4:U:106:PHE:O	4:U:107:ASP:HB2	2.00	0.61
1:A:393:THR:CA	1:A:516:GLU:CB	2.78	0.61
1:A:425:LEU:HD12	1:A:426:PRO:HD2	1.83	0.61
3:C:323:THR:OG1	3:C:324:GLU:OE2	2.19	0.61
5:Q:36:TYR:HD1	5:Q:46:LEU:HA	1.66	0.61
1:A:360:ASN:CA	1:A:523:THR:OG1	2.49	0.61
1:A:364:ASP:HA	1:A:527:PRO:CD	2.31	0.60
4:O:106:PHE:O	4:O:107:ASP:HB2	2.00	0.60
1:A:984:LEU:HD21	3:C:381:GLY:O	2.01	0.60
4:U:27:TYR:CZ	4:U:98:ARG:HD3	2.37	0.60
5:V:36:TYR:HD1	5:V:46:LEU:HA	1.66	0.60
1:A:905:ARG:NH1	1:A:1049:LEU:O	2.34	0.60
1:A:984:LEU:HA	3:C:383:SER:HB2	1.84	0.60
3:C:546:LEU:HD21	3:C:573:THR:HG21	1.81	0.60
3:C:393:THR:HB	3:C:520:ALA:C	2.21	0.60
1:A:393:THR:HB	1:A:516:GLU:CB	2.32	0.60
1:A:701:VAL:HG13	2:B:787:GLN:HG2	1.82	0.60
1:A:364:ASP:OD1	1:A:527:PRO:CG	2.49	0.60
1:A:364:ASP:CG	1:A:527:PRO:CD	2.70	0.60
1:A:364:ASP:CB	1:A:527:PRO:HD3	2.31	0.60
1:A:393:THR:C	1:A:516:GLU:HB3	2.20	0.60
1:A:401:VAL:HG22	1:A:509:ARG:HG2	1.84	0.60
2:B:401:VAL:HG22	2:B:509:ARG:HG2	1.84	0.60
1:A:983:ARG:HB3	3:C:390:LEU:HD21	1.82	0.59
3:C:425:LEU:HD12	3:C:426:PRO:HD2	1.83	0.59
5:V:33:LEU:HD13	5:V:71:PHE:CD2	2.37	0.59
5:Q:33:LEU:HD13	5:Q:71:PHE:CD2	2.37	0.59
4:P:27:TYR:CZ	4:P:98:ARG:HD3	2.37	0.59

Continued on next page...

PRELIMINARY VALIDATION REPORT

Continued from previous page...

Atom-1	Atom-2	Interatomic distance (Å)	Clash overlap (Å)
1:A:362:VAL:CG1	1:A:527:PRO:N	2.65	0.59
2:B:391:CYS:HB3	2:B:522:ALA:CB	2.32	0.59
5:S:33:LEU:HD13	5:S:71:PHE:CD1	2.37	0.59
5:S:36:TYR:HD2	5:S:46:LEU:HA	1.66	0.59
2:B:355:ARG:HH22	3:C:230:PRO:C	2.05	0.59
3:C:811:LYS:HG3	3:C:812:PRO:HD2	1.85	0.59
4:O:27:TYR:CZ	4:O:98:ARG:HD3	2.37	0.59
1:A:395:VAL:H	1:A:524:VAL:HG11	1.66	0.59
1:A:789:TYR:HA	3:C:703:ASN:O	2.02	0.59
3:C:985:ASP:OD1	3:C:985:ASP:N	2.30	0.59
2:B:359:SER:HB3	2:B:523:THR:HG21	1.84	0.59
3:C:821:LEU:O	3:C:825:LYS:HG2	2.03	0.59
3:C:280:ASN:HD21	8:C:1301:NAG:H82	1.68	0.58
3:C:743:CYS:SG	3:C:750:SER:N	2.76	0.58
2:B:383:SER:HB2	3:C:984:LEU:HD22	1.82	0.58
2:B:390:LEU:HD21	3:C:983:ARG:HB3	1.86	0.58
1:A:393:THR:O	1:A:516:GLU:HB3	2.03	0.58
3:C:401:VAL:HG22	3:C:509:ARG:HG2	1.84	0.58
4:U:97:ALA:HB2	4:U:108:VAL:HG23	1.86	0.58
4:O:105:TYR:HB3	5:Q:34:HIS:CE1	2.39	0.58
7:K:1:NAG:H83	7:K:1:NAG:H3	1.85	0.58
3:C:273:ARG:HD3	3:C:292:ALA:HB3	1.86	0.58
3:C:299:THR:HG22	3:C:315:THR:HG21	1.84	0.58
4:O:97:ALA:HB2	4:O:108:VAL:HG23	1.86	0.58
1:A:707:TYR:HB2	2:B:883:THR:HG23	1.86	0.58
4:P:105:TYR:HB3	5:S:34:HIS:CE1	2.39	0.58
1:A:555:SER:HB3	1:A:586:ASP:HB2	1.85	0.58
2:B:362:VAL:CG1	2:B:525:CYS:O	2.52	0.58
1:A:703:ASN:O	2:B:789:TYR:HA	2.04	0.58
3:C:36:VAL:HG11	3:C:220:PHE:CZ	2.39	0.58
5:V:6:GLN:NE2	5:V:88:CYS:SG	2.77	0.58
4:O:51:ILE:HG21	4:O:70:PHE:HB2	1.86	0.58
4:P:58:PRO:HB2	4:P:60:TYR:CZ	2.39	0.58
1:A:763:LEU:HD22	1:A:1008:VAL:HG21	1.86	0.57
5:Q:46:LEU:HD21	5:Q:49:TYR:HB3	1.86	0.57
5:S:46:LEU:HD21	5:S:49:TYR:HB3	1.86	0.57
1:A:883:THR:HG23	3:C:707:TYR:CB	2.32	0.57
2:B:746:SER:HB2	2:B:749:CYS:SG	2.44	0.57
4:P:51:ILE:HG21	4:P:70:PHE:HB2	1.86	0.57
2:B:722:VAL:HG22	2:B:930:ALA:HB1	1.86	0.57
1:A:393:THR:O	1:A:523:THR:HG22	2.03	0.57

Continued on next page...

PRELIMINARY VALIDATION REPORT

Continued from previous page...

Atom-1	Atom-2	Interatomic distance (Å)	Clash overlap (Å)
4:U:34:MET:HG2	4:U:79:ALA:HB1	1.86	0.57
4:O:58:PRO:HB2	4:O:60:TYR:CZ	2.39	0.57
5:S:6:GLN:NE2	5:S:88:CYS:SG	2.77	0.57
1:A:364:ASP:CB	1:A:527:PRO:CD	2.82	0.57
4:U:105:TYR:HB3	5:V:34:HIS:CE1	2.39	0.57
5:Q:6:GLN:NE2	5:Q:88:CYS:SG	2.77	0.57
7:K:2:NAG:H3	7:K:2:NAG:H83	1.87	0.57
5:V:46:LEU:HD21	5:V:49:TYR:HB3	1.86	0.57
1:A:702:GLU:HA	2:B:788:ILE:HB	1.87	0.57
2:B:363:ALA:CB	2:B:524:VAL:HG23	1.93	0.57
2:B:1106:GLN:HE21	2:B:1109:PHE:HB3	1.70	0.57
4:U:51:ILE:HG21	4:U:70:PHE:HB2	1.86	0.57
4:U:58:PRO:HB2	4:U:60:TYR:CZ	2.39	0.57
1:A:91:TYR:OH	1:A:191:GLU:OE1	2.17	0.56
2:B:294:ASP:OD2	2:B:294:ASP:N	2.38	0.56
1:A:394:ASN:H	1:A:516:GLU:H	1.51	0.56
4:O:34:MET:HG2	4:O:79:ALA:HB1	1.86	0.56
4:P:97:ALA:HB2	4:P:108:VAL:HG23	1.86	0.56
2:B:382:VAL:HG12	3:C:983:ARG:CB	2.29	0.56
2:B:170:TYR:CE1	2:B:172:SER:HB2	2.41	0.56
2:B:382:VAL:HA	3:C:983:ARG:HD2	1.87	0.56
2:B:386:LYS:HD3	3:C:982:SER:C	2.26	0.56
4:P:34:MET:HG2	4:P:79:ALA:HB1	1.86	0.56
8:A:1306:NAG:H3	8:A:1306:NAG:H83	1.87	0.56
2:B:386:LYS:CD	3:C:983:ARG:CA	2.83	0.56
5:S:34:HIS:HE1	5:S:50:TYR:CD2	2.24	0.56
1:A:362:VAL:HG12	1:A:527:PRO:N	2.21	0.56
4:O:12:LYS:HG3	4:O:18:VAL:CG1	2.30	0.56
5:Q:33:LEU:HA	5:Q:89:GLN:O	2.06	0.56
2:B:330:PRO:C	2:B:332:ILE:N	2.59	0.55
5:S:38:GLN:HG3	5:S:44:PRO:HD3	1.88	0.55
3:C:472:ILE:HD11	3:C:488:CYS:HB3	1.88	0.55
3:C:738:CYS:SG	3:C:739:THR:N	2.80	0.55
5:V:38:GLN:HG3	5:V:44:PRO:HD3	1.88	0.55
5:S:33:LEU:HA	5:S:89:GLN:O	2.06	0.55
1:A:379:CYS:HA	1:A:432:CYS:HA	1.89	0.55
1:A:395:VAL:CB	1:A:524:VAL:HG11	2.37	0.55
1:A:1141:LEU:HG	1:A:1145:LEU:HD13	1.88	0.55
2:B:391:CYS:HB3	2:B:522:ALA:HB3	1.89	0.55
5:Q:64:GLY:HA2	5:Q:73:LEU:HA	1.89	0.55
3:C:822:LEU:HD21	3:C:938:LEU:HD13	1.89	0.55

Continued on next page...

PRELIMINARY VALIDATION REPORT

Continued from previous page...

Atom-1	Atom-2	Interatomic distance (Å)	Clash overlap (Å)
5:V:64:GLY:HA2	5:V:73:LEU:HA	1.89	0.55
1:A:560:LEU:H	1:A:563:GLN:NE2	2.05	0.55
2:B:359:SER:CA	2:B:523:THR:HG23	2.25	0.55
3:C:379:CYS:HA	3:C:432:CYS:HA	1.89	0.55
5:V:33:LEU:HA	5:V:89:GLN:O	2.06	0.55
5:V:34:HIS:HE1	5:V:50:TYR:CD1	2.24	0.55
1:A:516:GLU:CD	1:A:516:GLU:C	2.65	0.55
2:B:170:TYR:HE1	2:B:172:SER:HB2	1.72	0.55
2:B:821:LEU:HD21	2:B:939:SER:HB2	1.87	0.55
4:O:107:ASP:HB3	4:O:109:TRP:CZ3	2.42	0.55
5:Q:38:GLN:HG3	5:Q:44:PRO:HD3	1.88	0.55
4:P:107:ASP:HB3	4:P:109:TRP:CZ3	2.42	0.55
3:C:364:ASP:OD2	3:C:527:PRO:HG3	2.06	0.55
4:O:98:ARG:NH2	4:O:109:TRP:HH2	2.05	0.55
2:B:706:ALA:HB1	7:M:2:NAG:O7	2.07	0.54
4:P:12:LYS:HG3	4:P:18:VAL:CG1	2.30	0.54
3:C:391:CYS:O	3:C:522:ALA:HB2	2.06	0.54
4:P:98:ARG:NH2	4:P:109:TRP:HH2	2.05	0.54
1:A:364:ASP:CA	1:A:527:PRO:CD	2.86	0.54
4:U:98:ARG:NH2	4:U:109:TRP:HH2	2.05	0.54
5:Q:34:HIS:HE1	5:Q:50:TYR:CD1	2.24	0.54
5:S:6:GLN:HE22	5:S:88:CYS:H	1.56	0.54
2:B:472:ILE:HD11	2:B:488:CYS:HB3	1.88	0.54
4:U:33:GLY:H	4:U:100:GLY:HA2	1.73	0.54
4:O:33:GLY:H	4:O:100:GLY:HA2	1.73	0.54
2:B:390:LEU:HD11	3:C:983:ARG:HB3	1.89	0.54
1:A:393:THR:CA	1:A:522:ALA:HA	2.30	0.54
4:P:33:GLY:H	4:P:100:GLY:HA2	1.73	0.54
4:U:107:ASP:HB3	4:U:109:TRP:CZ3	2.42	0.54
1:A:200:TYR:HE2	3:C:396:TYR:OH	1.88	0.53
2:B:604:THR:HG21	2:B:687:VAL:HG11	1.89	0.53
2:B:707:TYR:HB2	3:C:883:THR:HG23	1.90	0.53
3:C:662:CYS:HB2	3:C:697:MET:HG2	1.90	0.53
2:B:127:VAL:HG21	8:B:1301:NAG:H5	1.90	0.53
2:B:1039:ARG:NE	3:C:1031:GLU:OE1	2.41	0.53
3:C:52:GLN:OE1	3:C:52:GLN:N	2.39	0.53
2:B:134:GLN:HG2	2:B:162:SER:HB3	1.90	0.53
2:B:276:LEU:O	2:B:288:ALA:HA	2.08	0.53
2:B:379:CYS:HA	2:B:432:CYS:HA	1.89	0.53
5:S:64:GLY:HA2	5:S:73:LEU:HA	1.89	0.53
5:Q:34:HIS:HE1	5:Q:50:TYR:CE1	2.27	0.53

Continued on next page...

PRELIMINARY VALIDATION REPORT

Continued from previous page...

Atom-1	Atom-2	Interatomic distance (Å)	Clash overlap (Å)
3:C:472:ILE:HD13	3:C:484:LYS:HG3	1.91	0.53
1:A:105:ILE:HD12	1:A:239:GLN:HE21	1.73	0.53
1:A:121:ASN:HD21	1:A:174:PRO:HB3	1.73	0.53
1:A:228:ASP:OD2	1:A:228:ASP:N	2.42	0.53
1:A:472:ILE:HD11	1:A:488:CYS:HB3	1.88	0.53
2:B:303:LEU:HD12	2:B:308:VAL:HG12	1.90	0.53
7:G:2:NAG:H83	7:G:2:NAG:H3	1.89	0.53
3:C:336:CYS:SG	3:C:524:VAL:HG22	2.49	0.53
3:C:870:ILE:O	3:C:874:THR:HG23	2.09	0.53
1:A:1139:ASP:OD1	1:A:1139:ASP:N	2.39	0.52
3:C:565:PHE:HB3	3:C:576:VAL:HG23	1.91	0.52
4:P:45:LEU:HD11	5:S:44:PRO:HG2	1.92	0.52
3:C:193:VAL:HB	3:C:204:TYR:HB2	1.92	0.52
3:C:364:ASP:OD2	3:C:527:PRO:CG	2.58	0.52
1:A:328:ARG:HD3	1:A:578:ASP:OD1	2.09	0.52
2:B:309:GLU:HG2	2:B:313:TYR:OH	2.10	0.52
2:B:400:PHE:HE2	2:B:410:ILE:HD12	1.75	0.52
3:C:295:PRO:O	3:C:299:THR:HG23	2.09	0.52
5:Q:6:GLN:HE22	5:Q:88:CYS:H	1.56	0.52
5:S:34:HIS:HE1	5:S:50:TYR:CE2	2.27	0.52
3:C:388:ASN:O	3:C:526:GLY:CA	2.57	0.52
4:P:93:VAL:HG12	4:P:114:LEU:HD23	1.91	0.52
1:A:362:VAL:HG12	1:A:527:PRO:CB	2.39	0.52
1:A:395:VAL:CB	1:A:524:VAL:HG21	2.39	0.52
1:A:472:ILE:HD13	1:A:484:LYS:HG3	1.91	0.52
2:B:567:ARG:HH21	2:B:571:ASP:HA	1.74	0.52
5:V:6:GLN:HE22	5:V:88:CYS:H	1.56	0.52
1:A:973:ILE:HG23	1:A:992:GLN:OE1	2.10	0.52
2:B:391:CYS:O	2:B:522:ALA:HB2	2.10	0.52
3:C:532:ASN:OD1	3:C:533:LEU:N	2.42	0.52
4:U:93:VAL:HG12	4:U:114:LEU:HD23	1.91	0.52
2:B:334:ASN:ND2	2:B:360:ASN:O	2.43	0.51
3:C:856:ASN:ND2	3:C:856:ASN:O	2.43	0.51
5:V:34:HIS:HE1	5:V:50:TYR:CE1	2.27	0.51
1:A:393:THR:HB	1:A:516:GLU:HB2	1.91	0.51
3:C:560:LEU:N	3:C:563:GLN:OE1	2.43	0.51
4:U:50:TRP:CD2	4:U:104:TRP:HH2	2.28	0.51
1:A:393:THR:CB	1:A:516:GLU:HB2	2.37	0.51
2:B:472:ILE:HD13	2:B:484:LYS:HG3	1.91	0.51
4:U:37:VAL:CG2	4:U:95:TYR:HB2	2.40	0.51
4:O:48:MET:HG2	4:O:64:PHE:CD1	2.46	0.51

Continued on next page...

PRELIMINARY VALIDATION REPORT

Continued from previous page...

Atom-1	Atom-2	Interatomic distance (Å)	Clash overlap (Å)
2:B:564:GLN:HG3	2:B:565:PHE:N	2.26	0.51
4:O:45:LEU:HD11	5:Q:44:PRO:HG2	1.92	0.51
4:O:93:VAL:HG12	4:O:114:LEU:HD23	1.91	0.51
4:P:48:MET:HG2	4:P:64:PHE:CD1	2.46	0.51
4:P:50:TRP:CD2	4:P:104:TRP:HH2	2.28	0.51
1:A:983:ARG:HD2	3:C:382:VAL:HG13	1.92	0.51
3:C:746:SER:HB3	3:C:749:CYS:SG	2.51	0.51
4:U:45:LEU:HD11	5:V:44:PRO:HG2	1.92	0.51
4:O:37:VAL:CG2	4:O:95:TYR:HB2	2.40	0.51
4:P:37:VAL:CG2	4:P:95:TYR:HB2	2.40	0.51
1:A:986:PRO:O	1:A:990:GLU:HG3	2.11	0.51
4:U:37:VAL:HG22	4:U:95:TYR:HB2	1.93	0.51
4:O:50:TRP:CD2	4:O:104:TRP:HH2	2.28	0.51
2:B:316:SER:OG	2:B:317:ASN:N	2.43	0.51
2:B:390:LEU:HD22	3:C:983:ARG:HD3	1.92	0.51
3:C:189:LEU:HD23	3:C:208:THR:O	2.11	0.51
1:A:217:PRO:C	1:A:219:GLY:H	2.14	0.51
1:A:787:GLN:OE1	3:C:703:ASN:HB2	2.11	0.51
3:C:400:PHE:HE2	3:C:410:ILE:HD12	1.75	0.51
4:P:35:ASN:OD1	4:P:47:TRP:NE1	2.43	0.51
5:Q:84:ALA:HB3	5:Q:86:TYR:CE1	2.46	0.51
1:A:400:PHE:HE2	1:A:410:ILE:HD12	1.75	0.51
1:A:722:VAL:HG22	1:A:930:ALA:HB1	1.91	0.51
2:B:395:VAL:HG21	2:B:524:VAL:CG1	2.41	0.51
4:U:48:MET:HG2	4:U:64:PHE:CD1	2.46	0.51
2:B:200:TYR:HE1	2:B:230:PRO:HB3	1.77	0.50
2:B:715:PRO:HA	2:B:1072:GLU:HA	1.93	0.50
3:C:298:GLU:OE2	3:C:316:SER:OG	2.29	0.50
4:O:35:ASN:OD1	4:O:47:TRP:NE1	2.43	0.50
3:C:200:TYR:CE1	3:C:230:PRO:HB3	2.46	0.50
5:V:2:ILE:HD13	5:V:27:GLN:HB2	1.93	0.50
4:O:37:VAL:HG22	4:O:95:TYR:HB2	1.93	0.50
5:Q:2:ILE:HD13	5:Q:27:GLN:HB2	1.94	0.50
1:A:132:GLU:HG2	1:A:165:ASN:HB2	1.94	0.50
1:A:362:VAL:HG12	1:A:527:PRO:HB3	1.94	0.50
8:A:1302:NAG:H83	3:C:558:LYS:NZ	2.26	0.50
3:C:34:ARG:NH1	3:C:191:GLU:OE1	2.44	0.50
4:U:35:ASN:OD1	4:U:47:TRP:NE1	2.43	0.50
5:V:84:ALA:HB3	5:V:86:TYR:CE1	2.46	0.50
3:C:391:CYS:SG	3:C:544:ASN:CB	3.00	0.50
4:P:37:VAL:HG22	4:P:95:TYR:HB2	1.93	0.50

Continued on next page...

PRELIMINARY VALIDATION REPORT

Continued from previous page...

Atom-1	Atom-2	Interatomic distance (Å)	Clash overlap (Å)
2:B:362:VAL:HA	2:B:525:CYS:H	1.76	0.50
3:C:105:ILE:HG12	3:C:241:LEU:HD21	1.93	0.50
3:C:470:THR:HG22	3:C:492:LEU:HD12	1.94	0.50
4:O:38:ARG:HD3	4:O:64:PHE:CE2	2.47	0.50
5:S:2:ILE:HD13	5:S:27:GLN:HB2	1.93	0.50
3:C:330:PRO:C	3:C:332:ILE:N	2.61	0.50
5:Q:36:TYR:CD1	5:Q:46:LEU:HA	2.47	0.50
1:A:869:MET:HG2	3:C:699:LEU:HD21	1.94	0.49
1:A:359:SER:HA	1:A:523:THR:HG23	1.95	0.49
1:A:391:CYS:CB	1:A:525:CYS:SG	3.00	0.49
1:A:746:SER:HB3	1:A:749:CYS:SG	2.52	0.49
5:V:21:ILE:O	5:V:72:THR:OG1	2.19	0.49
1:A:360:ASN:HA	1:A:523:THR:OG1	2.12	0.49
1:A:963:VAL:HG12	3:C:570:ALA:HB2	1.93	0.49
3:C:391:CYS:SG	3:C:544:ASN:HB3	2.52	0.49
4:P:38:ARG:HD3	4:P:64:PHE:CE2	2.47	0.49
1:A:473:TYR:CE2	1:A:475:ALA:HB2	2.48	0.49
3:C:206:LYS:HB2	3:C:223:LEU:HD22	1.93	0.49
4:U:34:MET:HG2	4:U:79:ALA:CB	2.42	0.49
4:U:38:ARG:HD3	4:U:64:PHE:CE2	2.47	0.49
1:A:470:THR:HG22	1:A:492:LEU:HD12	1.94	0.49
2:B:386:LYS:HE2	3:C:981:LEU:O	2.12	0.49
2:B:390:LEU:CD2	3:C:983:ARG:HD3	2.42	0.49
3:C:170:TYR:CE2	3:C:172:SER:HB2	2.48	0.49
3:C:280:ASN:ND2	8:C:1301:NAG:H82	2.27	0.49
3:C:429:PHE:HE1	3:C:514:SER:HA	1.77	0.49
4:O:34:MET:HG2	4:O:79:ALA:CB	2.42	0.49
1:A:578:ASP:OD2	1:A:581:THR:HG22	2.13	0.49
1:A:858:LEU:HD23	1:A:959:LEU:HD22	1.95	0.49
1:A:870:ILE:O	1:A:874:THR:HG23	2.12	0.49
4:P:34:MET:HG2	4:P:79:ALA:CB	2.42	0.49
5:S:84:ALA:HB3	5:S:86:TYR:CE2	2.46	0.49
1:A:331:ASN:HB2	1:A:580:GLN:HG2	1.93	0.49
2:B:97:LYS:HZ2	2:B:187:LYS:N	2.11	0.49
3:C:391:CYS:O	3:C:522:ALA:CB	2.61	0.49
3:C:472:ILE:HG21	3:C:482:GLY:H	1.78	0.48
5:S:35:TRP:CD2	5:S:73:LEU:HD22	2.48	0.48
2:B:473:TYR:CE2	2:B:475:ALA:HB2	2.48	0.48
5:Q:35:TRP:CD2	5:Q:73:LEU:HD22	2.48	0.48
2:B:470:THR:HG22	2:B:492:LEU:HD12	1.94	0.48
4:O:107:ASP:HB3	4:O:109:TRP:CE3	2.49	0.48

Continued on next page...

PRELIMINARY VALIDATION REPORT

Continued from previous page...

Atom-1	Atom-2	Interatomic distance (Å)	Clash overlap (Å)
3:C:124:THR:HG22	3:C:174:PRO:HG3	1.95	0.48
3:C:391:CYS:HB2	3:C:544:ASN:C	2.27	0.48
3:C:473:TYR:CE2	3:C:475:ALA:HB2	2.48	0.48
1:A:46:SER:CA	1:A:279:TYR:O	2.61	0.48
1:A:472:ILE:HG21	1:A:482:GLY:H	1.78	0.48
3:C:567:ARG:NH1	3:C:571:ASP:OD1	2.47	0.48
1:A:976:VAL:HB	1:A:979:ASP:OD2	2.14	0.48
2:B:472:ILE:HG21	2:B:482:GLY:H	1.78	0.48
6:E:1:NAG:H4	6:E:2:NAG:H2	1.63	0.48
2:B:359:SER:C	2:B:523:THR:HG23	2.15	0.48
4:P:107:ASP:HB3	4:P:109:TRP:CE3	2.49	0.48
1:A:357:ARG:NH2	2:B:167:THR:HA	2.28	0.48
2:B:395:VAL:CG2	2:B:524:VAL:CG1	2.92	0.48
4:U:4:LEU:HB2	4:U:109:TRP:HD1	1.76	0.48
2:B:334:ASN:HD22	2:B:361:CYS:CB	2.27	0.48
3:C:725:GLU:OE1	3:C:1028:LYS:NZ	2.22	0.48
4:U:2:VAL:HG23	4:U:2:VAL:O	2.13	0.48
5:V:35:TRP:CD2	5:V:73:LEU:HD22	2.48	0.48
3:C:170:TYR:CZ	3:C:172:SER:HB2	2.49	0.48
4:O:2:VAL:HG23	4:O:2:VAL:O	2.14	0.48
4:P:4:LEU:HD23	4:P:22:CYS:SG	2.54	0.48
1:A:395:VAL:HB	1:A:524:VAL:HG21	1.95	0.47
1:A:1031:GLU:N	1:A:1031:GLU:OE1	2.47	0.47
4:U:107:ASP:HB3	4:U:109:TRP:CE3	2.49	0.47
1:A:1105:THR:HG22	1:A:1112:PRO:HA	1.96	0.47
4:P:2:VAL:HG23	4:P:2:VAL:O	2.13	0.47
3:C:200:TYR:HE1	3:C:230:PRO:HB3	1.80	0.47
3:C:206:LYS:HG3	3:C:223:LEU:HA	1.95	0.47
4:O:4:LEU:HD23	4:O:22:CYS:SG	2.54	0.47
1:A:331:ASN:CB	1:A:580:GLN:HG2	2.45	0.47
1:A:1102:TRP:HB2	1:A:1135:ASN:ND2	2.30	0.47
5:V:36:TYR:CD1	5:V:46:LEU:HA	2.47	0.47
1:A:200:TYR:CZ	3:C:396:TYR:OH	2.68	0.47
3:C:331:ASN:O	3:C:332:ILE:C	2.52	0.47
4:U:109:TRP:CD1	4:U:109:TRP:O	2.68	0.47
6:D:1:NAG:H4	6:D:2:NAG:H2	1.63	0.47
2:B:324:GLU:OE1	2:B:325:SER:N	2.40	0.47
5:V:4:MET:HE3	5:V:90:GLN:HB3	1.97	0.47
6:F:1:NAG:H4	6:F:2:NAG:H2	1.63	0.47
1:A:395:VAL:HG23	1:A:524:VAL:CB	2.31	0.47
2:B:386:LYS:CD	3:C:984:LEU:N	2.75	0.47

Continued on next page...

PRELIMINARY VALIDATION REPORT

Continued from previous page...

Atom-1	Atom-2	Interatomic distance (Å)	Clash overlap (Å)
2:B:786:LYS:HG3	2:B:787:GLN:HG3	1.97	0.47
3:C:715:PRO:HA	3:C:1071:GLN:O	2.13	0.47
5:V:61:ARG:NE	5:V:79:GLN:OE1	2.48	0.47
4:P:4:LEU:HB2	4:P:109:TRP:HD1	1.77	0.47
1:A:99:ASN:CG	1:A:102:ARG:HE	2.19	0.47
5:Q:61:ARG:NE	5:Q:79:GLN:OE1	2.48	0.47
1:A:394:ASN:CB	1:A:516:GLU:CB	2.93	0.46
1:A:1037:SER:OG	1:A:1039:ARG:HG2	2.14	0.46
2:B:452:LEU:HD22	2:B:494:SER:HA	1.98	0.46
4:U:59:THR:HG23	4:U:59:THR:O	2.15	0.46
5:Q:55:ILE:HG23	5:Q:58:VAL:HB	1.97	0.46
1:A:611:LEU:HD22	1:A:666:ILE:HG23	1.97	0.46
1:A:726:ILE:HG12	1:A:1061:VAL:HG22	1.97	0.46
2:B:363:ALA:O	2:B:525:CYS:C	2.53	0.46
3:C:431:GLY:HA2	3:C:515:PHE:CD2	2.50	0.46
4:O:109:TRP:CD1	4:O:109:TRP:O	2.68	0.46
1:A:770:ILE:O	1:A:774:GLN:HG2	2.15	0.46
2:B:363:ALA:O	2:B:526:GLY:N	2.49	0.46
2:B:418:ILE:HA	2:B:422:ASN:HB2	1.98	0.46
4:U:37:VAL:O	4:U:95:TYR:N	2.45	0.46
5:Q:4:MET:HE3	5:Q:90:GLN:HB3	1.97	0.46
4:P:14:PRO:HG3	4:P:117:VAL:HG22	1.97	0.46
4:U:4:LEU:HD23	4:U:22:CYS:SG	2.54	0.46
4:U:67:ARG:HD2	4:U:84:CYS:O	2.15	0.46
4:O:95:TYR:HE2	5:Q:44:PRO:HD2	1.81	0.46
2:B:280:ASN:OD1	2:B:281:GLU:N	2.42	0.46
4:O:67:ARG:HD2	4:O:84:CYS:O	2.15	0.46
5:S:61:ARG:NE	5:S:79:GLN:OE1	2.48	0.46
2:B:722:VAL:HA	2:B:1064:HIS:O	2.14	0.46
3:C:418:ILE:HA	3:C:422:ASN:HB2	1.98	0.46
1:A:282:ASN:HB2	1:A:284:THR:HG22	1.98	0.46
4:U:12:LYS:HG3	4:U:18:VAL:CG1	2.30	0.46
4:O:37:VAL:O	4:O:95:TYR:N	2.45	0.46
4:P:67:ARG:HD2	4:P:84:CYS:O	2.15	0.46
1:A:418:ILE:HA	1:A:422:ASN:HB2	1.98	0.46
3:C:454:ARG:HE	3:C:454:ARG:HB3	1.59	0.46
5:V:55:ILE:HG23	5:V:58:VAL:HB	1.98	0.46
4:P:109:TRP:CD1	4:P:109:TRP:O	2.68	0.46
5:S:55:ILE:HG23	5:S:58:VAL:HB	1.98	0.46
1:A:320:VAL:HG22	1:A:591:SER:O	2.16	0.46
1:A:394:ASN:ND2	1:A:516:GLU:CA	2.79	0.46

Continued on next page...

PRELIMINARY VALIDATION REPORT

Continued from previous page...

Atom-1	Atom-2	Interatomic distance (Å)	Clash overlap (Å)
1:A:516:GLU:CB	1:A:516:GLU:CA	2.94	0.46
1:A:542:ASN:OD1	1:A:547:THR:HG22	2.16	0.46
4:P:59:THR:HG23	4:P:59:THR:O	2.15	0.46
2:B:53:ASP:HB3	2:B:55:PHE:CE1	2.51	0.45
2:B:734:THR:HG21	2:B:1007:TYR:OH	2.16	0.45
5:S:36:TYR:CD2	5:S:46:LEU:HA	2.47	0.45
4:O:59:THR:HG23	4:O:59:THR:O	2.15	0.45
2:B:390:LEU:HD11	3:C:983:ARG:CB	2.46	0.45
4:U:20:VAL:HG12	4:U:113:THR:HG21	1.98	0.45
1:A:957:GLN:HE21	2:B:765:ARG:NH2	2.14	0.45
3:C:126:VAL:CG2	3:C:172:SER:HB3	2.46	0.45
3:C:725:GLU:CD	3:C:1028:LYS:HZ3	2.15	0.45
4:O:20:VAL:HG12	4:O:113:THR:HG21	1.98	0.45
1:A:359:SER:HB3	1:A:523:THR:HG21	1.98	0.45
2:B:362:VAL:CA	2:B:525:CYS:O	2.64	0.45
2:B:362:VAL:HG13	2:B:525:CYS:O	2.16	0.45
3:C:973:ILE:HG23	3:C:992:GLN:OE1	2.17	0.45
4:U:14:PRO:HG3	4:U:117:VAL:HG22	1.97	0.45
4:U:95:TYR:HE2	5:V:44:PRO:HD2	1.81	0.45
1:A:458:LYS:HA	1:A:458:LYS:HD2	1.41	0.45
1:A:452:LEU:HD22	1:A:494:SER:HA	1.98	0.45
3:C:452:LEU:HD22	3:C:494:SER:HA	1.98	0.45
1:A:707:TYR:HB2	2:B:883:THR:CG2	2.46	0.45
3:C:271:GLN:OE1	3:C:272:PRO:HD2	2.17	0.45
3:C:977:LEU:HD11	3:C:993:ILE:HG12	1.97	0.45
4:U:51:ILE:O	4:U:51:ILE:HG13	2.17	0.45
4:O:4:LEU:HD13	4:O:109:TRP:NE1	2.21	0.45
4:P:20:VAL:HG12	4:P:113:THR:HG21	1.98	0.45
2:B:934:ILE:HD13	2:B:1063:LEU:HD13	1.99	0.45
3:C:106:PHE:HD2	3:C:235:ILE:HD11	1.82	0.45
3:C:531:THR:HG22	3:C:532:ASN:N	2.32	0.45
3:C:560:LEU:HB2	3:C:563:GLN:NE2	2.32	0.45
5:Q:21:ILE:O	5:Q:72:THR:OG1	2.19	0.45
4:P:51:ILE:HG22	4:P:58:PRO:HA	1.99	0.45
3:C:332:ILE:C	3:C:334:ASN:H	2.21	0.45
3:C:914:ASN:O	3:C:918:GLU:HG3	2.16	0.45
4:O:14:PRO:HG3	4:O:117:VAL:HG22	1.97	0.45
1:A:345:THR:HG23	4:U:104:TRP:HD1	1.82	0.44
1:A:736:VAL:HG12	1:A:858:LEU:HG	1.99	0.44
3:C:345:THR:HG23	4:P:104:TRP:HD1	1.83	0.44
4:U:51:ILE:HG22	4:U:58:PRO:HA	1.99	0.44

Continued on next page...

PRELIMINARY VALIDATION REPORT

Continued from previous page...

Atom-1	Atom-2	Interatomic distance (Å)	Clash overlap (Å)
4:P:95:TYR:HE2	5:S:44:PRO:HD2	1.81	0.44
2:B:357:ARG:HE	3:C:230:PRO:HG3	1.36	0.44
2:B:702:GLU:O	2:B:703:ASN:HB3	2.18	0.44
5:V:34:HIS:CE1	5:V:50:TYR:CD1	3.05	0.44
5:Q:90:GLN:OE1	5:Q:92:ASN:N	2.44	0.44
1:A:364:ASP:HB2	1:A:527:PRO:HD3	1.98	0.44
2:B:363:ALA:O	2:B:525:CYS:O	2.36	0.44
5:Q:24:ARG:HB2	5:Q:24:ARG:CZ	2.47	0.44
5:Q:61:ARG:HD3	5:Q:77:SER:O	2.18	0.44
1:A:394:ASN:O	1:A:515:PHE:C	2.56	0.44
2:B:736:VAL:HG11	2:B:1004:LEU:HD11	1.99	0.44
3:C:29:THR:HG22	3:C:62:VAL:HG23	2.00	0.44
3:C:112:SER:HB2	3:C:134:GLN:HG2	1.99	0.44
5:V:24:ARG:NH2	5:V:24:ARG:HB2	2.33	0.44
5:V:61:ARG:HD3	5:V:77:SER:O	2.18	0.44
5:S:4:MET:HE3	5:S:90:GLN:HB3	1.99	0.44
5:S:24:ARG:HB2	5:S:24:ARG:CZ	2.48	0.44
5:S:24:ARG:HB2	5:S:24:ARG:NH2	2.33	0.44
3:C:434:ILE:HD11	3:C:513:LEU:HD12	2.00	0.44
4:O:51:ILE:HG13	4:O:51:ILE:O	2.17	0.44
1:A:811:LYS:HE2	1:A:811:LYS:HB2	1.72	0.44
5:Q:79:GLN:HA	5:Q:80:PRO:HD2	1.87	0.44
4:P:51:ILE:HG13	4:P:51:ILE:O	2.17	0.44
2:B:434:ILE:HD11	2:B:513:LEU:HD12	2.00	0.44
2:B:345:THR:HG23	4:O:104:TRP:HD1	1.83	0.43
2:B:492:LEU:HA	2:B:492:LEU:HD23	1.85	0.43
3:C:105:ILE:HD12	3:C:118:LEU:HG	1.99	0.43
4:O:51:ILE:HG22	4:O:58:PRO:HA	1.99	0.43
5:S:34:HIS:CE1	5:S:50:TYR:CD2	3.05	0.43
1:A:121:ASN:HD21	1:A:174:PRO:CB	2.30	0.43
1:A:296:LEU:O	1:A:300:LYS:HG3	2.18	0.43
1:A:421:TYR:HB3	1:A:457:ARG:HB3	2.00	0.43
1:A:492:LEU:HD23	1:A:492:LEU:HA	1.85	0.43
4:O:47:TRP:HZ2	4:O:50:TRP:HD1	1.66	0.43
2:B:714:ILE:HD11	2:B:1096:VAL:HG11	2.00	0.43
5:Q:24:ARG:HB2	5:Q:24:ARG:NH2	2.33	0.43
2:B:91:TYR:OH	2:B:191:GLU:OE1	2.19	0.43
3:C:87:ASN:OD1	3:C:88:ASP:N	2.52	0.43
3:C:421:TYR:HB3	3:C:457:ARG:HB3	2.00	0.43
5:V:24:ARG:HB2	5:V:24:ARG:CZ	2.48	0.43
4:O:38:ARG:HB3	4:O:48:MET:HE1	2.01	0.43

Continued on next page...

PRELIMINARY VALIDATION REPORT

Continued from previous page...

Atom-1	Atom-2	Interatomic distance (Å)	Clash overlap (Å)
1:A:531:THR:HG22	1:A:532:ASN:N	2.33	0.43
4:O:108:VAL:O	4:O:108:VAL:HG13	2.19	0.43
4:P:47:TRP:HZ2	4:P:50:TRP:HD1	1.66	0.43
4:P:108:VAL:HG13	4:P:108:VAL:O	2.19	0.43
1:A:434:ILE:HD11	1:A:513:LEU:HD12	2.00	0.43
3:C:441:LEU:HD13	3:C:441:LEU:HA	1.91	0.43
1:A:410:ILE:H	1:A:410:ILE:HG12	1.71	0.43
1:A:822:LEU:HD21	1:A:938:LEU:HD13	2.01	0.43
2:B:296:LEU:O	2:B:299:THR:OG1	2.28	0.43
3:C:410:ILE:H	3:C:410:ILE:HG12	1.71	0.43
5:V:6:GLN:NE2	5:V:88:CYS:H	2.17	0.43
2:B:421:TYR:HB3	2:B:457:ARG:HB3	2.00	0.43
2:B:455:LEU:HB3	2:B:456:PHE:H	1.51	0.43
3:C:391:CYS:CB	3:C:544:ASN:C	2.85	0.43
5:S:61:ARG:HD3	5:S:77:SER:O	2.18	0.43
1:A:299:THR:HA	1:A:302:THR:HG22	2.00	0.43
1:A:707:TYR:CD1	2:B:883:THR:HG23	2.54	0.43
1:A:912:THR:OG1	1:A:914:ASN:ND2	2.52	0.43
3:C:276:LEU:O	3:C:288:ALA:HA	2.19	0.43
4:O:4:LEU:HD11	4:O:27:TYR:OH	2.19	0.43
5:Q:6:GLN:NE2	5:Q:88:CYS:H	2.17	0.43
5:Q:6:GLN:OE1	5:Q:102:THR:HG23	2.19	0.43
4:P:4:LEU:HD11	4:P:27:TYR:OH	2.19	0.43
1:A:394:ASN:OD1	1:A:516:GLU:C	2.56	0.43
2:B:914:ASN:O	2:B:918:GLU:HG3	2.18	0.43
2:B:985:ASP:OD2	2:B:987:PRO:HD2	2.18	0.43
3:C:57:PRO:HB3	3:C:273:ARG:HH21	1.84	0.43
4:O:4:LEU:HB2	4:O:109:TRP:HD1	1.77	0.43
4:O:39:GLN:HG3	4:O:45:LEU:HD23	2.01	0.43
2:B:821:LEU:O	2:B:825:LYS:HG2	2.19	0.42
5:S:6:GLN:OE1	5:S:102:THR:HG23	2.19	0.42
5:S:84:ALA:O	5:S:86:TYR:HD2	2.02	0.42
2:B:129:LYS:HG2	2:B:169:GLU:HG2	2.01	0.42
2:B:201:PHE:HE2	2:B:203:ILE:HD11	1.84	0.42
3:C:564:GLN:OE1	3:C:577:ARG:HD2	2.19	0.42
5:S:34:HIS:CE1	5:S:50:TYR:HD2	2.38	0.42
1:A:199:GLY:HA2	1:A:232:GLY:HA2	2.01	0.42
1:A:320:VAL:HG23	1:A:590:CYS:HB3	2.01	0.42
1:A:328:ARG:NH1	1:A:578:ASP:OD2	2.53	0.42
1:A:360:ASN:H	1:A:523:THR:HG23	1.85	0.42
1:A:393:THR:OG1	1:A:516:GLU:C	2.58	0.42

Continued on next page...

PRELIMINARY VALIDATION REPORT

Continued from previous page...

Atom-1	Atom-2	Interatomic distance (Å)	Clash overlap (Å)
3:C:205:SER:O	3:C:206:LYS:HG2	2.20	0.42
3:C:336:CYS:SG	3:C:524:VAL:HG13	2.59	0.42
3:C:425:LEU:HD12	3:C:425:LEU:HA	1.79	0.42
5:V:6:GLN:OE1	5:V:102:THR:HG23	2.19	0.42
4:P:35:ASN:ND2	4:P:35:ASN:H	2.18	0.42
1:A:790:LYS:H	3:C:704:SER:HA	1.84	0.42
2:B:332:ILE:O	2:B:362:VAL:HG23	2.20	0.42
2:B:381:GLY:O	3:C:984:LEU:HD11	2.19	0.42
2:B:555:SER:HB3	2:B:586:ASP:HB2	2.01	0.42
2:B:856:ASN:OD1	2:B:856:ASN:N	2.50	0.42
4:U:35:ASN:ND2	4:U:35:ASN:H	2.18	0.42
4:U:108:VAL:O	4:U:108:VAL:HG13	2.19	0.42
5:V:79:GLN:HA	5:V:80:PRO:HD2	1.86	0.42
5:V:84:ALA:O	5:V:86:TYR:HD1	2.02	0.42
1:A:391:CYS:HB3	1:A:522:ALA:HB1	2.02	0.42
2:B:39:PRO:HG3	2:B:51:THR:HG21	2.02	0.42
2:B:48:LEU:CD2	2:B:278:LYS:HG3	2.50	0.42
2:B:396:TYR:OH	3:C:230:PRO:HB3	2.19	0.42
3:C:402:ILE:H	3:C:402:ILE:HG13	1.77	0.42
1:A:29:THR:HG22	1:A:30:ASN:N	2.34	0.42
2:B:382:VAL:O	3:C:984:LEU:HD21	2.15	0.42
2:B:916:LEU:HD12	2:B:923:ILE:HD12	2.02	0.42
4:U:38:ARG:HB3	4:U:48:MET:HE1	2.02	0.42
5:V:34:HIS:CE1	5:V:50:TYR:HD1	2.38	0.42
5:V:62:PHE:HB3	5:V:75:ILE:HA	2.02	0.42
5:Q:84:ALA:O	5:Q:86:TYR:HD1	2.02	0.42
5:S:37:GLN:HB3	5:S:47:LEU:HD11	2.02	0.42
1:A:326:ILE:O	1:A:326:ILE:HG13	2.19	0.42
2:B:583:GLU:HG2	2:B:584:ILE:H	1.84	0.42
3:C:280:ASN:CG	3:C:281:GLU:H	2.23	0.42
3:C:363:ALA:H	3:C:525:CYS:H	1.67	0.42
3:C:558:LYS:O	3:C:558:LYS:HG3	2.20	0.42
3:C:33:THR:HA	3:C:58:PHE:CD1	2.55	0.42
3:C:105:ILE:CD1	3:C:118:LEU:HG	2.50	0.42
4:U:34:MET:SD	4:U:96:CYS:SG	3.18	0.42
4:U:47:TRP:HZ2	4:U:50:TRP:HD1	1.66	0.42
5:Q:34:HIS:CE1	5:Q:50:TYR:HD1	2.38	0.42
4:P:37:VAL:O	4:P:95:TYR:N	2.45	0.42
5:S:3:GLN:OE1	5:S:3:GLN:HA	2.20	0.42
5:S:11:LEU:H	5:S:11:LEU:HD23	1.85	0.42
1:A:809:PRO:HA	1:A:814:LYS:HE3	2.01	0.42

Continued on next page...

PRELIMINARY VALIDATION REPORT

Continued from previous page...

Atom-1	Atom-2	Interatomic distance (Å)	Clash overlap (Å)
4:U:4:LEU:HD11	4:U:27:TYR:OH	2.19	0.42
5:V:3:GLN:HA	5:V:3:GLN:OE1	2.20	0.42
4:O:104:TRP:CE2	5:Q:94:TRP:HH2	2.38	0.42
5:Q:62:PHE:HB3	5:Q:75:ILE:HA	2.02	0.42
7:L:1:NAG:H61	7:L:2:NAG:H82	2.02	0.42
2:B:368:LEU:N	2:B:368:LEU:CD1	2.83	0.41
1:A:402:ILE:H	1:A:402:ILE:HG13	1.77	0.41
2:B:126:VAL:HG13	2:B:172:SER:HB3	2.03	0.41
2:B:552:LEU:HA	2:B:586:ASP:O	2.19	0.41
3:C:1102:TRP:CZ2	3:C:1133:VAL:HG21	2.55	0.41
4:U:39:GLN:HG3	4:U:45:LEU:HD23	2.01	0.41
4:O:34:MET:SD	4:O:96:CYS:SG	3.18	0.41
1:A:217:PRO:C	1:A:219:GLY:N	2.73	0.41
1:A:394:ASN:O	1:A:515:PHE:HA	2.20	0.41
1:A:868:GLU:OE2	1:A:868:GLU:N	2.51	0.41
4:U:35:ASN:ND2	4:U:99:GLY:O	2.54	0.41
4:U:62:ASP:HA	4:U:65:ARG:HH21	1.85	0.41
4:U:104:TRP:CE2	5:V:94:TRP:HH2	2.39	0.41
4:P:34:MET:SD	4:P:96:CYS:SG	3.18	0.41
4:P:39:GLN:HG3	4:P:45:LEU:HD23	2.01	0.41
2:B:577:ARG:HA	2:B:583:GLU:O	2.20	0.41
5:Q:11:LEU:HD23	5:Q:11:LEU:H	1.85	0.41
1:A:975:SER:OG	3:C:571:ASP:OD2	2.38	0.41
2:B:1107:ARG:HD2	3:C:904:TYR:CE2	2.55	0.41
3:C:31:SER:O	3:C:59:PHE:HA	2.20	0.41
5:V:11:LEU:HD23	5:V:11:LEU:H	1.85	0.41
4:P:35:ASN:ND2	4:P:99:GLY:O	2.54	0.41
2:B:562:PHE:HB2	3:C:41:LYS:HE2	2.01	0.41
2:B:733:LYS:HE3	2:B:771:ALA:O	2.21	0.41
3:C:114:THR:HG22	3:C:115:GLN:N	2.35	0.41
3:C:901:GLN:O	3:C:905:ARG:HG3	2.20	0.41
2:B:382:VAL:CB	3:C:983:ARG:HD2	2.49	0.41
2:B:552:LEU:CD2	2:B:587:ILE:HG12	2.51	0.41
2:B:741:TYR:CE1	2:B:966:LEU:HD11	2.56	0.41
2:B:1027:THR:HG22	2:B:1042:PHE:HZ	1.85	0.41
1:A:134:GLN:NE2	1:A:162:SER:OG	2.32	0.41
1:A:386:LYS:H	1:A:386:LYS:HG3	1.58	0.41
2:B:363:ALA:H	2:B:525:CYS:C	2.24	0.41
2:B:437:ASN:HB2	2:B:508:TYR:CE2	2.56	0.41
2:B:980:ILE:HG13	2:B:981:LEU:N	2.36	0.41
3:C:437:ASN:HB2	3:C:508:TYR:CE1	2.56	0.41

Continued on next page...

PRELIMINARY VALIDATION REPORT

Continued from previous page...

Atom-1	Atom-2	Interatomic distance (Å)	Clash overlap (Å)
4:U:50:TRP:CG	4:U:104:TRP:HH2	2.39	0.41
4:O:35:ASN:H	4:O:35:ASN:ND2	2.18	0.41
4:O:39:GLN:NE2	4:O:40:ALA:O	2.54	0.41
4:O:60:TYR:CE1	4:O:69:VAL:HA	2.56	0.41
1:A:336:CYS:HA	1:A:337:PRO:HD3	1.95	0.41
1:A:368:LEU:N	1:A:368:LEU:CD1	2.83	0.41
1:A:743:CYS:SG	1:A:750:SER:N	2.93	0.41
1:A:789:TYR:HD1	3:C:703:ASN:O	2.04	0.41
2:B:172:SER:OG	2:B:173:GLN:N	2.53	0.41
2:B:658:ASN:ND2	2:B:660:TYR:CZ	2.89	0.41
2:B:658:ASN:ND2	2:B:660:TYR:OH	2.42	0.41
3:C:461:LEU:HD13	3:C:461:LEU:HA	1.90	0.41
5:V:91:SER:HA	5:V:96:LEU:HD22	2.03	0.41
4:O:50:TRP:CG	4:O:104:TRP:HH2	2.39	0.41
5:Q:3:GLN:HA	5:Q:3:GLN:OE1	2.20	0.41
4:P:62:ASP:HA	4:P:65:ARG:HH21	1.85	0.41
4:P:104:TRP:CE2	5:S:94:TRP:HH2	2.38	0.41
5:S:62:PHE:HB3	5:S:75:ILE:HA	2.02	0.41
1:A:441:LEU:HD13	1:A:441:LEU:HA	1.91	0.41
2:B:583:GLU:HG2	2:B:584:ILE:N	2.36	0.41
4:U:39:GLN:NE2	4:U:40:ALA:O	2.54	0.41
5:V:37:GLN:HB3	5:V:47:LEU:HD11	2.02	0.41
4:O:95:TYR:CE2	5:Q:44:PRO:HD2	2.56	0.41
5:Q:34:HIS:CE1	5:Q:50:TYR:CD1	3.05	0.41
5:Q:37:GLN:HB3	5:Q:47:LEU:HD11	2.02	0.41
4:P:4:LEU:HD13	4:P:109:TRP:NE1	2.21	0.41
5:S:91:SER:HA	5:S:96:LEU:HD22	2.03	0.41
1:A:303:LEU:HD23	1:A:308:VAL:HG12	2.03	0.40
2:B:29:THR:HG22	2:B:30:ASN:N	2.37	0.40
3:C:90:VAL:HG21	3:C:238:PHE:CE1	2.56	0.40
5:V:104:LEU:HD23	5:V:104:LEU:HA	1.91	0.40
4:O:35:ASN:ND2	4:O:99:GLY:O	2.54	0.40
4:P:95:TYR:CE2	5:S:44:PRO:HD2	2.56	0.40
1:A:34:ARG:NH2	1:A:217:PRO:HB2	2.36	0.40
1:A:643:PHE:CE2	1:A:645:THR:HG22	2.56	0.40
3:C:189:LEU:HD12	3:C:216:LEU:HD11	2.03	0.40
5:V:21:ILE:O	5:V:21:ILE:HG13	2.22	0.40
4:O:98:ARG:HH21	4:O:109:TRP:HH2	1.69	0.40
4:P:50:TRP:CG	4:P:104:TRP:HH2	2.39	0.40
5:S:6:GLN:NE2	5:S:88:CYS:H	2.17	0.40
2:B:326:ILE:HG13	2:B:326:ILE:O	2.21	0.40

Continued on next page...

PRELIMINARY VALIDATION REPORT

Continued from previous page...

Atom-1	Atom-2	Interatomic distance (Å)	Clash overlap (Å)
3:C:90:VAL:HG23	3:C:267:VAL:HG13	2.02	0.40
3:C:386:LYS:H	3:C:386:LYS:HG3	1.59	0.40
3:C:959:LEU:HD23	3:C:959:LEU:HA	1.89	0.40
5:V:61:ARG:HG3	5:V:62:PHE:CD2	2.57	0.40
5:S:61:ARG:HG3	5:S:62:PHE:CD1	2.57	0.40
2:B:280:ASN:CG	2:B:281:GLU:N	2.75	0.40
2:B:410:ILE:H	2:B:410:ILE:HG12	1.71	0.40
2:B:560:LEU:N	2:B:563:GLN:OE1	2.55	0.40
4:U:95:TYR:CE2	5:V:44:PRO:HD2	2.56	0.40
5:S:9:SER:O	5:S:102:THR:HA	2.22	0.40
7:M:1:NAG:O4	7:M:2:NAG:O7	2.39	0.40
1:A:914:ASN:O	1:A:918:GLU:HG3	2.22	0.40
2:B:805:ILE:HD11	2:B:931:ILE:HD12	2.03	0.40
3:C:368:LEU:N	3:C:368:LEU:CD1	2.83	0.40

There are no symmetry-related clashes.

5.3 Torsion angles (i)

5.3.1 Protein backbone (i)

In the following table, the Percentiles column shows the percent Ramachandran outliers of the chain as a percentile score with respect to all PDB entries followed by that with respect to all EM entries.

The Analysed column shows the number of residues for which the backbone conformation was analysed, and the total number of residues.

Mol	Chain	Analysed	Favoured	Allowed	Outliers	Percentiles	
1	A	988/1016 (97%)	901 (91%)	74 (8%)	13 (1%)	12	47
2	B	982/1000 (98%)	891 (91%)	80 (8%)	11 (1%)	14	50
3	C	982/1000 (98%)	893 (91%)	76 (8%)	13 (1%)	12	47
4	O	116/118 (98%)	94 (81%)	22 (19%)	0	100	100
4	P	116/118 (98%)	94 (81%)	22 (19%)	0	100	100
4	U	116/118 (98%)	94 (81%)	22 (19%)	0	100	100
5	Q	105/107 (98%)	92 (88%)	13 (12%)	0	100	100
5	S	105/107 (98%)	92 (88%)	13 (12%)	0	100	100
5	V	105/107 (98%)	92 (88%)	13 (12%)	0	100	100

Continued on next page...

PRELIMINARY VALIDATION REPORT

Continued from previous page...

Mol	Chain	Analysed	Favoured	Allowed	Outliers	Percentiles	
All	All	3615/3691 (98%)	3243 (90%)	335 (9%)	37 (1%)	20	51

All (37) Ramachandran outliers are listed below:

Mol	Chain	Res	Type
1	A	378	LYS
1	A	516	GLU
1	A	613	GLN
2	B	32	PHE
2	B	378	LYS
3	C	332	ILE
3	C	378	LYS
1	A	32	PHE
1	A	110	LEU
1	A	422	ASN
1	A	492	LEU
2	B	422	ASN
2	B	492	LEU
2	B	703	ASN
3	C	422	ASN
3	C	492	LEU
3	C	516	GLU
3	C	518	LEU
1	A	385	THR
1	A	477	SER
1	A	491	PRO
2	B	385	THR
2	B	477	SER
2	B	491	PRO
3	C	385	THR
3	C	477	SER
3	C	491	PRO
3	C	333	THR
1	A	218	GLN
2	B	518	LEU
3	C	481	ASN
1	A	463	PRO
2	B	463	PRO
3	C	463	PRO
2	B	445	VAL
1	A	445	VAL
3	C	445	VAL

PRELIMINARY VALIDATION REPORT

5.3.2 Protein sidechains (i)

In the following table, the Percentiles column shows the percent sidechain outliers of the chain as a percentile score with respect to all PDB entries followed by that with respect to all EM entries.

The Analysed column shows the number of residues for which the sidechain conformation was analysed, and the total number of residues.

Mol	Chain	Analysed	Rotameric	Outliers	Percentiles	
1	A	857/880 (97%)	783 (91%)	74 (9%)	10	39
2	B	858/877 (98%)	784 (91%)	74 (9%)	10	39
3	C	844/875 (96%)	764 (90%)	80 (10%)	8	34
4	O	97/97 (100%)	97 (100%)	0	100	100
4	P	97/97 (100%)	97 (100%)	0	100	100
4	U	97/97 (100%)	97 (100%)	0	100	100
5	Q	94/94 (100%)	94 (100%)	0	100	100
5	S	94/94 (100%)	94 (100%)	0	100	100
5	V	94/94 (100%)	94 (100%)	0	100	100
All	All	3132/3205 (98%)	2904 (93%)	228 (7%)	18	44

All (228) residues with a non-rotameric sidechain are listed below:

Mol	Chain	Res	Type
1	A	31	SER
1	A	33	THR
1	A	80	ASP
1	A	157	PHE
1	A	206	LYS
1	A	208	THR
1	A	218	GLN
1	A	284	THR
1	A	331	ASN
1	A	332	ILE
1	A	333	THR
1	A	334	ASN
1	A	335	LEU
1	A	356	LYS
1	A	367	VAL
1	A	376	THR
1	A	378	LYS
1	A	382	VAL

Continued on next page...

PRELIMINARY VALIDATION REPORT

Continued from previous page...

Mol	Chain	Res	Type
1	A	386	LYS
1	A	387	LEU
1	A	389	ASP
1	A	394	ASN
1	A	398	ASP
1	A	402	ILE
1	A	405	ASP
1	A	407	VAL
1	A	408	ARG
1	A	409	GLN
1	A	410	ILE
1	A	422	ASN
1	A	424	LYS
1	A	425	LEU
1	A	428	ASP
1	A	430	THR
1	A	434	ILE
1	A	436	TRP
1	A	440	ASN
1	A	441	LEU
1	A	443	SER
1	A	444	LYS
1	A	450	ASN
1	A	452	LEU
1	A	454	ARG
1	A	455	LEU
1	A	456	PHE
1	A	458	LYS
1	A	459	SER
1	A	462	LYS
1	A	463	PRO
1	A	466	ARG
1	A	472	ILE
1	A	480	CYS
1	A	483	VAL
1	A	484	LYS
1	A	493	GLN
1	A	495	TYR
1	A	498	GLN
1	A	503	VAL
1	A	508	TYR
1	A	512	VAL

Continued on next page...

PRELIMINARY VALIDATION REPORT

Continued from previous page...

Mol	Chain	Res	Type
1	A	516	GLU
1	A	517	LEU
1	A	518	LEU
1	A	599	THR
1	A	602	THR
1	A	613	GLN
1	A	615	VAL
1	A	701	VAL
1	A	716	THR
1	A	738	CYS
1	A	747	THR
1	A	881	THR
1	A	937	SER
1	A	967	SER
2	B	34	ARG
2	B	50	SER
2	B	134	GLN
2	B	167	THR
2	B	205	SER
2	B	274	THR
2	B	331	ASN
2	B	334	ASN
2	B	356	LYS
2	B	367	VAL
2	B	376	THR
2	B	378	LYS
2	B	382	VAL
2	B	386	LYS
2	B	387	LEU
2	B	389	ASP
2	B	394	ASN
2	B	398	ASP
2	B	402	ILE
2	B	405	ASP
2	B	407	VAL
2	B	408	ARG
2	B	409	GLN
2	B	410	ILE
2	B	422	ASN
2	B	424	LYS
2	B	425	LEU
2	B	428	ASP

Continued on next page...

PRELIMINARY VALIDATION REPORT

Continued from previous page...

Mol	Chain	Res	Type
2	B	430	THR
2	B	434	ILE
2	B	436	TRP
2	B	440	ASN
2	B	441	LEU
2	B	443	SER
2	B	444	LYS
2	B	450	ASN
2	B	452	LEU
2	B	454	ARG
2	B	455	LEU
2	B	456	PHE
2	B	458	LYS
2	B	459	SER
2	B	462	LYS
2	B	463	PRO
2	B	466	ARG
2	B	472	ILE
2	B	480	CYS
2	B	483	VAL
2	B	484	LYS
2	B	493	GLN
2	B	495	TYR
2	B	498	GLN
2	B	503	VAL
2	B	508	TYR
2	B	512	VAL
2	B	517	LEU
2	B	518	LEU
2	B	531	THR
2	B	532	ASN
2	B	546	LEU
2	B	558	LYS
2	B	567	ARG
2	B	581	THR
2	B	586	ASP
2	B	615	VAL
2	B	638	THR
2	B	701	VAL
2	B	703	ASN
2	B	738	CYS
2	B	938	LEU

Continued on next page...

PRELIMINARY VALIDATION REPORT

Continued from previous page...

Mol	Chain	Res	Type
2	B	940	SER
2	B	983	ARG
2	B	1037	SER
2	B	1097	SER
3	C	33	THR
3	C	63	THR
3	C	126	VAL
3	C	167	THR
3	C	175	PHE
3	C	276	LEU
3	C	284	THR
3	C	334	ASN
3	C	335	LEU
3	C	356	LYS
3	C	367	VAL
3	C	376	THR
3	C	378	LYS
3	C	382	VAL
3	C	386	LYS
3	C	387	LEU
3	C	389	ASP
3	C	394	ASN
3	C	398	ASP
3	C	402	ILE
3	C	405	ASP
3	C	407	VAL
3	C	408	ARG
3	C	409	GLN
3	C	410	ILE
3	C	422	ASN
3	C	424	LYS
3	C	425	LEU
3	C	428	ASP
3	C	430	THR
3	C	434	ILE
3	C	436	TRP
3	C	440	ASN
3	C	441	LEU
3	C	443	SER
3	C	444	LYS
3	C	450	ASN
3	C	452	LEU

Continued on next page...

PRELIMINARY VALIDATION REPORT

Continued from previous page...

Mol	Chain	Res	Type
3	C	454	ARG
3	C	455	LEU
3	C	456	PHE
3	C	458	LYS
3	C	459	SER
3	C	462	LYS
3	C	463	PRO
3	C	466	ARG
3	C	472	ILE
3	C	480	CYS
3	C	483	VAL
3	C	484	LYS
3	C	493	GLN
3	C	495	TYR
3	C	498	GLN
3	C	503	VAL
3	C	508	TYR
3	C	512	VAL
3	C	514	SER
3	C	516	GLU
3	C	518	LEU
3	C	539	VAL
3	C	568	ASP
3	C	586	ASP
3	C	591	SER
3	C	599	THR
3	C	615	VAL
3	C	701	VAL
3	C	704	SER
3	C	705	VAL
3	C	760	CYS
3	C	785	VAL
3	C	819	GLU
3	C	856	ASN
3	C	860	VAL
3	C	881	THR
3	C	887	THR
3	C	904	TYR
3	C	974	SER
3	C	980	ILE
3	C	1077	THR
3	C	1136	THR

PRELIMINARY VALIDATION REPORT

Sometimes sidechains can be flipped to improve hydrogen bonding and reduce clashes. All (41) such sidechains are listed below:

Mol	Chain	Res	Type
1	A	218	GLN
1	A	239	GLN
1	A	280	ASN
1	A	474	GLN
1	A	532	ASN
1	A	563	GLN
1	A	580	GLN
1	A	613	GLN
1	A	751	ASN
1	A	914	ASN
1	A	1071	GLN
1	A	1101	HIS
1	A	1135	ASN
2	B	52	GLN
2	B	137	ASN
2	B	207	HIS
2	B	334	ASN
2	B	474	GLN
2	B	655	HIS
2	B	762	GLN
2	B	784	GLN
2	B	804	GLN
2	B	935	GLN
2	B	1005	GLN
2	B	1101	HIS
3	C	61	ASN
3	C	134	GLN
3	C	280	ASN
3	C	394	ASN
3	C	474	GLN
3	C	935	GLN
3	C	1005	GLN
3	C	1135	ASN
4	U	82	GLN
5	V	34	HIS
5	V	89	GLN
4	O	82	GLN
5	Q	89	GLN
4	P	82	GLN
5	S	34	HIS
5	S	89	GLN

PRELIMINARY VALIDATION REPORT

5.3.3 RNA (i)

There are no RNA molecules in this entry.

5.4 Non-standard residues in protein, DNA, RNA chains (i)

There are no non-standard protein/DNA/RNA residues in this entry.

5.5 Carbohydrates (i)

29 monosaccharides are modelled in this entry.

In the following table, the Counts columns list the number of bonds (or angles) for which Mogul statistics could be retrieved, the number of bonds (or angles) that are observed in the model and the number of bonds (or angles) that are defined in the Chemical Component Dictionary. The Link column lists molecule types, if any, to which the group is linked. The Z score for a bond length (or angle) is the number of standard deviations the observed value is removed from the expected value. A bond length (or angle) with $|Z| > 2$ is considered an outlier worth inspection. RMSZ is the root-mean-square of all Z scores of the bond lengths (or angles).

Mol	Type	Chain	Res	Link	Bond lengths			Bond angles		
					Counts	RMSZ	#|Z| > 2	Counts	RMSZ	#|Z| > 2
6	NAG	D	1	6,1	14,14,15	0.73	1 (7%)	17,19,21	2.54	6 (35%)
6	NAG	D	2	6	14,14,15	0.52	0	17,19,21	1.14	2 (11%)
6	BMA	D	3	6	11,11,12	0.44	0	15,15,17	0.49	0
6	NAG	E	1	6,2	14,14,15	0.74	1 (7%)	17,19,21	2.54	6 (35%)
6	NAG	E	2	6	14,14,15	0.49	0	17,19,21	1.12	2 (11%)
6	BMA	E	3	6	11,11,12	0.44	0	15,15,17	0.49	0
6	NAG	F	1	3,6	14,14,15	0.74	1 (7%)	17,19,21	2.53	6 (35%)
6	NAG	F	2	6	14,14,15	0.50	0	17,19,21	1.12	2 (11%)
6	BMA	F	3	6	11,11,12	0.43	0	15,15,17	0.48	0
7	NAG	G	1	7	14,14,15	0.75	1 (7%)	17,19,21	0.61	0
7	NAG	G	2	7	14,14,15	0.42	0	17,19,21	1.23	1 (5%)
7	NAG	H	1	7,1	14,14,15	0.38	0	17,19,21	0.53	0
7	NAG	H	2	7	14,14,15	0.33	0	17,19,21	0.38	0
7	NAG	I	1	7,1	14,14,15	0.28	0	17,19,21	0.62	1 (5%)
7	NAG	I	2	7	14,14,15	0.29	0	17,19,21	0.37	0
7	NAG	J	1	7,1	14,14,15	1.03	1 (7%)	17,19,21	0.92	1 (5%)
7	NAG	J	2	7	14,14,15	0.26	0	17,19,21	0.41	0
7	NAG	K	1	7,1	14,14,15	0.38	0	17,19,21	1.27	2 (11%)
7	NAG	K	2	7	14,14,15	0.40	0	17,19,21	1.27	1 (5%)
7	NAG	L	1	7,2	14,14,15	0.49	0	17,19,21	0.44	0

PRELIMINARY VALIDATION REPORT

Mol	Type	Chain	Res	Link	Bond lengths			Bond angles		
					Counts	RMSZ	#|Z| > 2	Counts	RMSZ	#|Z| > 2
7	NAG	L	2	7	14,14,15	0.22	0	17,19,21	0.43	0
7	NAG	M	1	7,2	14,14,15	0.78	1 (7%)	17,19,21	0.78	1 (5%)
7	NAG	M	2	7	14,14,15	0.75	1 (7%)	17,19,21	0.71	0
7	NAG	N	1	3,7	14,14,15	0.33	0	17,19,21	0.38	0
7	NAG	N	2	7	14,14,15	0.18	0	17,19,21	0.50	0
7	NAG	R	1	3,7	14,14,15	0.73	1 (7%)	17,19,21	0.68	0
7	NAG	R	2	7	14,14,15	0.20	0	17,19,21	0.42	0
7	NAG	T	1	3,7	14,14,15	0.51	0	17,19,21	0.50	0
7	NAG	T	2	7	14,14,15	0.21	0	17,19,21	0.47	0

In the following table, the Chirals column lists the number of chiral outliers, the number of chiral centers analysed, the number of these observed in the model and the number defined in the Chemical Component Dictionary. Similar counts are reported in the Torsion and Rings columns. '-' means no outliers of that kind were identified.

Mol	Type	Chain	Res	Link	Chirals	Torsions	Rings
6	NAG	D	1	6,1	-	3/6/23/26	0/1/1/1
6	NAG	D	2	6	-	6/6/23/26	0/1/1/1
6	BMA	D	3	6	-	0/2/19/22	0/1/1/1
6	NAG	E	1	6,2	-	3/6/23/26	0/1/1/1
6	NAG	E	2	6	-	6/6/23/26	0/1/1/1
6	BMA	E	3	6	-	0/2/19/22	0/1/1/1
6	NAG	F	1	3,6	-	3/6/23/26	0/1/1/1
6	NAG	F	2	6	-	6/6/23/26	0/1/1/1
6	BMA	F	3	6	-	0/2/19/22	0/1/1/1
7	NAG	G	1	7	-	0/6/23/26	0/1/1/1
7	NAG	G	2	7	-	5/6/23/26	0/1/1/1
7	NAG	H	1	7,1	-	0/6/23/26	0/1/1/1
7	NAG	H	2	7	-	1/6/23/26	0/1/1/1
7	NAG	I	1	7,1	-	0/6/23/26	0/1/1/1
7	NAG	I	2	7	-	1/6/23/26	0/1/1/1
7	NAG	J	1	7,1	-	3/6/23/26	0/1/1/1
7	NAG	J	2	7	-	2/6/23/26	0/1/1/1
7	NAG	K	1	7,1	-	4/6/23/26	0/1/1/1
7	NAG	K	2	7	-	5/6/23/26	0/1/1/1
7	NAG	L	1	7,2	-	1/6/23/26	0/1/1/1
7	NAG	L	2	7	-	0/6/23/26	0/1/1/1
7	NAG	M	1	7,2	-	2/6/23/26	0/1/1/1
7	NAG	M	2	7	-	3/6/23/26	0/1/1/1

Continued on next page...

PRELIMINARY VALIDATION REPORT

Continued from previous page...

Mol	Type	Chain	Res	Link	Chirals	Torsions	Rings
7	NAG	N	1	3,7	-	2/6/23/26	0/1/1/1
7	NAG	N	2	7	-	2/6/23/26	0/1/1/1
7	NAG	R	1	3,7	-	2/6/23/26	0/1/1/1
7	NAG	R	2	7	-	2/6/23/26	0/1/1/1
7	NAG	T	1	3,7	-	2/6/23/26	0/1/1/1
7	NAG	T	2	7	-	1/6/23/26	0/1/1/1

All (8) bond length outliers are listed below:

Mol	Chain	Res	Type	Atoms	Z	Observed(Å)	Ideal(Å)
7	J	1	NAG	O5-C1	-3.68	1.37	1.43
7	M	1	NAG	O5-C1	-2.71	1.39	1.43
7	R	1	NAG	O5-C1	-2.68	1.39	1.43
7	G	1	NAG	O5-C1	-2.62	1.39	1.43
6	E	1	NAG	O5-C1	-2.28	1.40	1.43
6	F	1	NAG	O5-C1	-2.28	1.40	1.43
6	D	1	NAG	O5-C1	-2.23	1.40	1.43
7	M	2	NAG	O5-C1	-2.04	1.40	1.43

All (31) bond angle outliers are listed below:

Mol	Chain	Res	Type	Atoms	Z	Observed(^{o})	Ideal(^{o})
6	D	1	NAG	C1-O5-C5	-7.33	102.27	112.19
6	E	1	NAG	C1-O5-C5	-7.31	102.28	112.19
6	F	1	NAG	C1-O5-C5	-7.30	102.30	112.19
7	K	2	NAG	C2-N2-C7	4.33	129.06	122.90
7	K	1	NAG	C2-N2-C7	4.32	129.06	122.90
7	G	2	NAG	C2-N2-C7	4.23	128.93	122.90
6	E	1	NAG	C6-C5-C4	3.30	120.73	113.00
6	F	1	NAG	C6-C5-C4	3.29	120.71	113.00
6	D	1	NAG	C6-C5-C4	3.29	120.70	113.00
6	D	1	NAG	O5-C5-C4	-3.27	102.86	110.83
6	F	1	NAG	O5-C5-C4	-3.27	102.87	110.83
6	E	1	NAG	O5-C5-C4	-3.26	102.89	110.83
6	D	2	NAG	C1-O5-C5	3.06	116.34	112.19
6	D	1	NAG	C4-C3-C2	3.03	115.47	111.02
6	E	1	NAG	C4-C3-C2	3.03	115.45	111.02
6	F	1	NAG	C4-C3-C2	3.02	115.44	111.02
6	F	2	NAG	C1-O5-C5	3.01	116.27	112.19
6	E	2	NAG	C1-O5-C5	2.99	116.24	112.19
6	E	1	NAG	C2-N2-C7	2.72	126.77	122.90

Continued on next page...

PRELIMINARY VALIDATION REPORT

Continued from previous page...

Mol	Chain	Res	Type	Atoms	Z	Observed(°)	Ideal(°)
6	D	1	NAG	C2-N2-C7	2.69	126.73	122.90
6	F	1	NAG	C2-N2-C7	2.68	126.72	122.90
7	J	1	NAG	C3-C4-C5	2.26	114.28	110.24
6	D	2	NAG	C3-C4-C5	2.24	114.23	110.24
6	E	1	NAG	O4-C4-C5	2.23	114.85	109.30
6	F	1	NAG	O4-C4-C5	2.23	114.83	109.30
6	D	1	NAG	O4-C4-C5	2.23	114.83	109.30
6	E	2	NAG	C3-C4-C5	2.20	114.17	110.24
6	F	2	NAG	C3-C4-C5	2.20	114.17	110.24
7	I	1	NAG	C1-O5-C5	2.12	115.07	112.19
7	K	1	NAG	C1-C2-N2	2.06	114.00	110.49
7	M	1	NAG	C1-O5-C5	2.02	114.92	112.19

There are no chirality outliers.

All (65) torsion outliers are listed below:

Mol	Chain	Res	Type	Atoms
6	D	1	NAG	C8-C7-N2-C2
6	D	1	NAG	O7-C7-N2-C2
6	D	2	NAG	C8-C7-N2-C2
6	D	2	NAG	O7-C7-N2-C2
6	E	1	NAG	C8-C7-N2-C2
6	E	1	NAG	O7-C7-N2-C2
6	E	2	NAG	C8-C7-N2-C2
6	E	2	NAG	O7-C7-N2-C2
6	F	1	NAG	C8-C7-N2-C2
6	F	1	NAG	O7-C7-N2-C2
6	F	2	NAG	C8-C7-N2-C2
6	F	2	NAG	O7-C7-N2-C2
7	M	2	NAG	C4-C5-C6-O6
7	M	1	NAG	O5-C5-C6-O6
7	M	2	NAG	O5-C5-C6-O6
7	J	1	NAG	O5-C5-C6-O6
7	J	2	NAG	O5-C5-C6-O6
7	R	1	NAG	O5-C5-C6-O6
7	M	1	NAG	C4-C5-C6-O6
7	G	2	NAG	O5-C5-C6-O6
7	T	1	NAG	O5-C5-C6-O6
7	K	2	NAG	O5-C5-C6-O6
7	J	1	NAG	C4-C5-C6-O6
7	J	2	NAG	C4-C5-C6-O6
7	T	1	NAG	C4-C5-C6-O6

Continued on next page...

PRELIMINARY VALIDATION REPORT

Continued from previous page...

Mol	Chain	Res	Type	Atoms
7	G	2	NAG	C8-C7-N2-C2
7	G	2	NAG	O7-C7-N2-C2
7	K	1	NAG	C8-C7-N2-C2
7	K	1	NAG	O7-C7-N2-C2
7	K	2	NAG	C8-C7-N2-C2
7	K	2	NAG	O7-C7-N2-C2
7	K	2	NAG	C4-C5-C6-O6
7	R	1	NAG	C4-C5-C6-O6
6	E	2	NAG	O5-C5-C6-O6
6	F	2	NAG	O5-C5-C6-O6
6	D	2	NAG	O5-C5-C6-O6
7	N	2	NAG	O5-C5-C6-O6
7	N	2	NAG	C4-C5-C6-O6
6	D	2	NAG	C1-C2-N2-C7
6	E	2	NAG	C1-C2-N2-C7
6	F	2	NAG	C1-C2-N2-C7
7	T	2	NAG	O5-C5-C6-O6
7	H	2	NAG	O5-C5-C6-O6
7	G	2	NAG	C4-C5-C6-O6
6	D	1	NAG	C3-C2-N2-C7
6	E	1	NAG	C3-C2-N2-C7
6	F	1	NAG	C3-C2-N2-C7
7	N	1	NAG	C4-C5-C6-O6
7	J	1	NAG	C1-C2-N2-C7
7	R	2	NAG	C4-C5-C6-O6
7	N	1	NAG	O5-C5-C6-O6
6	D	2	NAG	C3-C2-N2-C7
6	E	2	NAG	C3-C2-N2-C7
6	F	2	NAG	C3-C2-N2-C7
7	M	2	NAG	C3-C2-N2-C7
6	D	2	NAG	C4-C5-C6-O6
6	E	2	NAG	C4-C5-C6-O6
6	F	2	NAG	C4-C5-C6-O6
7	L	1	NAG	C1-C2-N2-C7
7	R	2	NAG	O5-C5-C6-O6
7	K	1	NAG	C4-C5-C6-O6
7	G	2	NAG	C3-C2-N2-C7
7	K	1	NAG	C3-C2-N2-C7
7	K	2	NAG	C3-C2-N2-C7
7	I	2	NAG	C4-C5-C6-O6

There are no ring outliers.

PRELIMINARY VALIDATION REPORT

13 monomers are involved in 9 short contacts:

Mol	Chain	Res	Type	Clashes	Symm-Clashes
6	F	2	NAG	1	0
6	D	1	NAG	1	0
6	E	2	NAG	1	0
6	D	2	NAG	1	0
7	L	1	NAG	1	0
7	M	1	NAG	1	0
6	E	1	NAG	1	0
7	M	2	NAG	2	0
7	K	2	NAG	1	0
6	F	1	NAG	1	0
7	L	2	NAG	1	0
7	K	1	NAG	1	0
7	G	2	NAG	1	0

The following is a two-dimensional graphical depiction of Mogul quality analysis of bond lengths, bond angles, torsion angles, and ring geometry for oligosaccharide.

Oligosaccharide Chain D

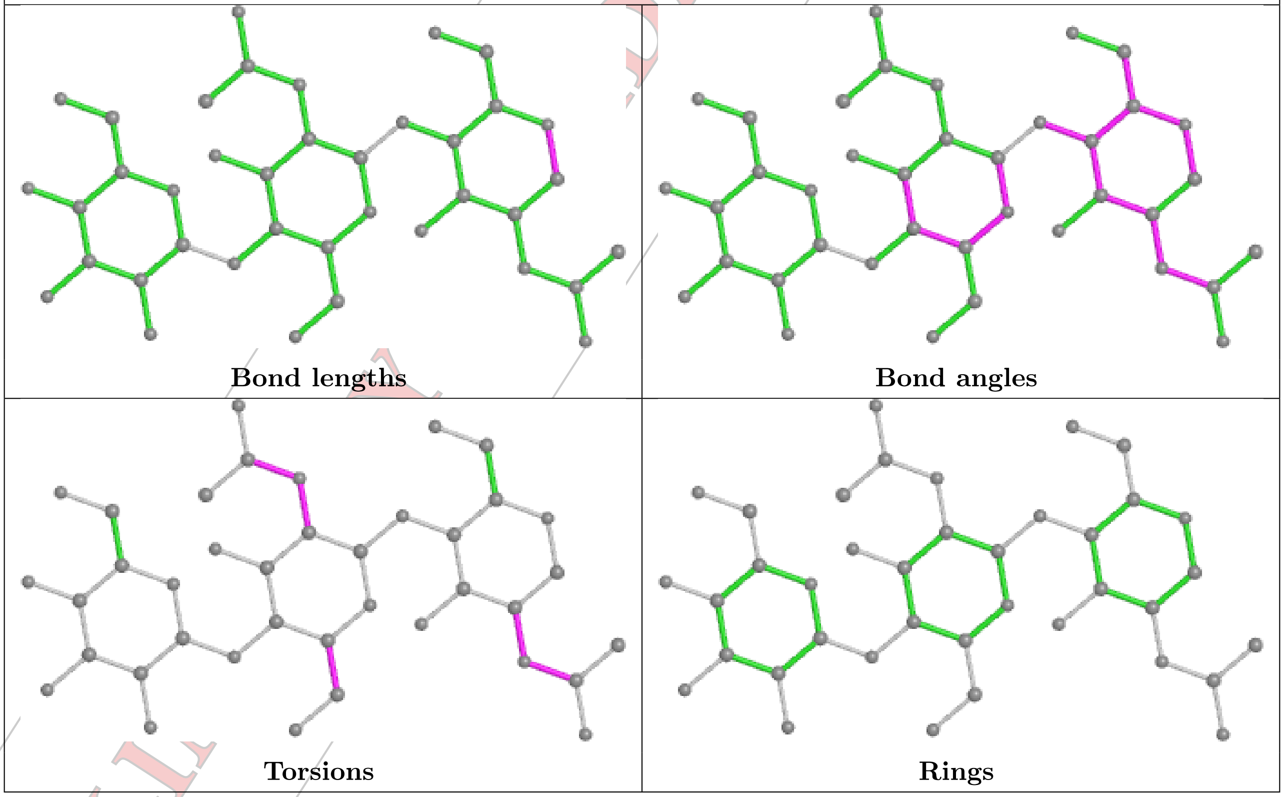

PRELIMINARY VALIDATION REPORT

Oligosaccharide Chain E

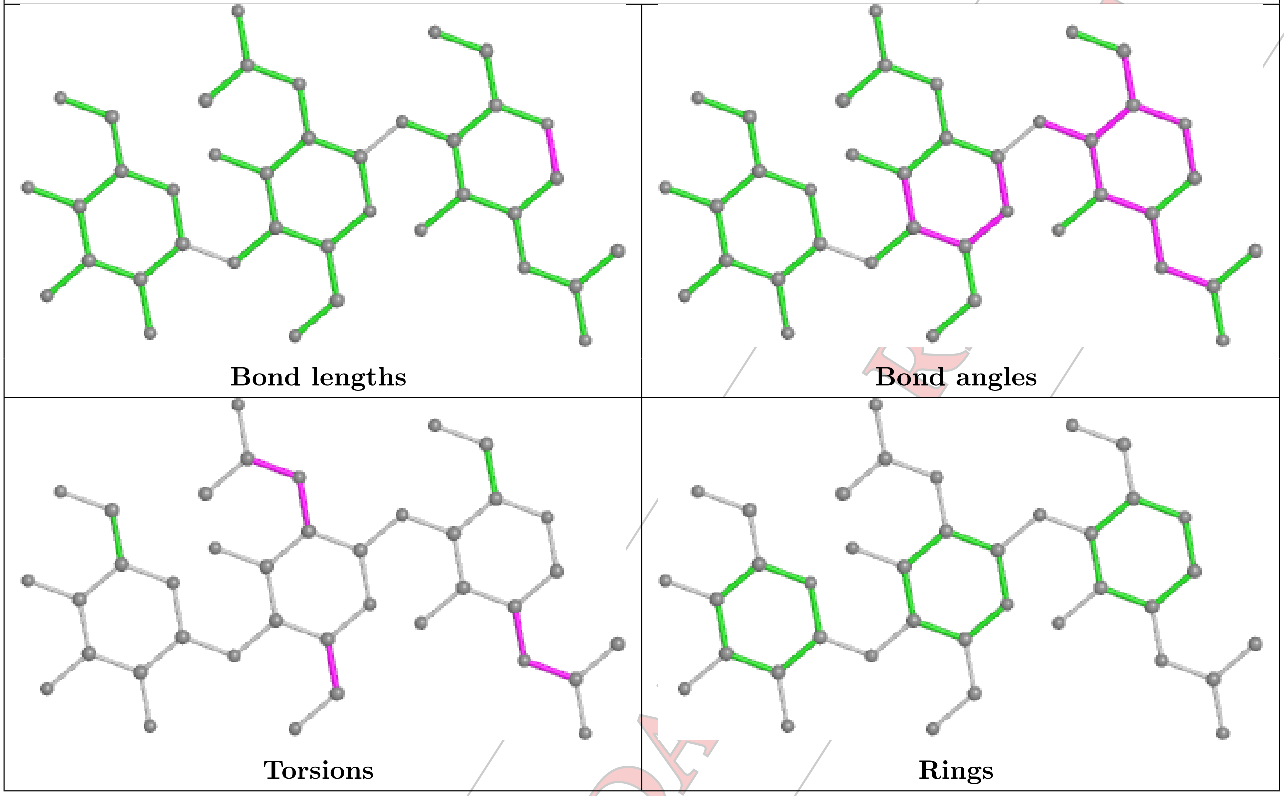

Oligosaccharide Chain F

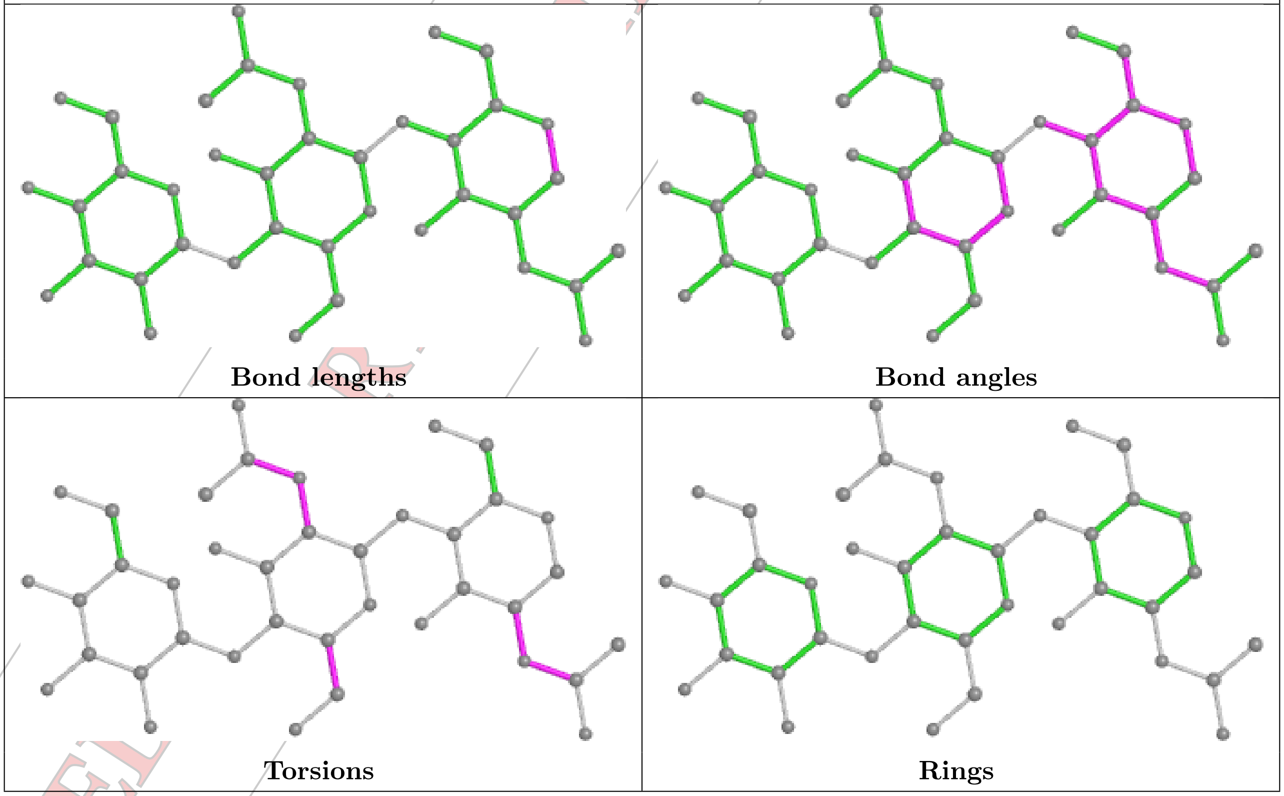

Oligosaccharide Chain G

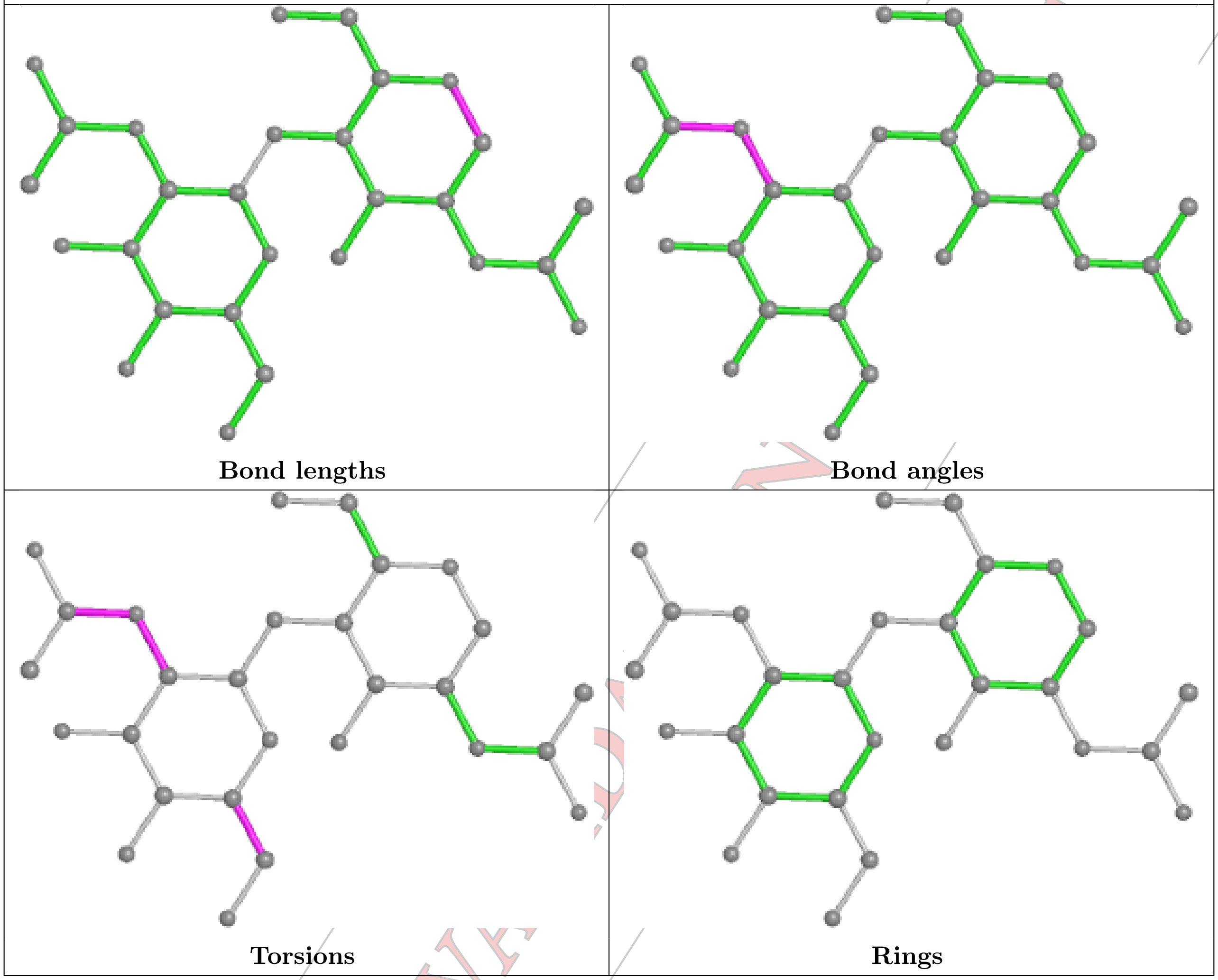

PRELIMINARY VALIDATION

Oligosaccharide Chain H	
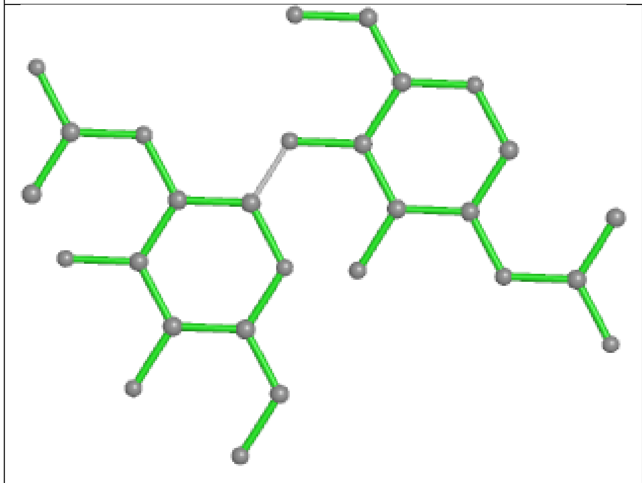 Bond lengths	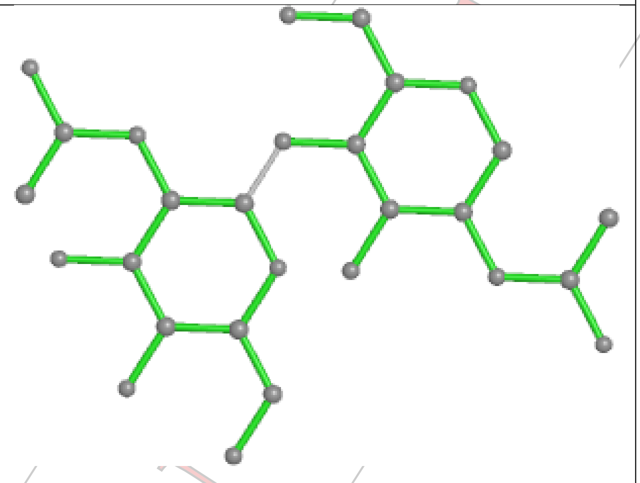 Bond angles
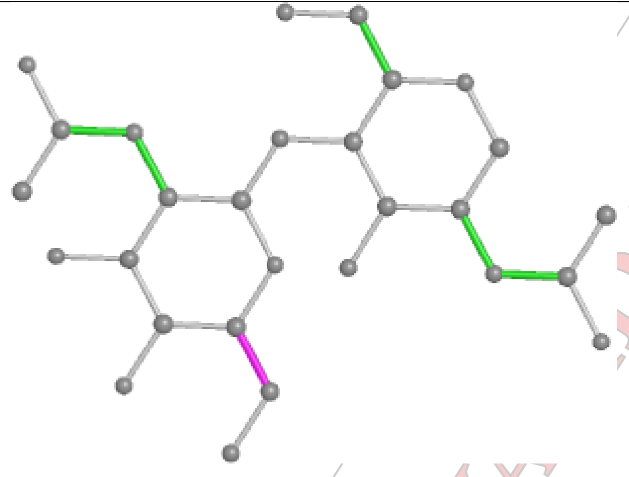 Torsions	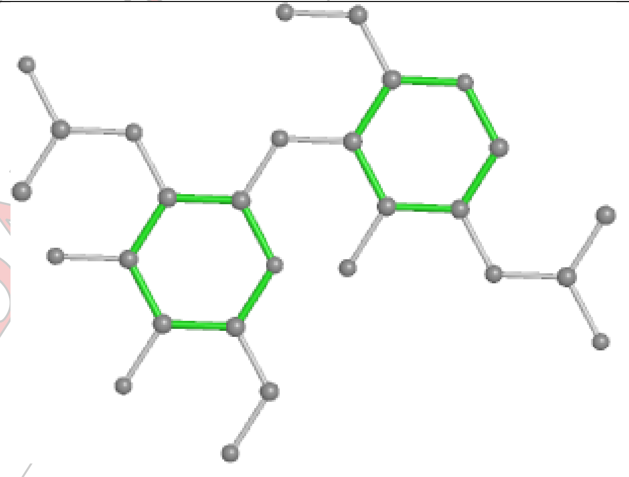 Rings

PRELIMINARY VALIDATION

Oligosaccharide Chain I	
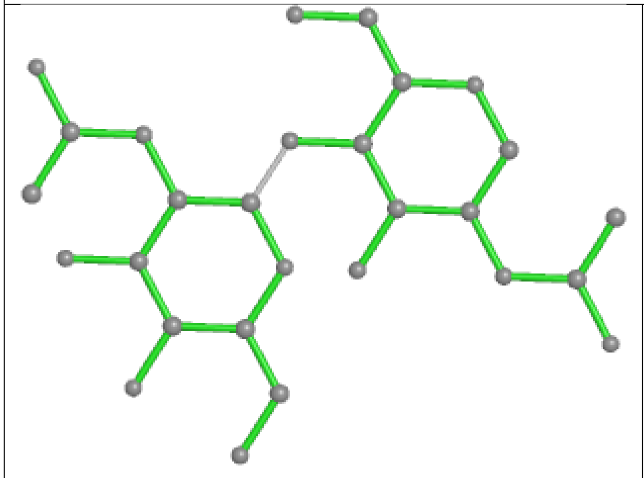 Bond lengths	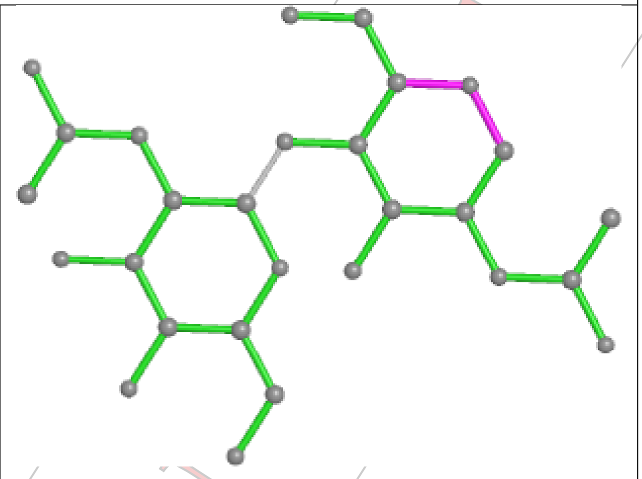 Bond angles
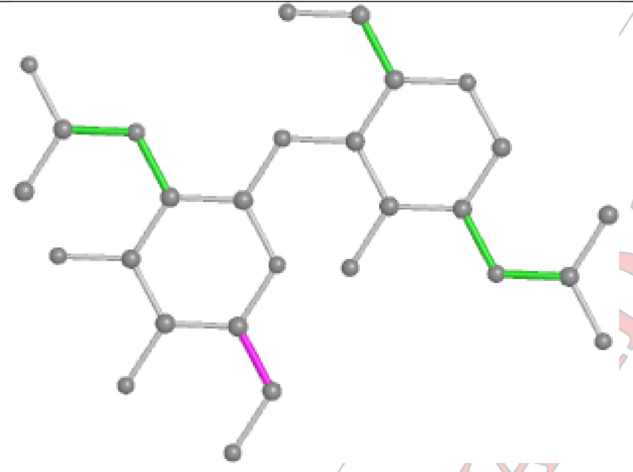 Torsions	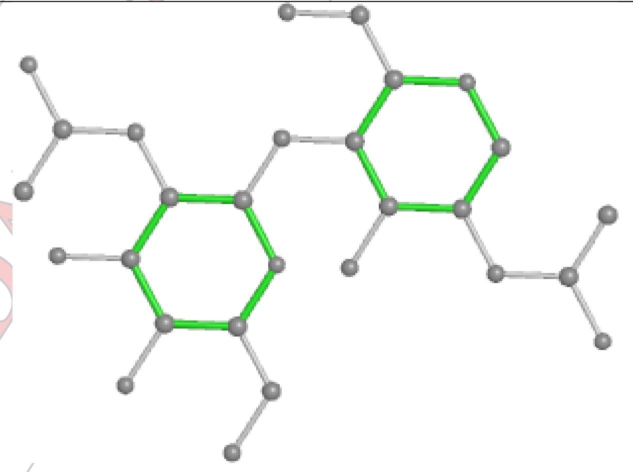 Rings

PRELIMINARY VALIDATION

Oligosaccharide Chain J

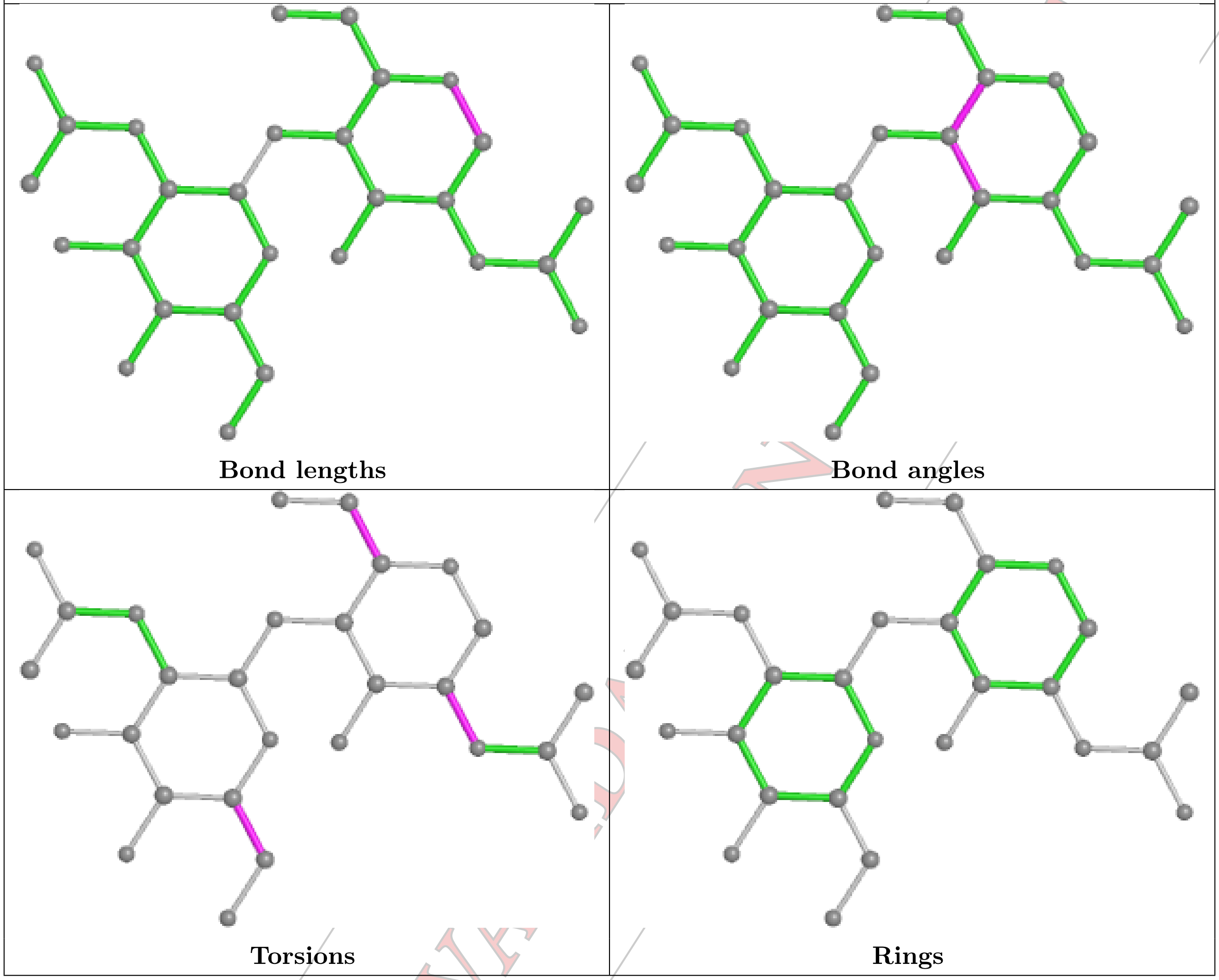

PRELIMINARY VALIDATION

Oligosaccharide Chain K

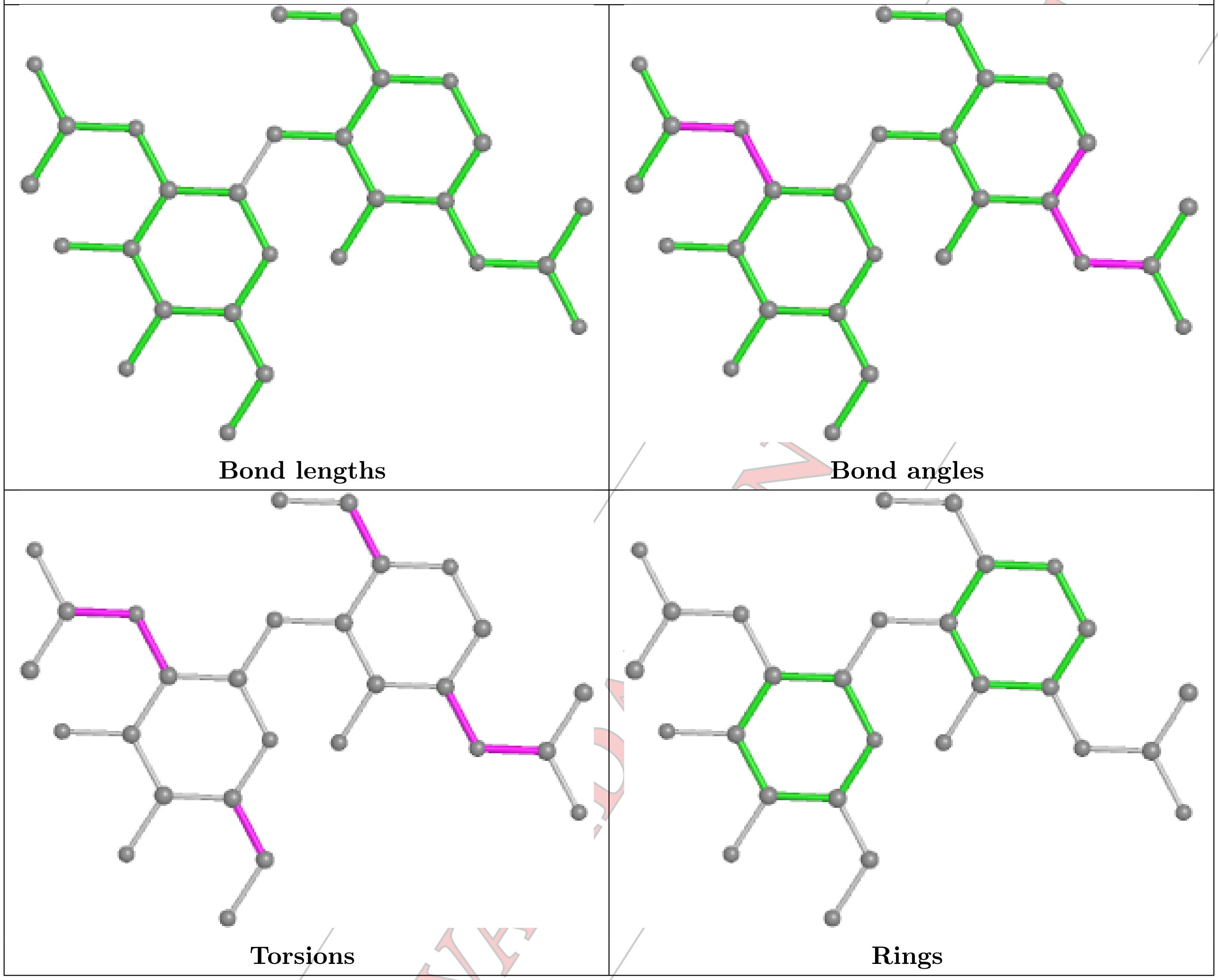

Oligosaccharide Chain L

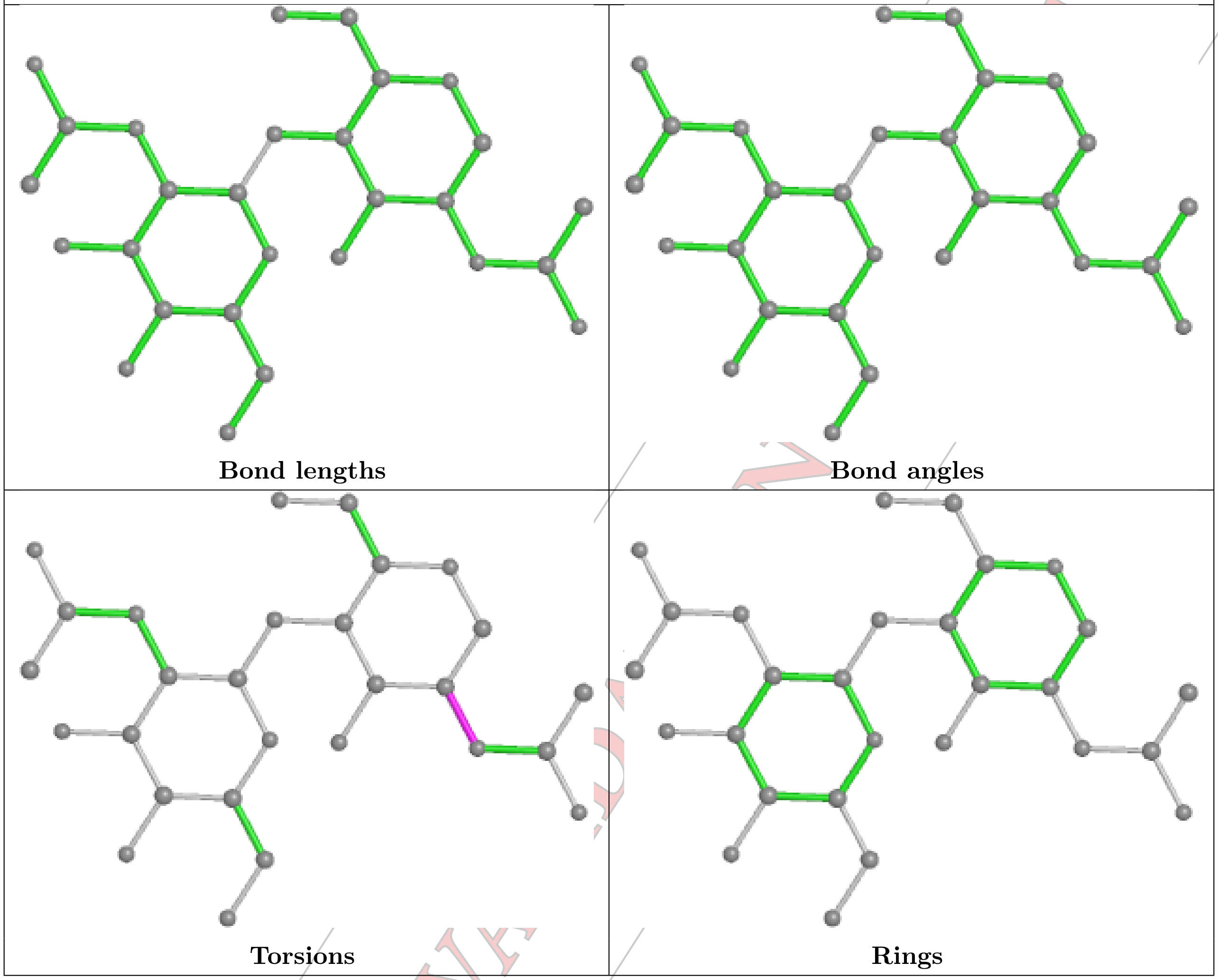

PRELIMINARY VALIDATION

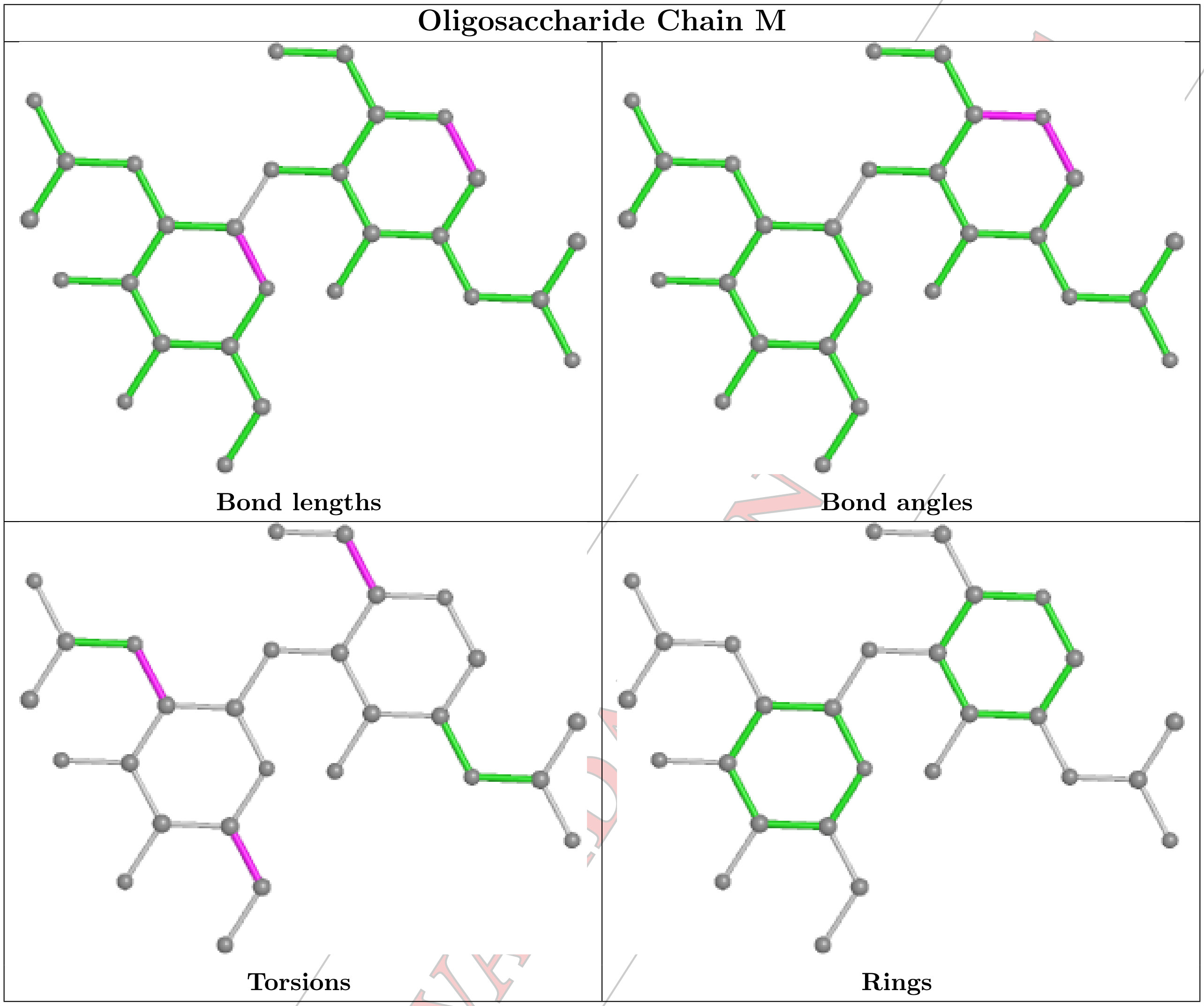

PRELIMINARY VALIDATION

Oligosaccharide Chain N

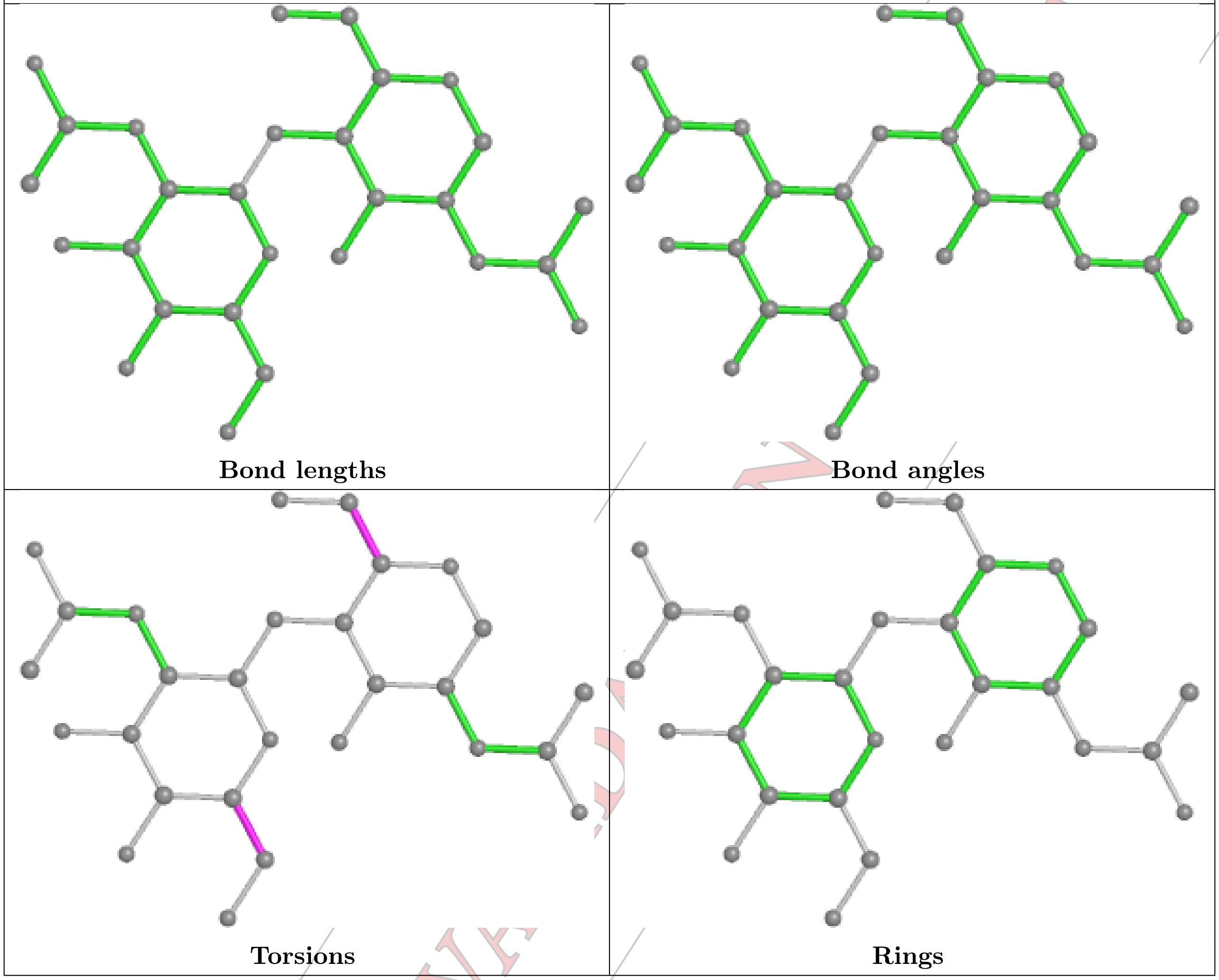

PRELIMINARY

Oligosaccharide Chain R

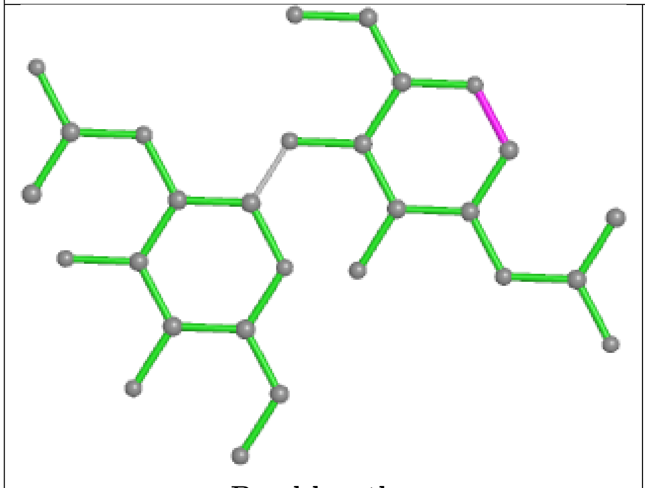

Bond lengths

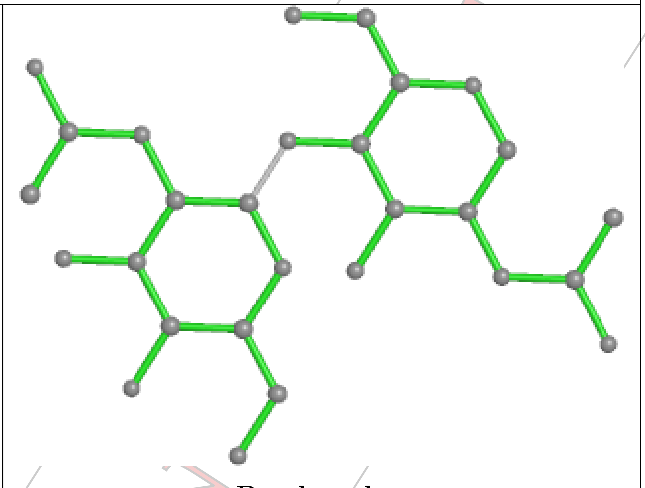

Bond angles

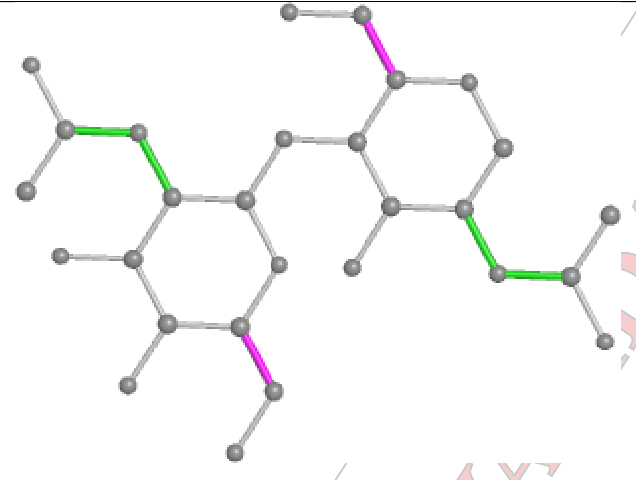

Torsions

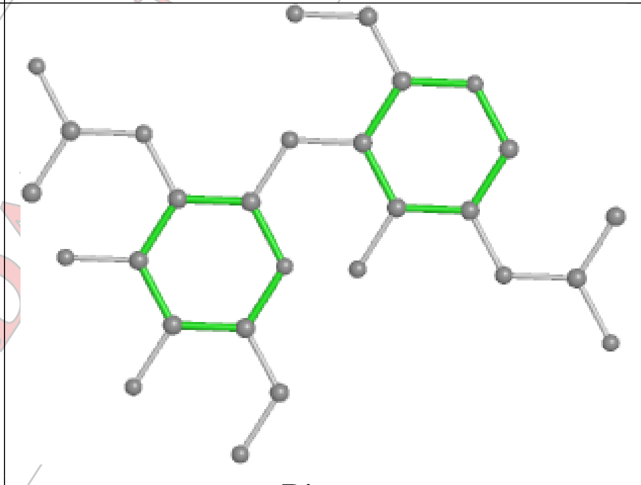

Rings

PRELIMINARY

Oligosaccharide Chain T

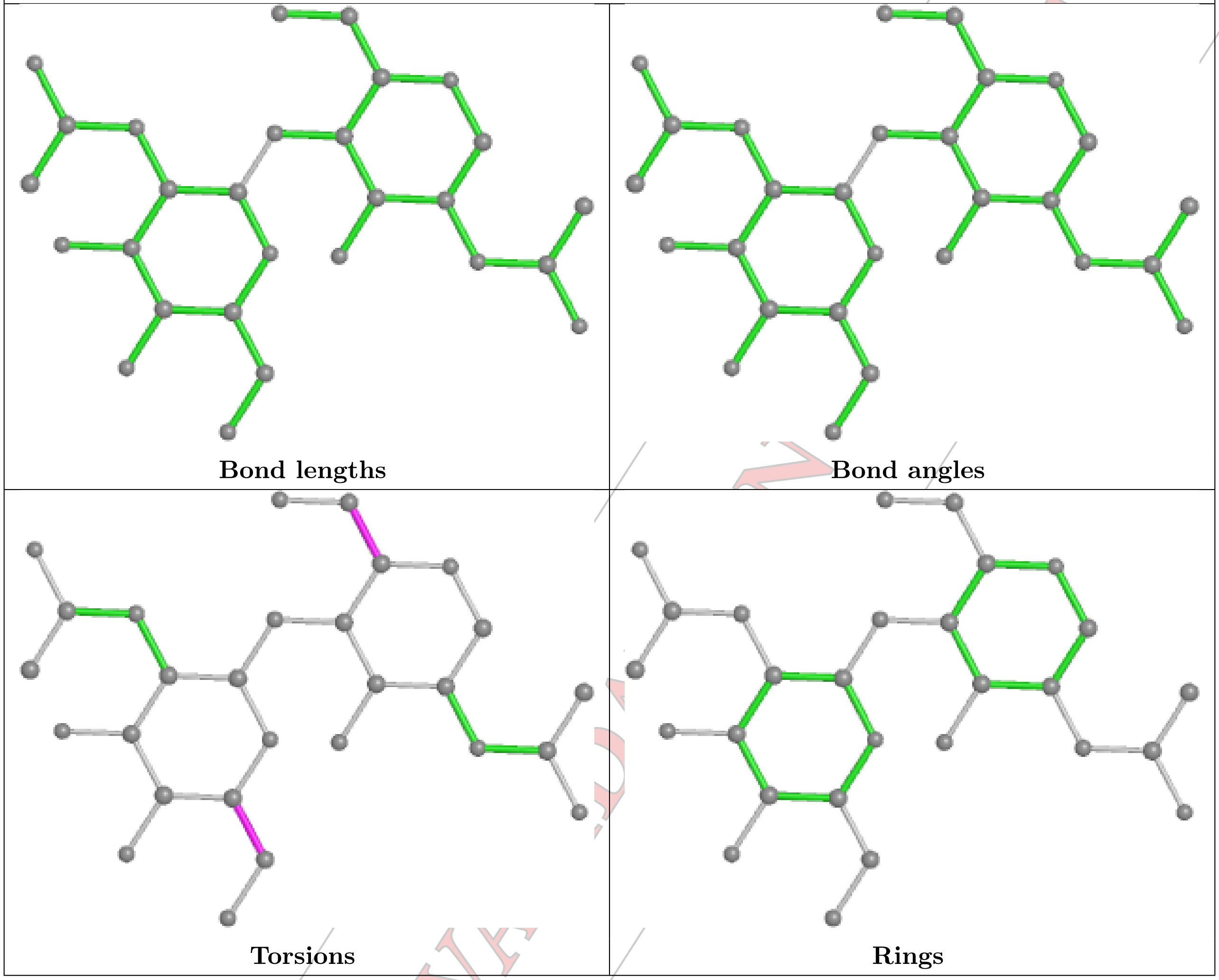

5.6 Ligand geometry (i)

26 ligands are modelled in this entry.

In the following table, the Counts columns list the number of bonds (or angles) for which Mogul statistics could be retrieved, the number of bonds (or angles) that are observed in the model and the number of bonds (or angles) that are defined in the Chemical Component Dictionary. The Link column lists molecule types, if any, to which the group is linked. The Z score for a bond length (or angle) is the number of standard deviations the observed value is removed from the expected value. A bond length (or angle) with $|Z| > 2$ is considered an outlier worth inspection. RMSZ is the root-mean-square of all Z scores of the bond lengths (or angles).

Mol	Type	Chain	Res	Link	Bond lengths			Bond angles		
					Counts	RMSZ	$\#\|Z\| > 2$	Counts	RMSZ	$\#\|Z\| > 2$
8	NAG	A	1304	1	14,14,15	0.34	0	17,19,21	0.57	0
8	NAG	B	1302	2	14,14,15	0.27	0	17,19,21	0.54	0
8	NAG	B	1303	2	14,14,15	0.19	0	17,19,21	0.51	0
8	NAG	C	1308	3	14,14,15	0.26	0	17,19,21	0.43	0

Mol	Type	Chain	Res	Link	Bond lengths			Bond angles		
					Counts	RMSZ	#|Z| > 2	Counts	RMSZ	#|Z| > 2
8	NAG	C	1306	3	14,14,15	0.18	0	17,19,21	0.47	0
8	NAG	B	1307	2	14,14,15	0.24	0	17,19,21	0.42	0
8	NAG	B	1304	2	14,14,15	0.33	0	17,19,21	0.54	0
8	NAG	C	1307	3	14,14,15	0.39	0	17,19,21	0.38	0
8	NAG	C	1302	3	14,14,15	0.19	0	17,19,21	0.45	0
8	NAG	C	1304	3	14,14,15	0.19	0	17,19,21	0.43	0
8	NAG	C	1305	3	14,14,15	0.21	0	17,19,21	0.42	0
8	NAG	B	1309	2	14,14,15	0.38	0	17,19,21	0.41	0
8	NAG	A	1305	1	14,14,15	0.26	0	17,19,21	0.48	0
8	NAG	B	1310	2	14,14,15	0.32	0	17,19,21	0.70	1 (5%)
8	NAG	A	1302	1	14,14,15	0.51	0	17,19,21	0.62	0
8	NAG	B	1306	2	14,14,15	0.21	0	17,19,21	0.49	0
8	NAG	B	1312	2	14,14,15	0.19	0	17,19,21	0.71	1 (5%)
8	NAG	C	1309	3	14,14,15	0.15	0	17,19,21	0.57	0
8	NAG	A	1307	1	14,14,15	0.22	0	17,19,21	0.56	0
8	NAG	C	1301	3	14,14,15	0.22	0	17,19,21	0.42	0
8	NAG	A	1306	1	14,14,15	0.34	0	17,19,21	1.27	1 (5%)
8	NAG	A	1303	1	14,14,15	0.23	0	17,19,21	0.44	0
8	NAG	B	1308	2	14,14,15	0.18	0	17,19,21	0.49	0
8	NAG	A	1301	1	14,14,15	0.31	0	17,19,21	0.43	0
8	NAG	B	1311	2	14,14,15	0.31	0	17,19,21	0.39	0
8	NAG	B	1301	2	14,14,15	0.25	0	17,19,21	0.53	0

In the following table, the Chirals column lists the number of chiral outliers, the number of chiral centers analysed, the number of these observed in the model and the number defined in the Chemical Component Dictionary. Similar counts are reported in the Torsion and Rings columns. '-' means no outliers of that kind were identified.

Mol	Type	Chain	Res	Link	Chirals	Torsions	Rings
8	NAG	A	1304	1	-	4/6/23/26	0/1/1/1
8	NAG	B	1302	2	-	3/6/23/26	0/1/1/1
8	NAG	B	1303	2	-	4/6/23/26	0/1/1/1
8	NAG	C	1308	3	-	0/6/23/26	0/1/1/1
8	NAG	C	1306	3	-	2/6/23/26	0/1/1/1
8	NAG	B	1307	2	-	0/6/23/26	0/1/1/1
8	NAG	B	1304	2	-	3/6/23/26	0/1/1/1
8	NAG	C	1307	3	-	2/6/23/26	0/1/1/1
8	NAG	C	1302	3	-	4/6/23/26	0/1/1/1
8	NAG	C	1304	3	-	2/6/23/26	0/1/1/1
8	NAG	C	1305	3	-	0/6/23/26	0/1/1/1

Continued on next page...

PRELIMINARY VALIDATION REPORT

Continued from previous page...

Mol	Type	Chain	Res	Link	Chirals	Torsions	Rings
8	NAG	B	1309	2	-	2/6/23/26	0/1/1/1
8	NAG	A	1305	1	-	2/6/23/26	0/1/1/1
8	NAG	B	1310	2	-	1/6/23/26	0/1/1/1
8	NAG	A	1302	1	-	3/6/23/26	0/1/1/1
8	NAG	B	1306	2	-	3/6/23/26	0/1/1/1
8	NAG	B	1312	2	-	2/6/23/26	0/1/1/1
8	NAG	C	1309	3	-	2/6/23/26	0/1/1/1
8	NAG	A	1307	1	-	2/6/23/26	0/1/1/1
8	NAG	C	1301	3	-	2/6/23/26	0/1/1/1
8	NAG	A	1306	1	-	3/6/23/26	0/1/1/1
8	NAG	A	1303	1	-	4/6/23/26	0/1/1/1
8	NAG	B	1308	2	-	2/6/23/26	0/1/1/1
8	NAG	A	1301	1	-	3/6/23/26	0/1/1/1
8	NAG	B	1311	2	-	2/6/23/26	0/1/1/1
8	NAG	B	1301	2	-	4/6/23/26	0/1/1/1

There are no bond length outliers.

All (3) bond angle outliers are listed below:

Mol	Chain	Res	Type	Atoms	Z	Observed(^{o})	Ideal(^{o})
8	A	1306	NAG	C2-N2-C7	4.31	129.04	122.90
8	B	1312	NAG	C1-O5-C5	2.58	115.69	112.19
8	B	1310	NAG	C1-O5-C5	2.39	115.43	112.19

There are no chirality outliers.

All (61) torsion outliers are listed below:

Mol	Chain	Res	Type	Atoms
8	B	1304	NAG	C4-C5-C6-O6
8	C	1301	NAG	C4-C5-C6-O6
8	C	1306	NAG	C4-C5-C6-O6
8	A	1301	NAG	O5-C5-C6-O6
8	A	1303	NAG	O5-C5-C6-O6
8	A	1305	NAG	O5-C5-C6-O6
8	C	1306	NAG	O5-C5-C6-O6
8	B	1304	NAG	O5-C5-C6-O6
8	C	1301	NAG	O5-C5-C6-O6
8	A	1307	NAG	C4-C5-C6-O6

Continued on next page...

PRELIMINARY VALIDATION REPORT

Continued from previous page...

Mol	Chain	Res	Type	Atoms
8	B	1302	NAG	C4-C5-C6-O6
8	B	1306	NAG	O5-C5-C6-O6
8	B	1312	NAG	O5-C5-C6-O6
8	A	1304	NAG	O5-C5-C6-O6
8	A	1301	NAG	C4-C5-C6-O6
8	B	1301	NAG	O5-C5-C6-O6
8	B	1303	NAG	O5-C5-C6-O6
8	C	1309	NAG	O5-C5-C6-O6
8	A	1304	NAG	C4-C5-C6-O6
8	A	1302	NAG	C4-C5-C6-O6
8	A	1307	NAG	O5-C5-C6-O6
8	B	1311	NAG	O5-C5-C6-O6
8	B	1301	NAG	C4-C5-C6-O6
8	C	1309	NAG	C4-C5-C6-O6
8	A	1303	NAG	C4-C5-C6-O6
8	A	1305	NAG	C4-C5-C6-O6
8	C	1307	NAG	C4-C5-C6-O6
8	B	1303	NAG	C4-C5-C6-O6
8	B	1306	NAG	C4-C5-C6-O6
8	B	1312	NAG	C4-C5-C6-O6
8	A	1303	NAG	C8-C7-N2-C2
8	A	1303	NAG	O7-C7-N2-C2
8	A	1304	NAG	C8-C7-N2-C2
8	A	1304	NAG	O7-C7-N2-C2
8	A	1306	NAG	C8-C7-N2-C2
8	A	1306	NAG	O7-C7-N2-C2
8	B	1301	NAG	C8-C7-N2-C2
8	B	1301	NAG	O7-C7-N2-C2
8	B	1303	NAG	C8-C7-N2-C2
8	B	1303	NAG	O7-C7-N2-C2
8	C	1302	NAG	C8-C7-N2-C2
8	C	1302	NAG	O7-C7-N2-C2
8	B	1302	NAG	O5-C5-C6-O6
8	C	1307	NAG	O5-C5-C6-O6
8	B	1309	NAG	O5-C5-C6-O6
8	A	1302	NAG	O5-C5-C6-O6
8	C	1302	NAG	C4-C5-C6-O6
8	B	1311	NAG	C4-C5-C6-O6
8	B	1310	NAG	O5-C5-C6-O6
8	C	1302	NAG	O5-C5-C6-O6
8	B	1308	NAG	C4-C5-C6-O6
8	B	1304	NAG	C1-C2-N2-C7

Continued on next page...

PRELIMINARY VALIDATION REPORT

Continued from previous page...

Mol	Chain	Res	Type	Atoms
8	B	1308	NAG	O5-C5-C6-O6
8	B	1309	NAG	C4-C5-C6-O6
8	A	1302	NAG	C3-C2-N2-C7
8	B	1302	NAG	C3-C2-N2-C7
8	B	1306	NAG	C3-C2-N2-C7
8	C	1304	NAG	C4-C5-C6-O6
8	A	1301	NAG	C1-C2-N2-C7
8	C	1304	NAG	O5-C5-C6-O6
8	A	1306	NAG	C3-C2-N2-C7

There are no ring outliers.

4 monomers are involved in 6 short contacts:

Mol	Chain	Res	Type	Clashes	Symm-Clashes
8	A	1302	NAG	2	0
8	C	1301	NAG	2	0
8	A	1306	NAG	1	0
8	B	1301	NAG	1	0

5.7 Other polymers (i)

There are no such residues in this entry.

5.8 Polymer linkage issues (i)

The following chains have linkage breaks:

Mol	Chain	Number of breaks
3	C	8
1	A	8
2	B	8

All chain breaks are listed below:

Model	Chain	Residue-1	Atom-1	Residue-2	Atom-2	Distance (Å)
1	C	241:LEU	C	261:GLY	N	14.44
1	A	241:LEU	C	261:GLY	N	13.72
1	C	620:VAL	C	641:ASN	N	12.86
1	A	620:VAL	C	640:SER	N	12.55
1	C	176:LEU	C	187:LYS	N	12.05

Continued on next page...

PRELIMINARY VALIDATION REPORT

Continued from previous page...

Model	Chain	Residue-1	Atom-1	Residue-2	Atom-2	Distance (Å)
1	A	176:LEU	C	187:LYS	N	11.99
1	B	176:LEU	C	187:LYS	N	11.91
1	A	828:LEU	C	853:GLN	N	11.67
1	C	828:LEU	C	853:GLN	N	10.51
1	B	620:VAL	C	638:THR	N	9.78
1	B	241:LEU	C	263:ALA	N	9.47
1	B	209:PRO	C	217:PRO	N	9.42
1	B	828:LEU	C	853:GLN	N	9.11
1	C	95:THR	C	99:ASN	N	8.82
1	A	95:THR	C	99:ASN	N	7.64
1	B	66:HIS	C	81:ASN	N	7.50
1	A	142:GLY	C	156:GLU	N	7.26
1	C	66:HIS	C	81:ASN	N	6.61
1	C	140:PHE	C	157:PHE	N	6.54
1	A	66:HIS	C	79:PHE	N	6.04
1	B	672:ALA	C	687:VAL	N	5.40
1	A	672:ALA	C	687:VAL	N	5.33
1	C	672:ALA	C	687:VAL	N	5.14
1	B	141:LEU	C	155:SER	N	4.86

PRELIMINARY VALIDATION REPORT

6 Map visualisation (i)

This section contains visualisations of the EMDB entry D_1300026375. These allow visual inspection of the internal detail of the map and identification of artifacts.

No raw map or half-maps were deposited for this entry and therefore no images, graphs, etc. pertaining to the raw map can be shown.

6.1 Orthogonal projections (i)

6.1.1 Primary map

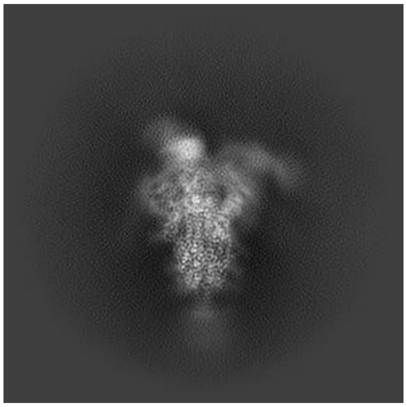

X

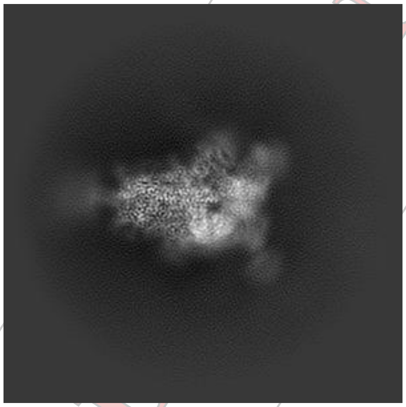

Y

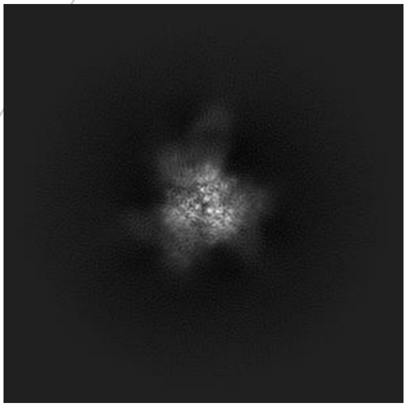

Z

The images above show the map projected in three orthogonal directions.

6.2 Central slices (i)

6.2.1 Primary map

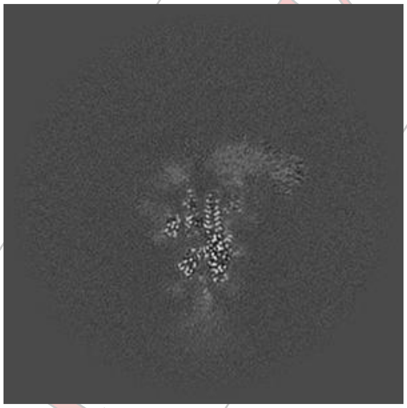

X Index: 200

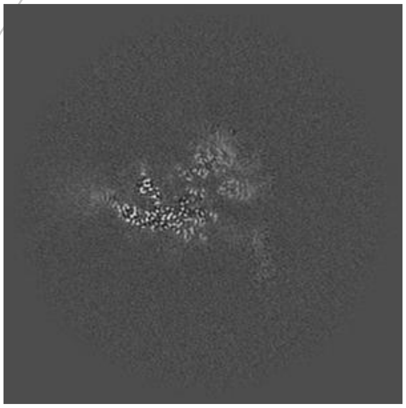

Y Index: 200

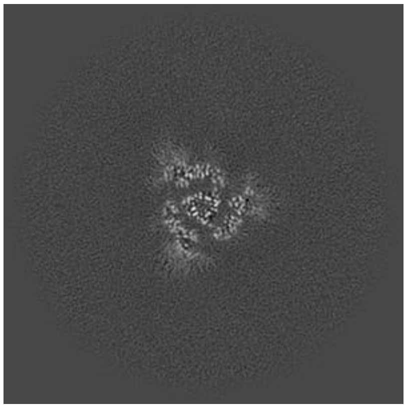

Z Index: 200

PRELIMINARY VALIDATION REPORT

The images above show central slices of the map in three orthogonal directions.

6.3 Largest variance slices (i)

6.3.1 Primary map

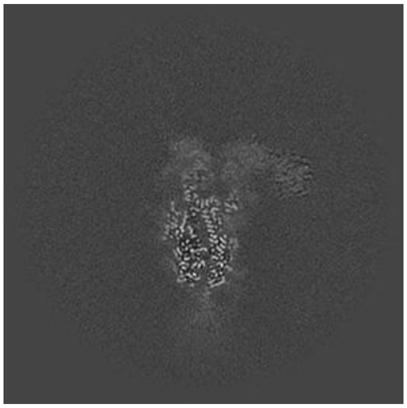

X Index: 211

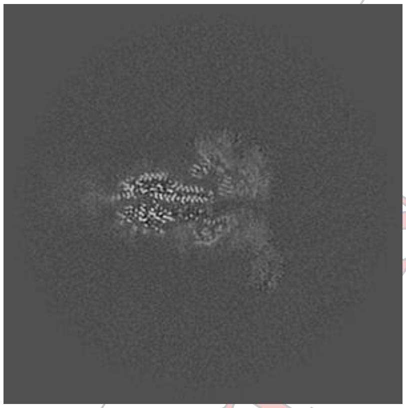

Y Index: 192

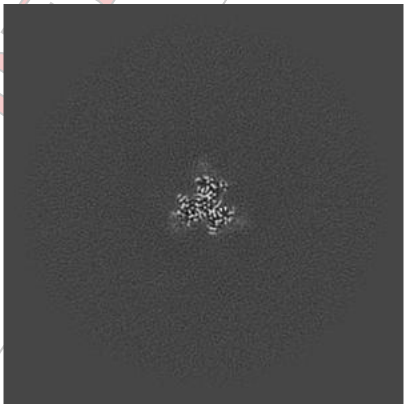

Z Index: 153

The images above show the largest variance slices of the map in three orthogonal directions.

6.4 Orthogonal surface views (i)

6.4.1 Primary map

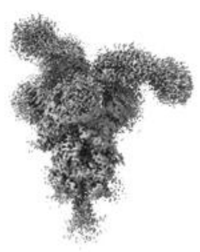

X

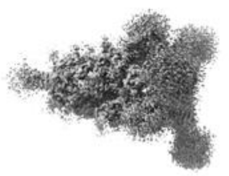

Y

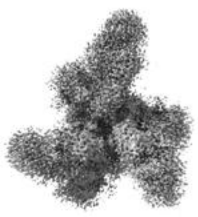

Z

The images above show the 3D surface view of the map at the recommended contour level 0.177. These images, in conjunction with the slice images, may facilitate assessment of whether an appropriate contour level has been provided.

PRELIMINARY VALIDATION REPORT

6.5 Mask visualisation (i)

This section was not generated. No masks/segmentation were deposited.

PRELIMINARY VALIDATION REPORT

7 Map analysis (i)

This section contains the results of statistical analysis of the map.

7.1 Map-value distribution (i)

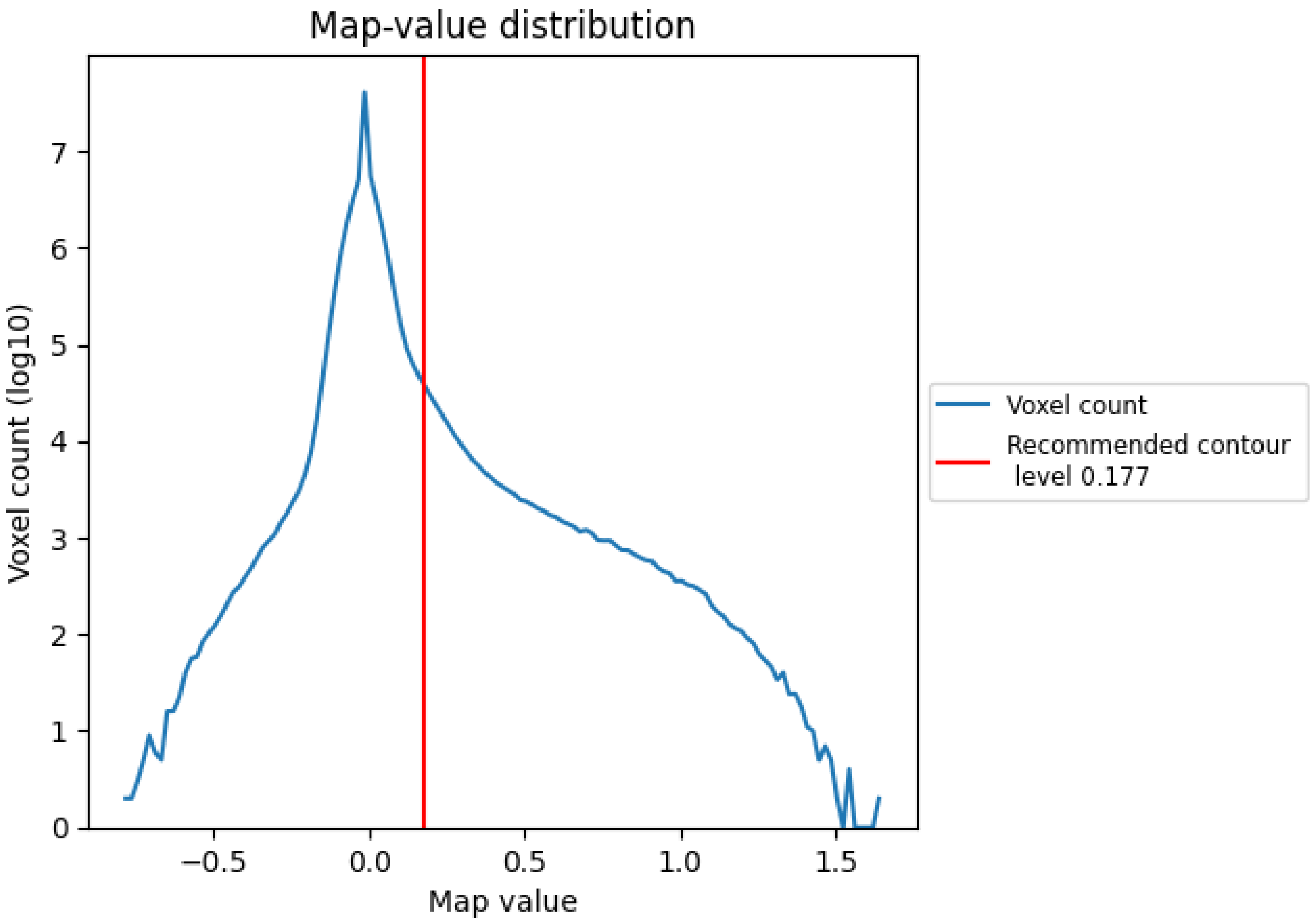

The map-value distribution is plotted in 128 intervals along the x-axis. The y-axis is logarithmic. A spike in this graph at zero usually indicates that the volume has been masked.

PRELIMINARY REPORT

7.2 Volume estimate ⓘ

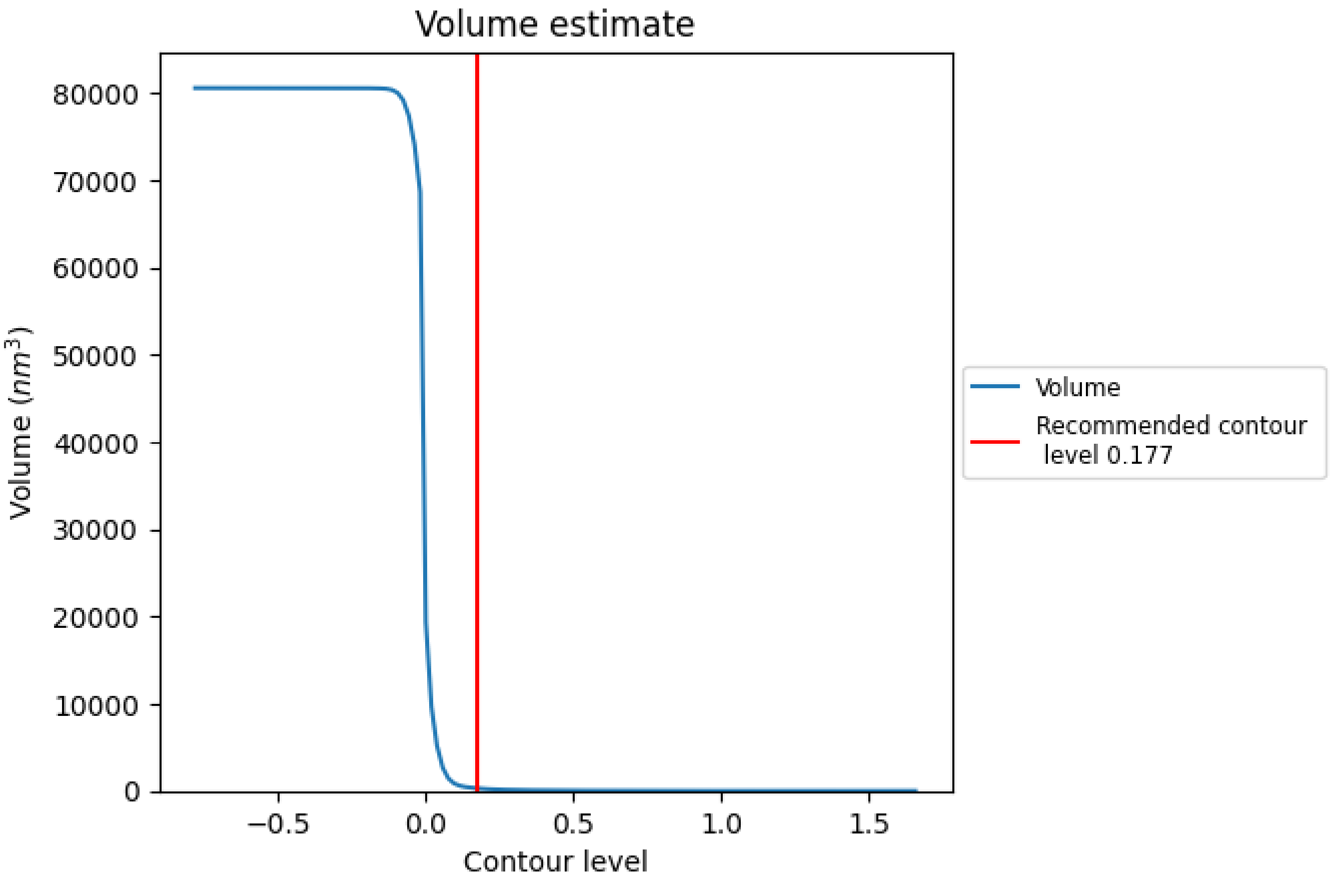

The volume at the recommended contour level is 285 nm^3; this corresponds to an approximate mass of 257 kDa.

The volume estimate graph shows how the enclosed volume varies with the contour level. The recommended contour level is shown as a vertical line and the intersection between the line and the curve gives the volume of the enclosed surface at the given level.

PRELIMINARY VA

7.3 Rotationally averaged power spectrum ⓘ

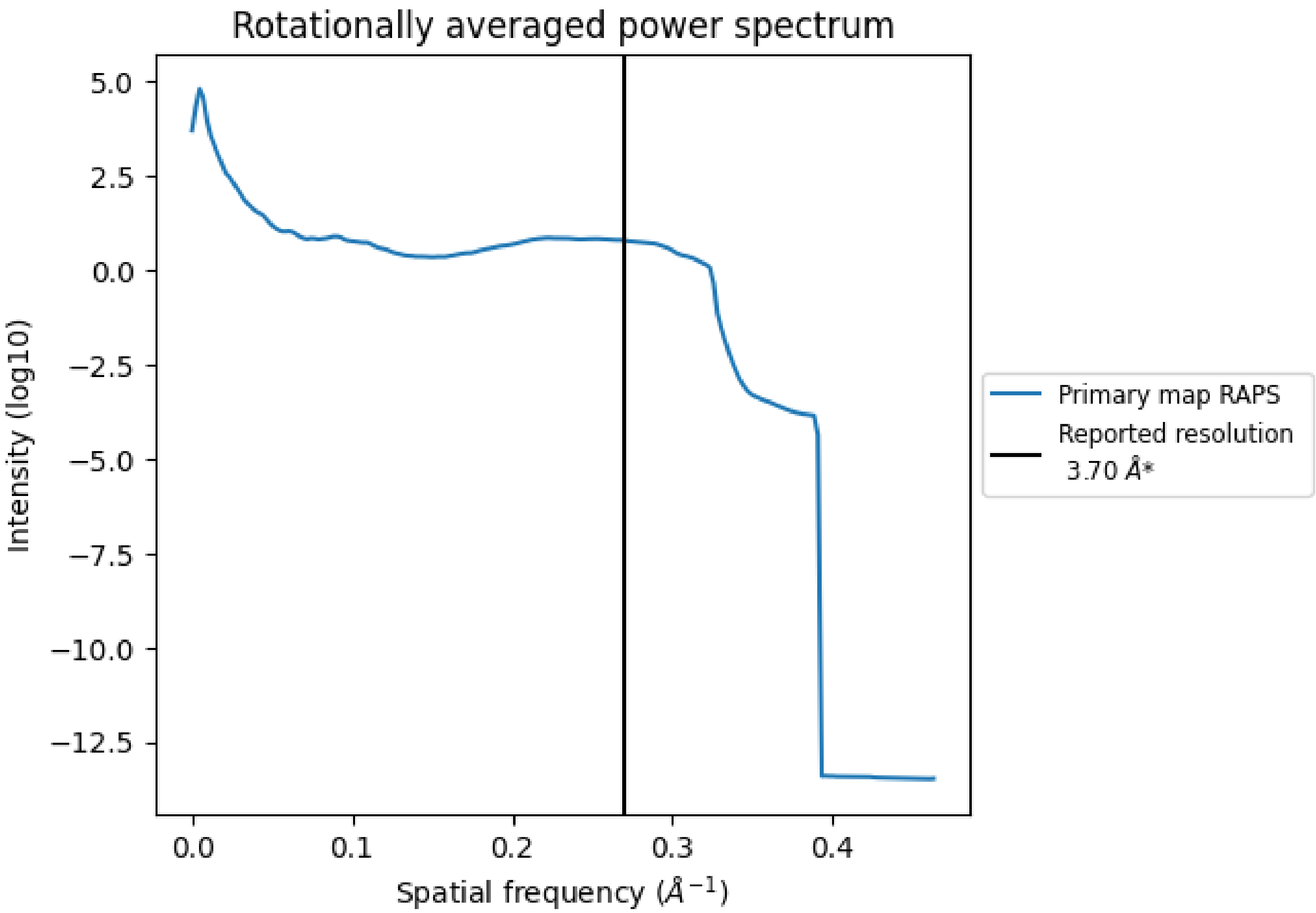

*Reported resolution corresponds to spatial frequency of 0.270 Å^{-1}

PRELIMINARY

8 Fourier-Shell correlation (i)

This section was not generated. No FSC curve or half-maps provided.

PRELIMINARY VALIDATION REPORT

9 Map-model fit

This section contains information regarding the fit between EMDB map D_1300026375 and PDB model D_1300026375. Per-residue inclusion information can be found in section 3 on page 8.

9.1 Map-model overlay

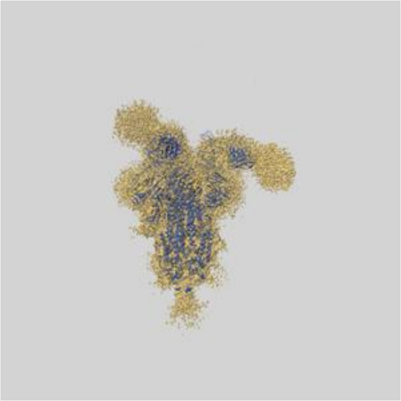

X

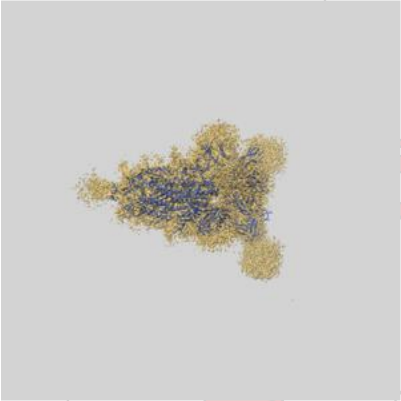

Y

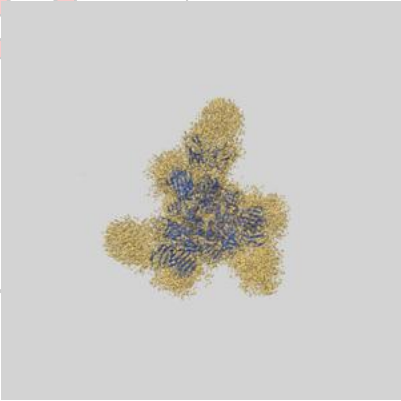

Z

The images above show the 3D surface view of the map at the recommended contour level 0.177 at 50% transparency in yellow overlaid with a ribbon representation of the model coloured in blue. These images allow for the visual assessment of the quality of fit between the atomic model and the map.

PRELIMINARY VALIDATION REPORT

9.2 Atom inclusion (i)

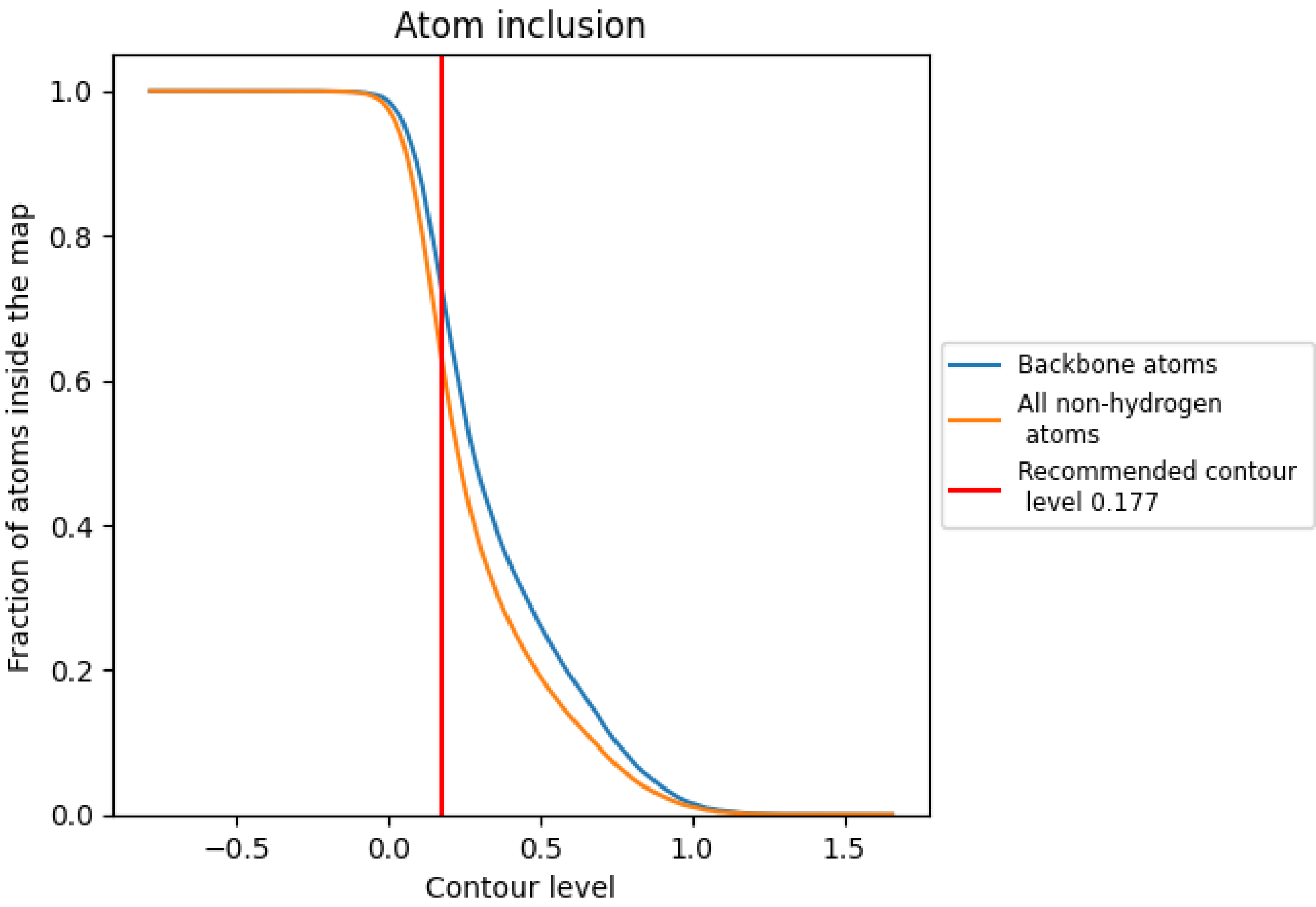

At the recommended contour level, 73% of all backbone atoms, 63% of all non-hydrogen atoms, are inside the map.

PRELIMINARY
